# CD200^+^ fibroblasts prime the immunoregulatory function of granulocytic myeloid-derived suppressor cells in inflammatory arthritis

**DOI:** 10.1038/s41467-026-78351-w

**Published:** 2026-10-13

**Authors:** Mario R. Angeli, Christian Schütz, Hashem Schmidt, Richard Demmler, Jiyang Chang, Hannah Labinsky, Maria Gabriella Raimondo, Brikena Lalazi, Katerina Stergioula, Kaiyue Huang, Vladyslav Fedorchenko, Mohan Li, Zoltán Winter, Styliani Tsiami, Arnd Lienert, Sebastian Rütten, Stefanie Weber, Markus Luber, Tobias Bäuerle, Reza Bahrami, Heinrich Boehm, Georg Schett, Simon Rauber, Xenofon Baraliakos, Andreas Ramming

**Affiliations:** 1 Department of Internal Medicine 3 - Rheumatology and Immunology, Friedrich-Alexander-Universität (FAU) Erlangen-Nürnberg and Universitätsklinikum Erlangen, Erlangen, Germany; 2 Deutsches Zentrum für Immuntherapie (DZI), Friedrich-Alexander-Universität (FAU) Erlangen-Nürnberg and Universitätsklinikum Erlangen, Erlangen, Germany; 3 Ruhr-Universität Bochum, Rheumazentrum Ruhrgebiet, Herne, Germany; 4 Department of Internal Medicine 2, Rheumatology and Clinical Immunology, Universitätsklinikum Würzburg, Würzburg, Germany; 5 Institute of Radiology, University Hospital, Friedrich-Alexander-Universität (FAU) Erlangen-Nürnberg and Universitätsklinikum Erlangen, Erlangen, Germany; 6 Center for Orthopedics and Traumatology, St. Anna Hospital, Herne, Germany; 7 Department of Radiology, Johannes-Gutenberg-Universität Mainz and Universitätsmedizin Mainz, Mainz, Germany; 8 Department for Spinal Surgery, Zentralklinik Bad Berka, Bad Berka, Germany

**Keywords:** Chronic inflammation, Autoimmunity, Innate immunity

## Abstract

Granulocytic myeloid-derived suppressor cells (G-MDSCs) are immunoregulatory cells of the neutrophil lineage. Here, we show that immunoregulation by G-MDSCs is imprinted by the joint tissue microenvironment in patients with axial spondyloarthritis and in animal models of inflammatory arthritis. We demonstrate that the suppressive function of G-MDSCs is significantly enhanced in the joint tissue, but not in the circulation. Analysis of public single-cell RNA sequencing datasets was consistent with *Cd200r1*^high^ *Hmgb2*^+^ precursors differentiating into G-MDSCs following interaction with *Cd200*^+^ fibroblasts. In hTNFtg/*Il23mc* mice, treatment with a CD200R1 agonist promoted an immunoregulatory signature associated with PD-L1 expression, whereas CD200 blockade reversed the phenotype. Following adoptive transfer of G-MDSC precursor cells into arthritic mice, administration of a CD200-blocking antibody impaired the differentiation of PD-L1^+^ G-MDSCs and exacerbated disease severity. These findings reveal a tissue-intrinsic program that facilitates resolution of inflammation through fibroblast-myeloid crosstalk that enhances G-MDSC function.

## Abstract (web summary)

Granulocytic myeloid-derived suppressor cells (G-MDSCs) are a subset of myeloid cells with immunoregulatory function. Here, the authors demonstrate that CD200-expressing fibroblasts drive the differentiation of neutrophil precursors into suppressive PD-L1^+^ G-MDSCs in the local joint microenvironment in inflammatory arthritis.

## Introduction

Among the myeloid lineage, a unique population of immature myeloid cells with immune regulatory properties has emerged^1–3^. Initially identified in mice, granulocytic myeloid-derived suppressor cells (G-MDSCs) share phenotypic similarities to neutrophils^4,5^, but exhibit distinct functional behaviors with predominant immunosuppressive properties in response to tumor-derived signals^6^. They are recognized for their ability to modulate immune responses, particularly their role in suppressing T cell activation and promoting tumor progression^3,5^. In humans, G-MDSCs are characterized by the surface markers HLA-DR^−^ CD11b^+^ CD33^+^ CD15^+^ and can be distinguished from monocytic myeloid-derived suppressor cells (M-MDSCs), which additionally express CD14 but no CD15. In mice, G-MDSCs are identified by the expression of CD11b^+^ Ly6G^+^ Ly6C^low^, whereas M-MDSCs are defined as CD11b^+^ Ly6G^−^ Ly6C^+^ cells^2,3^.

While G-MDSCs have been extensively studied in the context of cancer, revealing their powerful roles in tumor evasion and progression^7^, their role in immune-mediated inflammatory diseases, such as arthritis, is largely unexplored, which is surprising as the pathophysiology of these diseases is based on sustained inflammation.

The major forms of inflammatory arthritis in humans are rheumatoid arthritis (RA), psoriatic arthritis (PsA) and axial spondyloarthritis (axSpA). These diseases, although uniformly based on uncontrolled sustained inflammation, differ substantially in the nature of the affected target tissues (synovial membrane in RA^8^, peripheral tendon insertions in PsA^9^, vertebral bodies in axSpA^10^) as well as in the cytokines that drive the inflammatory process. These tissue-based differences may not only pertain to proinflammatory pathways but may also affect the immune regulatory pathways in these diseases. In contrast to cancer, where G-MDSCs support tumor growth by creating an immunosuppressive tissue environment, their suppressive function could act as anti-inflammatory in arthritis by shaping a regulatory tissue environment.

In this study, we have investigated the suppressive capacity of G-MDSCs in arthritis and found that it is primed by resident fibroblasts in the tissue expressing CD200. In arthritis, particularly axSpA, this tissue priming function of G-MDSCs is diminished, thereby losing their potential to control inflammation.

## Results

### Frequency of G-MDSCs correlates with disease activity in axSpA patients

**Fig. 1:**
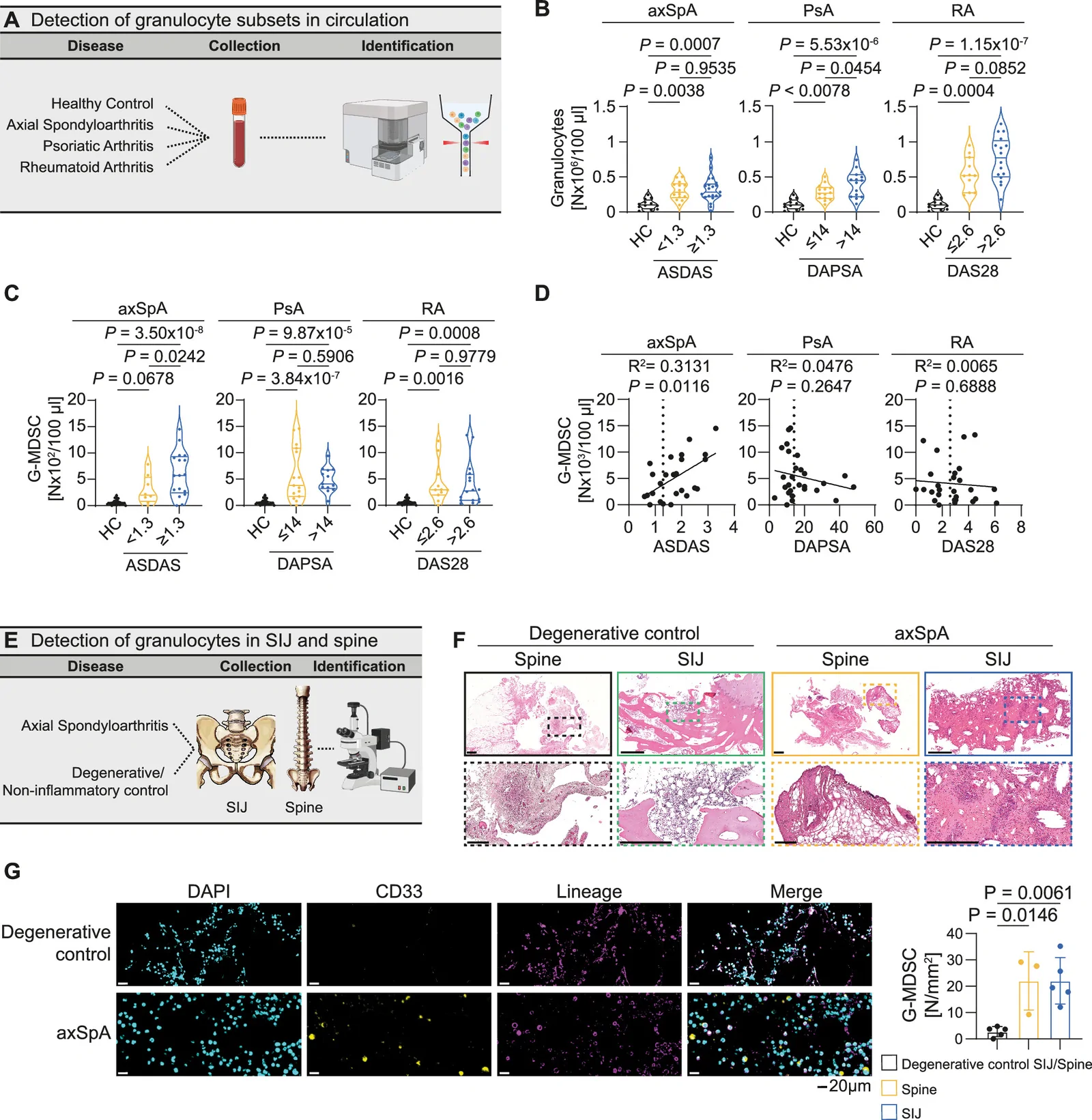
G-MDSCs are increased in peripheral blood and the tissue from patients with axial spondyloarthritis. **A** Workflow to detect granulocytes and suppressive innate cells from peripheral blood. **B** Quantification of blood granulocytes via flow cytometry of patients with different forms of arthritis separated by individual disease activity and healthy controls (HC; *n* = 12); Axial spondyloarthritis (axSpA, *n* = 35) according to axial spondyloarthritis disease activity score (ASDAS), psoriatic arthritis (PsA; *n* = 29) according to disease activity in psoriatic arthritis score (DAPSA) and rheumatoid arthritis (RA; *n* = 27) according to disease activity score 28 (DAS28). Violins show the median and quartiles. Quantification (**C**) and simple linear regression analysis (**D**) of blood granulocytic myeloid-derived suppressor cells (G-MDSCs) via flow cytometry in HC (*n* = 29), axSpA (*n* = 25), PsA (*n* = 28) and RA (*n* = 27) are separated by individual disease activity score. Violins show the median and quartiles. **E** Workflow to detect G-MDSCs in axial joint tissue of axSpA patients. Schematic spine and sacroiliac joint (SIJ) were created with Adobe Firefly. **F** Representative images of hematoxylin & eosin staining of inflamed tissue of SIJ and the spine of axSpA patients (*n* = 8; 3 spine, 5 SIJ) and degenerative controls (*n* = 5; 3 spine, 2 SIJ), representative of independent stainings with similar results. Scale bars are 500 μm (top) and 250 μm (bottom). **G** Representative images of immunofluorescence staining and quantification of G-MDSCs as CD33^+^Lin^−^ cells in IF stained tissues from degenerative controls (spine and SIJ, *n* = 3, 2) and spine and SIJ from axSpA patients (*n* = 3, 5). Lineage cocktail (Lin) contains HLA-DR, CD3, CD20, CD56, CD11c, CD123, CD14 and CD16. Bar plot shows Mean ± SD. Statistical significance was tested using two-sided unpaired one-way ANOVA with Tukey post hoc test (**B**–**C**, **G**). Illustration elements (**A**, **E**) were created in BioRender [Ramming, A. (2026) https://BioRender.com/5fy4lsh]. Additionally, schematic representations of the sacroiliac joint and spine in (**E**) were generated using Adobe Firefly. Source data are provided as a Source data file.

To investigate granulocytic suppressor cells in inflammatory joint diseases, we collected and analysed blood samples from patients with axSpA (*n* = 35), PsA (*n* = 29), RA (*n* = 27) as well as healthy controls without arthritis (HC, *n* = 29, Figs. 1A and S1A, Table 1). Total granulocyte counts were found to be elevated across all three forms of arthritis when compared to healthy controls (Fig. 1B), consistent with prior studies^11,12^. We next examined suppressive myeloid subsets, including eosinophils, granulocytic myeloid-derived suppressor cells (G-MDSCs), as well as monocytic myeloid-derived suppressor cells (M-MDSCs).

**Table 1:** Patient characteristics for peripheral blood analysis

Parameter	Healthy (*n* = 29)	axSpA (*n* = 35)	PsA (*n* = 29)	RA (*n* = 27)
Age, years, mean (s.d.)	29.2 (4.6)	42.7 (11.9)	55.9 (13.1)	59.8 (11.4)
Female/Male, *n*/*n*	18/11	6/29	14/15	19/8
Disease Duration, years, mean (s.d.)	-	9.2 (10.0)	8.0 (5.6)	1.6 (1.5)
DAS28, mean (s.d.)	-	-	-	2.7 (1.5)
DAPSA, mean (s.d.)	-	-	18.8 (15.9)	-
ASDAS, mean (s.d.)	-	1.9 (1.2)	-	-
BASDAI, mean (s.d.)	-	2.9 (2.2)	-	-
BASFI, mean (s.d.)	-	2.7 (2.5)	-	-
Rheumatoid Factor, *n* (%)	-	0 (0.0)	0 (0.0)	12 (44.4)
Anti-cyclic citrullinated peptide antibodies, *n* (%)	-	0 (0.0)	0 (0.0)	12 (44.4)
C-reactive protein, mg dl^−1^, mean (s.d.)	-	0.8 (1.2)	1.1 (2.6)	8.0 (14.1)
**Treatment**				
NSAIDs, *n*	-	16	0	1
Glucocorticoids, *n*	-	-	0	1
cDMARD, *n*	-	-	14	13
TNFi, *n*	-	16	6	9
JAKi, *n*	-	1	1	0
IL-17i, *n*	-	2	4	0
IL-23i, *n*	-	-	4	0
CTLA4-Ig, *n*	-	-	0	2
IL-6i, *n*	-	-	0	1

*ASDAS* Axial Spondyloarthritis Disease Activity Score, *axSpA* axial spondyloarthritis, *BASDAI* Bath Ankylosing Spondylitis Disease Activity Index, *BASFI* Bath Ankylosing Spondylitis Functional Index, *cDMARD* conventional Disease-Modifying Antirheumatic Drug, *CTLA4* cytotoxic T-lymphocyte-associated protein 4 immunoglobulin fusion protein, *DAS28* Disease Activity Score in 28 joints, *DAPSA* Disease Activity Index for Psoriatic Arthritis, *IL-17i* interleukin-17 inhibitor, *IL-23i* interleukin-23 inhibitor, *IL-6i* interleukin-6 inhibitor, *JAKi* Janus kinase inhibitor, *n* number of individuals, *NSAIDs* non-steroidal anti-inflammatory drugs, *PsA* psoriatic arthritis, *RA* rheumatoid arthritis, *s.d.* standard deviation, *TNFi* tumor necrosis factor inhibitor.

While eosinophils were significantly enriched in RA patients alone (Fig. S1B), G-MDSCs and M-MDSCs were elevated in patients with RA, PsA and axSpA compared to healthy donors (Figs. 1C and S1C). Moreover, in axSpA patients, circulating G-MDSCs, but not M-MDSCs, exhibited a positive correlation with disease activity (Figs. 1D and S1D).

This unexpected, paradoxical signal of increased G-MDSCs among patients with active axSpA prompted us to further investigate the role of G-MDSCs at the site of inflammation. We conducted an analysis of G-MDSCs in axial joint biopsies from axSpA patients. We obtained CT-guided biopsies from the sacroiliac joints (SIJ) and biopsies from facet joints during spine surgeries (Table 2), using biopsies from patients with degenerative axial disease as controls (Fig. 1E, F). Immunofluorescence imaging revealed a significant accumulation of G-MDSCs in the axial tissues of axSpA patients (Fig. 1G), aligning with their elevated presence in the circulation. This accumulation suggests impaired regulatory function and may reflect a compensatory, yet ultimately ineffective, response to ongoing inflammation.

**Table 2:** Patient characteristics for axial tissue analysis, related to Fig. 1G

Parameter	axSpA (*n* = 8)	Degenerative/Non-inflammatory control (*n* = 5)
Age, years, mean (s.d.)	42.6 (11.2)	65.4 (12.0)
Female/Male, *n*/*n*	1/7	3/2
Biopsy Location	3 Spine/5 SIJ	3 Spine/2 SIJ
BASDAI, mean (s.d.)	6.7 (1.0)	-
ASDAS, mean (s.d.)	3.7 (0.7)	-
Treatments	5 TNFi, 1 IL-17i, 2 Bionaïve	-

*ASDAS* Axial Spondyloarthritis Disease Activity Score, *BASDAI* Bath Ankylosing Spondylitis Disease Activity Index, *IL-17i* interleukin-17 inhibitor, *n* number of individuals, *SIJ* sacroiliac joint, *TNFi* tumor necrosis factor inhibitor, *axSpA* axial spondyloarthritis, *s.d.* standard deviation.

### Suppressive CXCR2^−^CD101^−^ G-MDSCs emerge in experimental axSpA

**Fig. 2:**
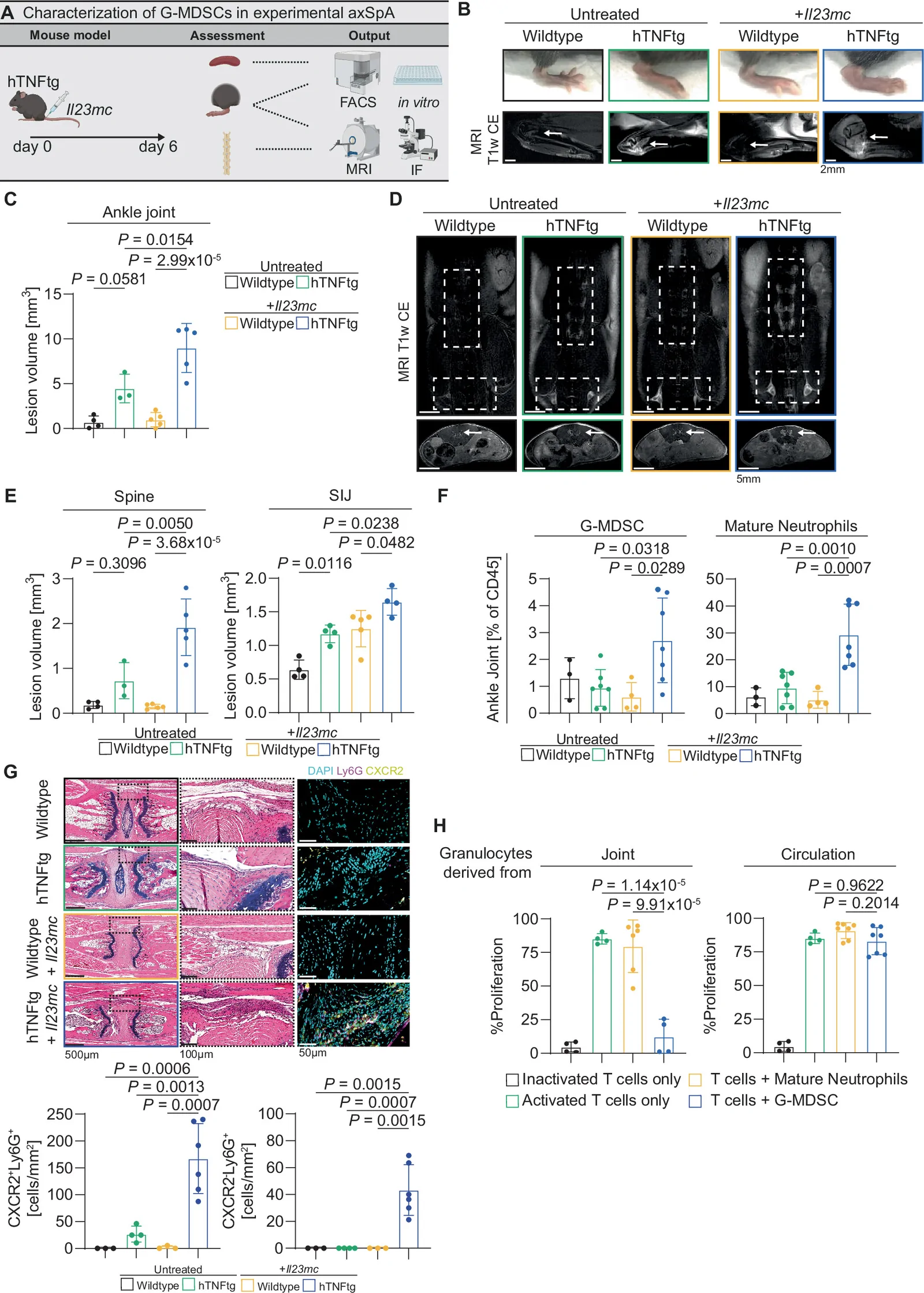
Suppressive G-MDSCs are tissue-specific in experimental axSpA. **A** Workflow in experimental axial axSpA. **B**–**E** Inflammation in joint, spine and sacroiliac joints (SIJ) after 6 days. Representative images of hind paws and MRI scans of ankle joints using T1-weighted contrast-enhanced sequences (T1w CE, **B**) and lesion volume from MRI of ankle joints (**C**, T1w CE volume) from wildtype mice, hTNFtg mice, wildtype mice treated with *Il23mc* and hTNFtg mice treated with *Il23mc* (*n* = 4, 3, 5, 5 mice, Mean ± SD, pooled from two independent experiments). Representative MRI scans (**D**) of the spine (*n* = 4, 3, 5, 5 mice, pooled from two independent experiments) and SIJ (*n* = 4, 4, 5, 4 mice, pooled from two independent experiments) with respective quantification of the lesion volume (**E**, T1w CE volume) from wildtype mice, hTNFtg mice, wildtype mice treated with *Il23mc* and hTNFtg mice treated with *Il23mc*. Mean ± SD. The dashed-line boxes and arrows highlight the regions of contrast enhancement in spine and SIJ. Pooled from two independent experiments. **F** Frequency of G-MDSCs and mature neutrophils from ankle joints from wildtype mice, hTNFtg mice, wildtype mice treated with *Il23mc* and hTNFtg mice treated with *Il23mc* after 6 days (*n* = 3, 7, 4, 7 mice) analysed by flow cytometry. G-MDSCs were defined as viable CD45⁺ CD11b⁺ Ly6C^low^ Ly6G⁺ CXCR2⁻ CD101⁻ Lineage⁻ cells (Lineage = CD3, B220, CD49b, CD11c, SiglecF). Mature neutrophils were identified as viable CD45⁺ CD11b⁺ Ly6C^low^ Ly6G⁺ CXCR2⁺ CD101⁺ Lineage⁻ cells. Gating strategy shown in Fig. S2A. Mean ± SD, pooled from three independent experiments. **G** Localization and quantification of G-MDSCs (CXCR2^−^Ly6G^+^) and mature neutrophils (CXCR2^+^Ly6G^+^) in axial tissue from wildtype mice, hTNFtg mice, wildtype mice treated with *Il23mc* and hTNFtg mice treated with *Il23mc* (*n* = 3, 4, 3, 6 mice) detected by immunofluorescence imaging. Mean ± SD, pooled from three independent experiments. **H** Proliferation of T cells determined by CFSE after culture with mature neutrophils or G-MDSCs derived from ankle joint (*n* = 6, 4 mice) or circulation (*n* = 7 both) of hTNFtg mice treated with *Il23mc*. Controls are IL-2 stimulated T cells only without CD3/CD28 and IL-2 stimulated T cells only with CD3/CD28 (*n* = 4 mice both). Mean ± SD, pooled from three independent experiments. Statistical significance was tested using two-sided unpaired one-way ANOVA with Tukey post hoc test. Illustration elements (**A**) were created in BioRender [Ramming, A. (2026) https://BioRender.com/5fy4lsh]. Source data are provided as a Source data file.

Functional assessment of G-MDSCs from human spine biopsies presents significant technical challenges. To circumvent this limitation and due to the well-established homology between the neutrophil compartments in mice and humans^13^, we examined the suppressive functionality of G-MDSCs in murine models of arthritis. We first utilized human TNF transgenic (hTNFtg) mice and wildtype littermates, both treated and untreated with murine IL-23 minicircle DNA (*Il23mc*) (Fig. 2A). Previous studies have shown that the combination of TNF and IL-23 induces severe periarticular, axial, entheseal and synovial inflammation closely mirroring the phenotype of spondyloarthritis (Fig. 2B–E)^14–16^. This process is associated with significant increase in circulating granulocytes, mature neutrophils and G-MDSCs, paralleling observations in the axSpA patient cohort (Fig. S2A–C). Interestingly, G-MDSCs also accumulated in the sacroiliac, spine and ankle joints of hTNFtg/*Il23mc* mice, locations typically involved in axSpA (Figs. 2F, G and S2D). The inflammatory infiltrates in the lumbar and sacroiliac joints included both pro-inflammatory neutrophils and G-MDSCs, indicating an interaction between pathogenic and regulatory myeloid populations within the axial structures critical to axSpA pathophysiology.

To evaluate the suppressive capacity of G-MDSCs, we performed fluorescence-activated cell sorting (FACS) to isolate G-MDSCs and mature neutrophils from joint tissues and the circulation of *Il23mc* treated hTNFtg animals. We co-cultured naïve carboxyfluorescein succinimidyl ester (CFSE)-labeled CD4^+^CD25^−^ T cells (Fig. S2E) with either G-MDSCs or mature neutrophils. While mature neutrophils demonstrated no suppressive effects as expected (Figs. 2H and S2F), G-MDSCs exhibited suppressive activity without affecting T cell viability (Fig. S2G). Notably, this effect was observed exclusively with cells derived from joint tissues, but not the circulation, suggesting that G-MDSCs undergo tissue-specific priming that enhances their regulatory capabilities.

### G-MDSCs arise from *Hmgb2*^+^ precursors across inflammatory conditions in mice

The observation that G-MDSCs acquire suppressive capacity specifically within tissues supports the idea of local environmental cues guiding their differentiation and functional specialization. To better understand the cellular programs and developmental trajectories underlying this tissue-induced immunoregulatory phenotype, we analysed a publicly available cross-tissue neutrophil atlas (viable CD11b⁺ Ly6G⁺, GSE165276^17^) covering five inflammatory and homeostatic conditions: arthritic blood and joint from the K/BxN serum-transfer arthritis model, IL-1β-induced peritonitis, IL-1β-induced pneumonitis and healthy blood.

**Fig. 3:**
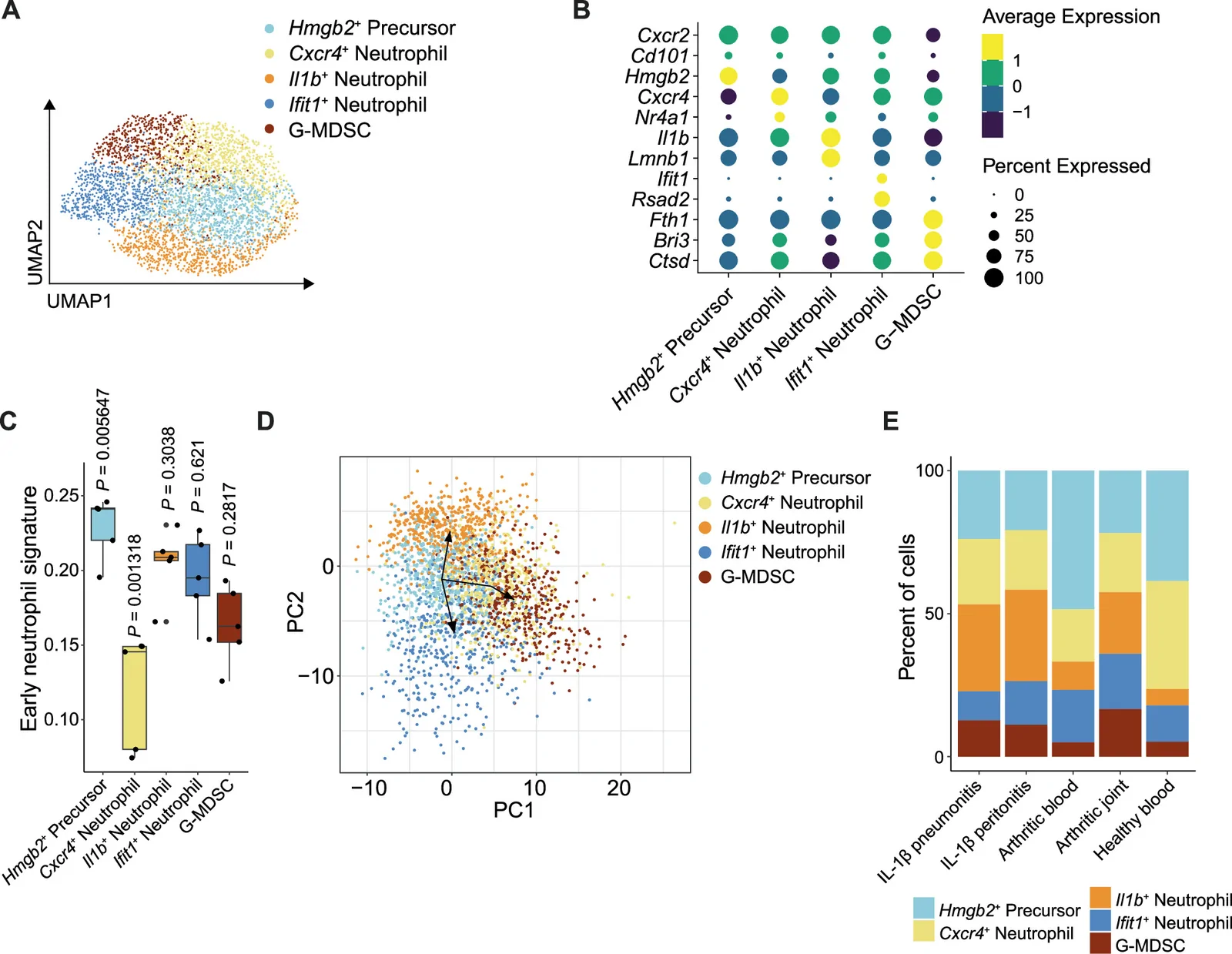
G-MDSCs polarize from *Hmgb2*^+^ precursors across inflammatory conditions in mice. **A** Uniform manifold approximation and projection (UMAP) for visualization of the integrated single-cell mRNA sequencing (scRNA-seq) dataset (Viable CD11b^+^Ly6G^+^, GSE165276^17^) showing the heterogeneity of neutrophils (*n* = 5, one per tissue/condition: IL-1β pneumonitis, IL-1β peritonitis, arthritic blood, arthritic joint, healthy blood). **B** Expression of the most relevant marker genes among each neutrophil subset as dot plot from (**A**). **C** Early neutrophil score^17^ of annotated neutrophil clusters testing the maturity state. Statistical significance was tested using two-sided one-vs-rest Wilcoxon rank sum test and corrected for multiple testing using BH. Box plots show the median (centre line), the 25th and 75th percentiles (box bounds) and the minimum and maximum values (whiskers). **D** Trajectory analysis of neutrophil subsets by Slingshot^22^ visualized on the first two principal components. **E** Bar plot comparing the percentage of cells between inflammatory and healthy conditions. Source data are provided as a Source data file.

Utilizing Uniform Manifold Approximation and Projection (UMAP) clustering in the neutrophil atlas, we identified five distinct neutrophil subsets: G-MDSCs, *Ifit1*^+^ neutrophils, *Il1b*^+^ neutrophils, *Cxcr4*^+^ neutrophils and a *Hmgb2*^+^ subset (Fig. 3A).

The G-MDSC cluster was defined by high expression of canonical immunosuppressive and MDSC-associated genes, including *Fth1* and *Ctsd*^18–21^ and by low expression of *Cxcr2* and *Cd101* (Fig. 3B), consistent with the previously reported CXCR2⁻CD101⁻ phenotype of murine G-MDSCs. *Hmgb2*^+^ cells displayed the highest early neutrophil score^17^ (Fig. 3C, Table S2), identifying them as early-stage precursors with differentiation potential. This was supported by trajectory modeling^22^, which revealed a developmental path from *Hmgb2*⁺ precursors toward G-MDSCs and the remaining neutrophil subsets (Fig. 3D).

Across the tissues and inflammatory models, *Hmgb2*⁺ precursors were prevalent in the circulation, whereas G-MDSCs were rare in blood but highly enriched in inflamed tissues (Fig. 3E), consistent with local differentiation rather than recruitment of preformed suppressor cells.

Together, these data identify *Hmgb2*⁺ cells as precursors with the potential to give rise to immunoregulatory G-MDSCs. The same continuum was present across distinct inflammatory conditions, pointing to local tissue cues in the inflamed microenvironment as drivers of their functional conversion.

### A CD200^+^ fibroblast niche for G-MDSC differentiation in mice and humans

**Fig. 4:**
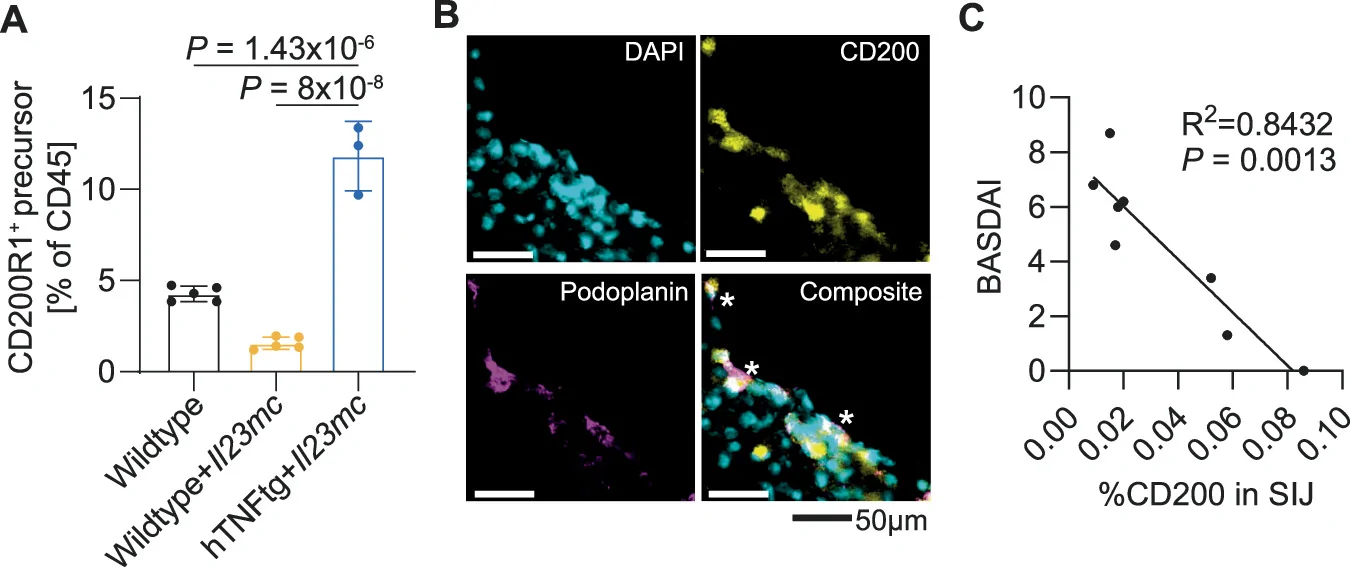
A CD200⁺ fibroblast niche for G-MDSC differentiation in mice and humans. **A** Abundance of CD200R1^+^ precursors (Viable SSC^high^CD4^−^CD90^−^CD170^−^CD11b^+^CD25^−^CD3^−^CD200R1^+^) in ankle joints of untreated wildtype mice, wildtype mice treated with *Il23mc* and hTNFtg mice treated with *Il23mc* analysed with flow cytometry (*n* = 5, 5, 3 mice, data are from one experiment). Mean ± SD. Representative image of immunofluorescence staining of CD200^+^Podoplanin^+^ stromal cells in human SIJ (**B**) with simple linear regression analysis (**C**) between the percentage of CD200⁺ cells in the sacroiliac joint (SIJ) and clinical disease activity of axial spondyloarthritis by Bath Ankylosing Spondylitis Disease Activity Index (BASDAI), with regression line, R² indicating goodness of fit and *P* value testing whether the slope differs from zero (*n* = 8 patients). Statistical testing: (**A**) two-sided unpaired one-way ANOVA with Tukey post hoc test. Source data are provided as a Source data file.

To identify the tissue-derived signals responsible for this conversion, we analysed a publicly available whole-tissue single-cell RNA-sequencing (scRNA-seq) dataset from inflamed ankle joints of mice with K/BxN serum-transfer arthritis (GSE253974^23^). Major cell types were identified based on established marker genes (Fig. S3A, B). Subclustering of the neutrophil compartment and reference mapping of our previous annotations recovered *Hmgb2*^+^ precursors and G-MDSCs (Fig. S3C, D). Trajectory analysis using CytoTRACE^24^ and CytoTRACE2^25^ confirmed *Hmgb2*⁺ cells as the least differentiated precursor population in this independent dataset (Fig. S3E–G). Using CellChat-based inference of ligand-receptor interactions^26^, we found that fibroblasts constitute the dominant communication partner for neutrophils in the inflamed joint environment (Fig. S3H, I). We therefore annotated fibroblast subsets according to our previous atlas using reference mapping (Fig. S3J, K), in which we had identified an immunoregulatory *Cd200*^+^ fibroblast subset that dampened arthritis^16,27^. Detailed analysis of intercellular interactions revealed a robust communication between *Cd200*^+^ fibroblasts and *Hmgb2*^+^ precursors (Fig. S3L). Consistent with this, *Hmgb2*^+^ precursors showed strong *Cd200r1* expression after signal imputation with Adaptively thresholded Low-Rank Approximation (ALRA)^28^ (Fig. S3M). Together, these analyses generated the hypothesis that *Cd200*^+^ fibroblasts direct *Hmgb2*^+^ precursors toward an anti-inflammatory fate via CD200/CD200R1 signaling. We next examined whether these precursors are present in the experimental axSpA model. Flow cytometry of ankle joints from hTNFtg mice treated with *Il23mc* confirmed CD200R1 expression on precursors and revealed an expansion in frequency of this population in arthritis compared with controls, indicating that inflammation amplifies the availability of CD200-responsive precursors (Figs. 4A and S3N).

To explore the clinical relevance of this pathway, we performed immunofluorescence staining on sacroiliac joint biopsies from axSpA patients and identified Podoplanin⁺CD200⁺ stromal cells, consistent with fibroblast identity in the biopsies (Fig. 4B, Table S1). Quantification of these cells revealed an inverse correlation between the frequency of CD200⁺ fibroblasts and patient-reported disease activity (Bath Ankylosing Spondylitis Disease Activity Index, BASDAI), suggesting that loss of tissue CD200 expression may contribute to persistent inflammation (Fig. 4C).

### CD200/CD200R1 signaling is required to sustain the immunoregulatory PD-L1⁺ G-MDSC phenotype in experimental axSpA

**Fig. 5:**
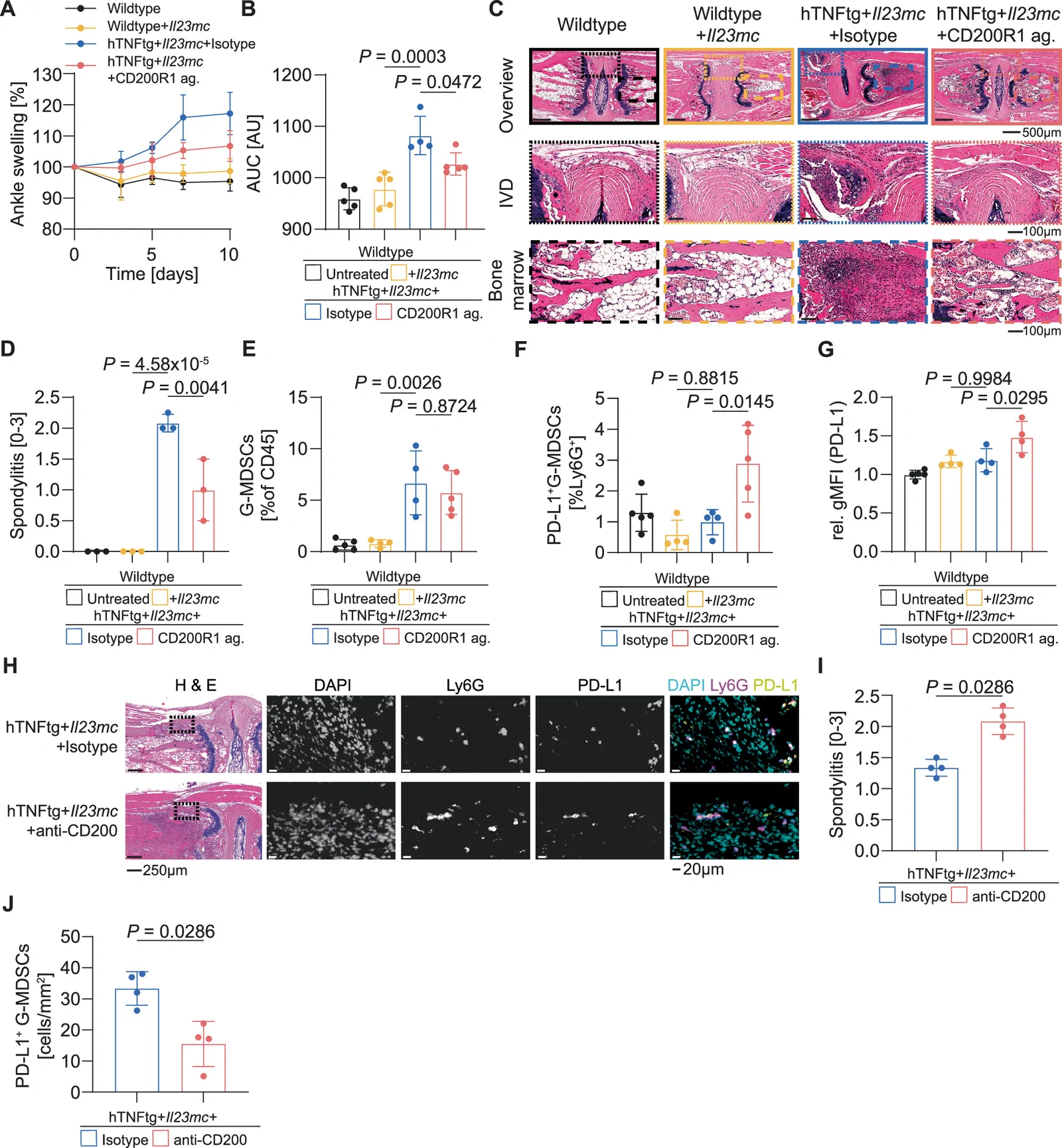
CD200/CD200R1 signaling is required to sustain the immunoregulatory PD-L1⁺ G-MDSC phenotype in experimental axSpA. Ankle swelling (**A**) with area under the curve analysis (**B**) of untreated wildtype mice, wildtype mice treated with *Il23mc*, hTNFtg mice treated with *Il23mc* in combination with either rat IgG2a isotype antibody or CD200R1 agonistic antibody (Mean ± SD; *n* = 5, 5, 4, 5 mice). Representative hematoxylin & eosin images of axial joints showing overview, intervertebral disc (IVD) and bone marrow (**C**) and semiquantitative scoring of spondylitis (**D**, *n* = 3 mice each). Mean ± SD, pooled from two independent experiments. **E** Abundance of G-MDSCs in ankle joints of untreated wildtype mice, wildtype mice treated with *Il23mc* and hTNFtg mice treated with *Il23mc* in combination with either rat IgG2a isotype antibody or CD200R1 agonistic antibody (*n* = 5, 4, 4, 5 mice). Shown is percentage of G-MDSCs (CXCR2^−^CD101^−^Ly6G^+^Ly6C^int^CD11b^+^Lineage^−^CD45^+^ cells) on total CD45^+^ cells. Lineage gate consists of CD3, B220, CD49b, CD11c, SiglecF/CD170. Gating strategy as shown in Fig. S2A. Mean ± SD, pooled from three independent experiments. Abundance of PD-L1^+^ G-MDSCs in ankle joints within the Ly6G^+^CD11b^+^ compartment (**F**) of untreated wildtype mice, wildtype mice treated with *Il23mc* and hTNFtg mice treated with *Il23mc* in combination with either rat IgG2a isotype antibody or CD200R1 agonistic antibody (*n* = 5, 4, 4, 5 mice) and PD-L1 expression of G-MDSCs as mean fluorescence intensity (**G**) normalized to untreated wildtype mice (Mean ± SD; *n* = 5, 4, 4, 4 mice, pooled from two independent experiments). Representative hematoxylin & eosin images and representative immunofluorescence images (DAPI, Ly6G, PD-L1, Composite) of inflamed areas in axial joints (**H**), semiquantitative scoring of spondylitis (**I**) and quantification of Ly6G^+^PD-L1^+ ^cells per mm^2^ (**J**) in axial joints of hTNFtg/*Il23mc* mice treated with rat IgG2a isotype control or anti-CD200 blocking antibody (OX-90) (*n* = 4, 4 mice, biological replicates). Scale bars, 250 μm (H&E) and 20 μm (immunofluorescence). Mean ± SD. Statistical testing: (**B**, **D**, **E**, **F**, **G)** two-sided unpaired one-way ANOVA with Tukey post hoc test and (**I**, **J**) two-sided Mann-Whitney *U* test. Source data are provided as a Source data file.

To test the functional relevance of this signaling axis in vivo, we treated hTNFtg/*Il23mc* mice with a CD200R1 agonist antibody (OX-110). This intervention significantly reduced inflammation in both axial and peripheral joints (Fig. 5A–D), confirming the therapeutic potential of CD200R1 signaling. Notably, CD200R1 agonistic treatment did not significantly alter the overall abundance of G-MDSCs in inflamed tissue (Fig. 5E), suggesting that the treatment modulates the functional phenotype rather than the numbers of these cells. To address this, we returned to the neutrophil atlas and subclustered G-MDSCs, which revealed a transcriptionally distinct *Cd274*⁺ (encoding programmed death-ligand 1, PD-L1) subpopulation enriched for immunosuppressive gene ontology biological processes (Fig. S4A–C).

We next assessed whether CD200R1 stimulation selectively affects the abundance of this PD-L1⁺ G-MDSC subpopulation. Notably, we observed a significant increase in the proportion of PD-L1⁺ G-MDSCs within the Ly6G^+^CD11b^+^ compartment following CD200R1 agonist antibody treatment, suggesting functional heterogeneity within the G-MDSCs (Figs. 5F and S4D). Additionally, PD-L1 expression levels (mean fluorescence intensity) were elevated on these cells compared to untreated controls (Fig. 5G). These data suggest that CD200R1 signaling induces a functional skewing of G-MDSCs toward an immunoregulatory PD-L1⁺ phenotype.

To directly test whether endogenous CD200/CD200R1 signaling is required to control disease, we treated hTNFtg/*Il23mc* mice with an anti-CD200 blocking antibody (OX-90). CD200 blockade aggravated inflammation in axial joints (Fig. 5H, I), demonstrating that endogenous CD200/CD200R1 signaling constrains disease severity. Accordingly, anti-CD200 blockade reduced the number of PD-L1^+^ G-MDSCs at the site of disease (Fig. 5J), consistent with impaired differentiation into the immunoregulatory phenotype. Together, these gain- and loss-of-function experiments demonstrate that CD200/CD200R1 signaling is required to sustain the immunoregulatory PD-L1^+^ G-MDSC phenotype and to limit inflammation in experimental axSpA.

### G-MDSCs acquire PD-L1-dependent suppressive function through CD200-driven differentiation in mice

**Fig. 6:**
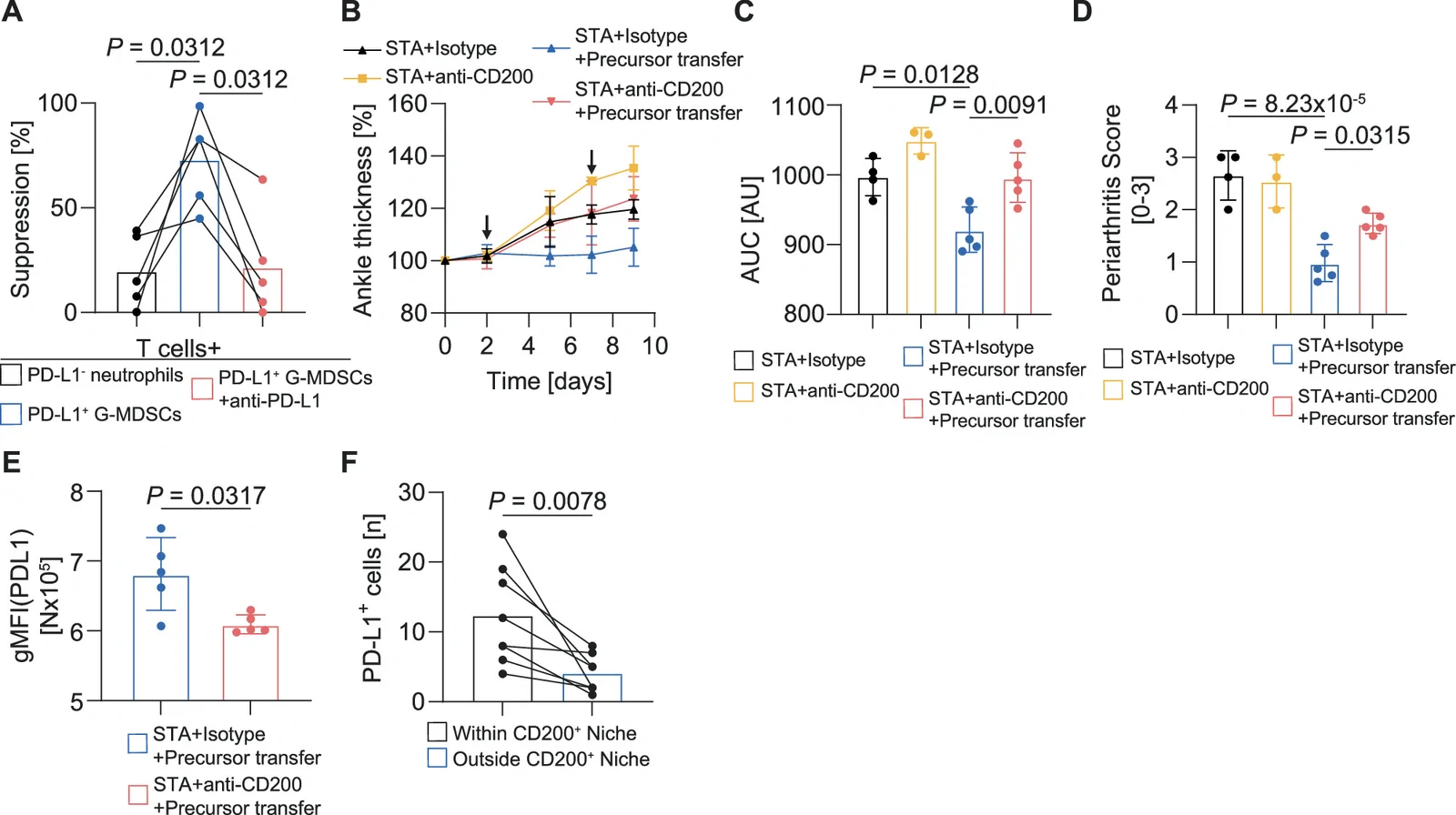
G-MDSCs acquire PD-L1-dependent suppressive function through CD200-driven differentiation in mice. **A** Proliferation of CFSE-labeled naïve CD4⁺ T cells co-cultured with bone marrow-derived Ly6G⁺ cells polarized with GM-CSF (PD-L1⁺), G-CSF (PD-L1⁻), or GM-CSF with anti-PD-L1 blocking antibody (*n* = 5 mice, pooled from three independent experiments). **B**–**D** Measurement of arthritis after serum transfer arthritis (STA) induction in the ankle joints (**B**) with area under the curve analysis (**C**) from arthritic mice treated with isotype control rat IgG2a or anti-CD200 blocking antibody (OX-90) without cell transfer, and arthritic mice adoptively transferred with CFSE-labeled CD177⁺Ly6G⁺ precursors under isotype control or anti-CD200 blockade (*n* = 4, 3, 5, 5 mice, data are from one experiment). Arrows in (**B**) indicate the timepoints of adoptive transfers. Mean ± SD. **D** Semiquantitative periarthritis score (0–3) of ankle joints (*n* = 4, 3, 5, 5 mice, data are from one experiment). Mean ± SD. **E** PD-L1 expression as mean fluorescence intensity of adoptively transferred CFSE^+^ cells recovered from inflamed joints of recipient mice (*n* = 5 mice per group, data are from one experiment). Mean ± SD. **F** Quantification of PD-L1⁺ cells located within versus outside the CD200⁺ niche in human axial joint sections (*n* = 8 patients, paired analysis). Statistical testing: (**C**, **D**) two-sided unpaired one-way ANOVA with Tukey post hoc test, (**A**) one-sided Wilcoxon matched-pairs signed-rank test, (**E**) two-sided Mann-Whitney *U* test and (**F**) two-sided Wilcoxon matched-pairs signed-rank test. Source data are provided as a Source data file.

To determine whether PD-L1 is functionally required for G-MDSC-mediated suppression, we polarized bone marrow-derived Ly6G^+^ cells with either granulocyte-macrophage colony-stimulating factor (GM-CSF) or granulocyte colony-stimulating factor (G-CSF). GM-CSF-polarized G-MDSCs upregulated PD-L1, consistent with previous reports^29^ and significantly suppressed T cell proliferation in vitro, whereas G-CSF-polarized PD-L1^−^ cells did not (Figs. 6A and S5A). Furthermore, anti-PD-L1 blockade was sufficient to abrogate the suppressive capacity of GM-CSF-polarized PD-L1^+^ G-MDSCs, demonstrating that PD-L1 is functionally required for immunosuppression rather than serving as a phenotypic marker. To explore whether PD-L1^+^ G-MDSCs are sufficient to ameliorate arthritis in vivo, we adoptively transferred PD-L1-expressing G-MDSCs into arthritic mice. The transfer significantly reduced joint swelling compared to controls, along with decreased leukocyte infiltration and preservation of joint structure (Fig. S5B–E).

To determine whether CD200 signaling directly instructs the differentiation of precursors into G-MDSCs, we adoptively transferred fluorescently labeled CD177^+^Ly6G^+^ precursor cells corresponding to *Hmgb2*^+^ precursors^17^ into arthritic mice treated with anti-CD200 blocking antibody or isotype control. Anti-CD200 blockade impaired PD-L1⁺ G-MDSC generation from transferred precursors and abolished their therapeutic benefit, whereas isotype antibody-treated recipients showed significantly reduced arthritis (Figs. 6B–E and S5F, G). These data demonstrate that CD200/CD200R1 signaling is necessary for precursors to differentiate into PD-L1⁺ G-MDSCs with immunosuppressive function in vivo.

Translating these findings to human disease, spatial analyses of human sacroiliac joints revealed that PD-L1⁺ cells were preferentially located near CD200⁺ cells, identifying a CD200⁺-centered niche that may instruct local G-MDSC polarization (Fig. 6F, Table S1).

## Discussion

**Fig. 7:**
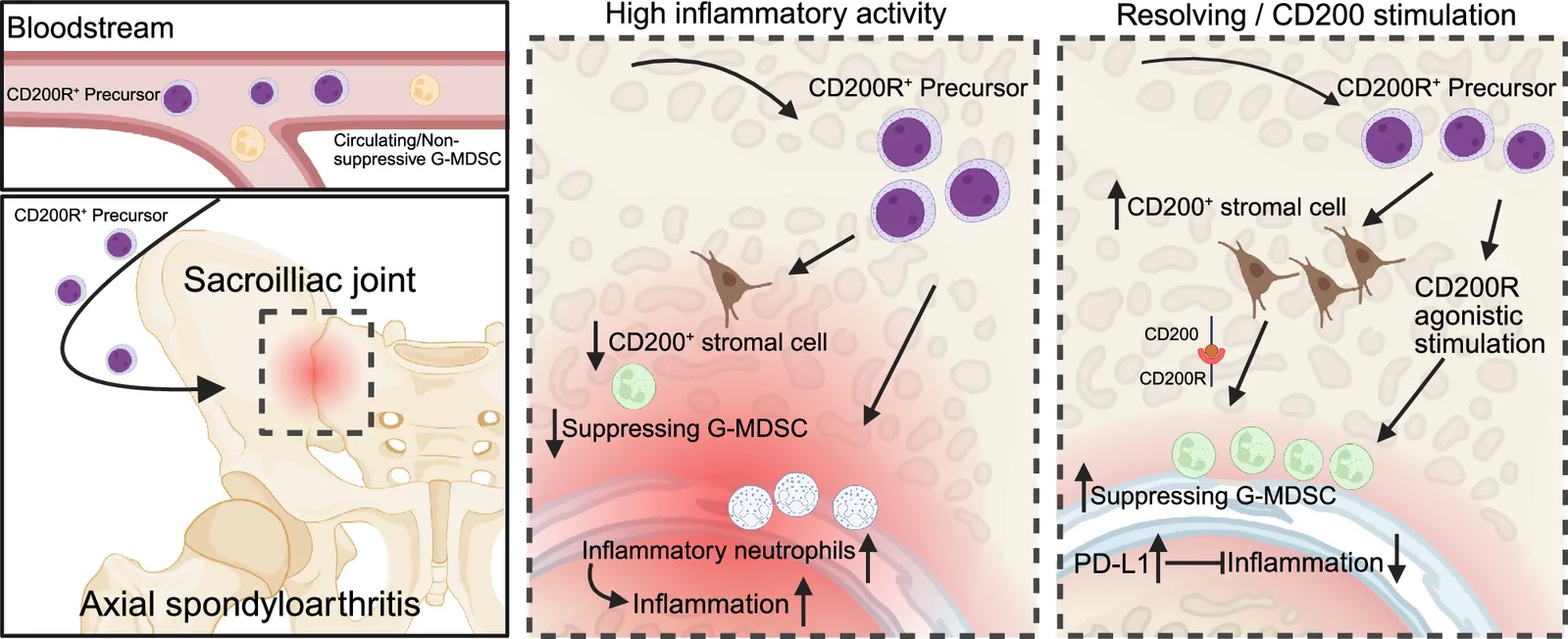
Graphical summary. Circulating CD200R1^+^ granulocytic precursors are transcriptionally immature and functionally non-suppressive. They acquire immunoregulatory capacity only upon entry into the inflamed axial joint, where their differentiation is determined by the local stromal context. Under conditions of high inflammatory activity, CD200/CD200R1 engagement is reduced, PD-L1 induction on infiltrating precursors fails and the resulting G-MDSC pool remains functionally incompetent. Inflammatory neutrophils accumulate in the infiltrate and inflammation is perpetuated, despite an expanded precursor compartment in blood and tissue. Where CD200^+^ stromal cells are available, or upon CD200R1 engagement by an agonistic antibody, the same precursors differentiate into PD-L1^+^ G-MDSCs, which suppress CD4⁺ T cell proliferation in a PD-L1-dependent manner and attenuate arthritis, whereas CD200 blockade abrogates both PD-L1 induction and therapeutic benefit. Graphical summary created in BioRender [Ramming, A. (2026) https://BioRender.com/6l4w03z].

In this study, we show that granulocytic myeloid-derived suppressor cells (G-MDSCs) gain their immunoregulatory properties in the tissue by receiving signals from a CD200^+^ resident fibroblast subset (Fig. 7). By combining in-depth human and experimental data, we reveal that G-MDSCs are not only increased in peripheral blood and tissues of arthritis patients and arthritic mice but are also functionally reprogrammed within the inflamed tissue niche. These findings highlight the role of resident cells priming G-MDSCs into their immune regulatory function, a process that is disturbed in axSpA. The increase in circulating G-MDSCs observed in axSpA likely reflects disease-specific alterations in myelopoiesis. Experimental spondyloarthritis is characterized by GM-CSF-driven dysregulation of hematopoietic stem cells, with differentiation bias toward granulocyte-monocyte progenitors and extramedullary myelopoiesis in inflamed joints^30^. Additionally, serum GM-CSF levels are elevated in axSpA patients and persist despite anti-TNF therapy^31^. We propose that this enhanced myeloid output represents a compensatory attempt to generate immunoregulatory cells. However, when these precursors encounter tissue niches with diminished CD200^+^ fibroblasts, they fail to acquire full suppressive function via CD200R1-mediated PD-L1 induction, perpetuating inflammation.

Our scRNA-seq data analysis and our experimental axSpA model provide mechanistic insight into G-MDSC ontogeny. We demonstrate that these cells arise locally from immature, *Hmgb2*⁺ neutrophil progenitors within inflamed tissues. The identification of this precursor state supports the concept of in situ differentiation, shaped by microenvironmental cues. Notably, the suppressive capacity of G-MDSCs, as measured in functional assays, was largely restricted to tissue-derived cells. This finding reinforces that local signals, rather than intrinsic programming, govern the immunoregulatory fate of G-MDSCs. A key insight was the role of stromal CD200/CD200R1 signaling in licensing G-MDSCs. Stromal fibroblasts expressing CD200 were spatially and transcriptionally positioned to interact with *Hmgb2*⁺ precursors via CD200R1. In line with this, CD200R1 stimulation in vivo enhanced the frequency and PD-L1 expression of G-MDSCs in inflamed joints without expanding the overall neutrophil pool, supporting a functional reprogramming model. This observation aligns with previous studies showing that CD200/CD200R1 signaling on granulocytes can be modulated under inflammatory conditions, including cardiac surgery^32^, highlighting the responsiveness of this axis in diverse settings.

The suppressive activity of G-MDSCs was associated with PD-L1 upregulation, a pathway classically linked to immune evasion in cancer. PD-L1⁺ MDSCs have been described in several tumor models, where they inhibit effector T cells and contribute to resistance against PD-1/PD-L1 blockade^33^. Our study extends this paradigm to immune-mediated inflammation, showing that PD-L1 expression in G-MDSCs is not limited to cancer but can be induced within inflamed stromal niches in autoimmune disease. Importantly, our findings are consistent with data from experimental autoimmune encephalomyelitis, in which PD-L1 expression was required for the suppressive function of MDSCs^34^. In direct support of this, we demonstrate that PD-L1 expression is functionally required for G-MDSC-mediated suppression, as anti-PD-L1 blockade was sufficient to abrogate their suppressive capacity in vitro. Hence, local fibroblasts modulate neutrophil fate via CD200R1 signaling, inducing a suppressive G-MDSC phenotype that expresses PD-L1 and may help counteract tissue inflammation.

Our findings clarify an important distinction between G-MDSC quantity and quality. The accumulation of G-MDSCs in axSpA tissue reflects inflammatory recruitment rather than functional capacity. In vivo evidence for this was provided by anti-CD200 blockade in hTNFtg/*Il23mc* mice, which reduced the number of PD-L1^+^ G-MDSCs in the inflamed axial joint and aggravated arthritis and by precursor transfer experiments, in which anti-CD200 blockade during differentiation impaired PD-L1⁺ G-MDSC generation from transferred precursors and abolished their therapeutic benefit. CD200R1 agonist treatment increased the proportion and intensity of PD-L1^+^ G-MDSCs without significantly altering total G-MDSC numbers, demonstrating that CD200 signaling specifically programs suppressive capacity rather than controlling recruitment. This explains the paradox of increased G-MDSC numbers in active disease: persistent inflammation drives compensatory precursor recruitment, but these cells fail to acquire full suppressive function due to diminished CD200^+^ fibroblasts in the tissue microenvironment. The therapeutic implication is that restoring functional maturation of existing G-MDSCs via CD200R1 agonism may be more effective than attempting to increase their numbers.

The selective tissue-specific emergence of these cells underscores the context dependency of neutrophil plasticity. Given their ability to suppress T cell proliferation and effector responses, G-MDSCs may serve as a natural brake on excessive inflammation. In line with this, we observed an inverse correlation between the numbers of CD200⁺ fibroblasts in the tissues and disease severity in patients with axSpA, suggesting that the local CD200/CD200R1 axis may be disrupted or insufficiently active in the tissue. This was directly confirmed in the disease model, where blocking CD200 in the axSpA model itself was sufficient to reduce PD-L1^+^ G-MDSCs in the axial joint and aggravate disease, demonstrating that an intact CD200/CD200R1 axis is required to sustain local immunoregulation in experimental axSpA. The reduced CD200⁺ fibroblast numbers observed in patients could reflect changes in the fibroblast-mediated immunoregulation under certain inflammatory conditions, with fibroblast functional phenotypes shifting from pro-resolving to pro-inflammatory states. Recent data also support the notion that fibroblast identity and function are dynamically regulated during inflammation, with subsets capable of either perpetuating or resolving inflammation depending on local cues and cytokine exposure^16^.

From a therapeutic perspective, enhancing the differentiation or activity of PD-L1⁺ G-MDSCs via CD200R1 agonists could offer a novel strategy to restore immune balance in certain forms of inflammatory diseases, such as axSpA. Our data support this approach by demonstrating that CD200R1 stimulation increases PD-L1 expression on G-MDSCs and reduces arthritis in mice in vivo. The dual observation of increased PD-L1 and functional immunosuppression aligns with prior cancer studies where targeting MDSCs improved checkpoint therapy^33^, but our work extends this concept to inflammation.

A limitation of this study is that the human data establish association rather than causality: reduced CD200^+^ fibroblast abundance with rising disease activity and the spatial association of PD-L1^+^ cells with CD200^+^ niches align with the mechanism defined in mice, but functional testing of G-MDSCs from axial tissue is constrained by the minimal material obtainable from these joints. Whether restoring this signal is therapeutically effective in axSpA is a question for interventional studies.

In conclusion, our study defines G-MDSCs as tissue-induced, PD-L1-expressing suppressor cells shaped by CD200⁺ fibroblasts. These findings highlight the broader relevance of fibroblast-myeloid interactions in regulating neutrophil plasticity and suggest that tissue-specific reprogramming of granulocytes could be therapeutically leveraged to dampen inflammation in autoimmune diseases.

## Methods

### Human subjects

Human research received approval from the local ethical committees of the University of Erlangen-Nürnberg (30_19B) and the Ruhr-Universität Bochum (18-6671-BR) with written informed consent obtained from all participants. Clinical assessments were conducted by experienced rheumatologists who were blinded to the study’s objectives. Participants diagnosed with rheumatoid arthritis (RA) (*n* = 27), psoriatic arthritis (PsA) (*n* = 29) and axial spondyloarthritis (*n* = 35) met established classification criteria (ACR, CASPAR, ASAS)^35–37^. Control subjects were non-arthritic individuals (*n* = 29). Disease activity was assessed using standardized composite scores, as detailed in the respective figure captions.

Needle biopsies of sacroiliac joints (SIJ) were obtained under sterile conditions following patient informed consent. Prior to the biopsy, MRI was performed to assess active disease, specifically identifying regions of subchondral bone marrow edema as the target area, as this compartment constitutes the site of active sacroiliitis. The biopsy was performed under CT guidance by experienced interventional radiologists, targeting the same anatomical site identified by MRI. In line with the biopsy technique described previously^38^, tissue samples comprised joint cartilage and the underlying subchondral bone at the site of bone marrow edema. Local anesthesia was applied and needle placement was confirmed by CT imaging prior to sample collection.

Biopsies of vertebral facet joints were taken under sterile conditions upon patient’s informed consent during spinal surgery. The region of interest had been verified prior by MRI as inflamed for axSpA patients and as disease-free for the controls.

### Mice

hTNFtg (*B6.Cg-Tg(TNF)197Gkl*) mice, as described in ref. ^39^, were maintained on a *C57BL/6N* background. Control animals were littermates negative for hTNFtg. *C57BL/6Rj* mice were purchased commercially (Janvier Labs). The mice were housed in standard type II long cages with a 12-h day-night cycle, provided with standard diet (V1534-000, ssniff Spezialdiäten) and had ad libitum access to water. The environmental conditions were controlled with a temperature range of 20–24 °C, relative humidity of 45% to 65% and an air exchange rate of 15-fold to 20-fold. Mice were housed in groups of three to five per cage. For externally sourced test animals, a 2-week acclimation period was observed upon delivery. At the start of the experiment, all animals were between 8 and 10 weeks old. For experimental approaches mice were stratified evenly by sex and then randomized to the different groups. The experimental procedures were conducted in accordance with local regulations and approved by the Government of Unterfranken, Würzburg (protocols 55.2-2532-2-1438, 55.2-2532-2-1886, 55.2-2532-2-1592, 55.2-2532-2-1061 and 55.2-2532-2-2337).

### Animal models of arthritis

Symptoms of arthritis of hTNFtg mice were assessed over a period of 6–10 days starting from onset of arthritis at 8–9 weeks of age. Additionally, *Il23mc* treatment was administered on the first day of symptom assessment for hTNFtg mice and their respective control littermates. The *Il23mc* vector, comprising the IL-12b (IL-12p40) and IL-23a (IL-23p19) subunits connected by a flexible region, was under the control of the albumin promoter to ensure efficient expression in hepatocytes. This vector, provided by S. Wirtz (Department of Internal Medicine 1, Erlangen, Germany), was produced in *Escherichia coli* DH5α grown in terrific broth medium without removal of the vector backbone. Plasmids were purified using the PureLink HiPure Plasmid Maxiprep Kit (Cat# K210007, Thermo Fisher), followed by endotoxin removal using the MiraCLEAN Endotoxin Removal Kit (Cat# MIR 5900, Mirus) to ensure efficient elimination of endotoxins. Subsequently, 3 μg of naked plasmid DNA in Ringer’s solution, equivalent to 10% of body weight in volume, was administered via hydrodynamic gene transfer into the tail vein^40,41^. Joint swelling was assessed at the ankles by measurement with calipers (Kroeplin) and calculated as the average change from baseline at day 0 of the experiment.

Therapeutic antibody treatment was started 1 day before hydrodynamic gene transfer. Animals received CD200R1 agonist (Cat# 123917, OX-110, BioLegend, 100 μg/mouse), anti-CD200 blocking antibody (Cat# BE0299, OX-90, BioXCell, 100 μg/mouse) or rat IgG2a isotype control (Cat# 400544, RTK2758, BioLegend, 100 μg/mouse) intraperitoneally on day −1, 3, 5 and 7 and mice were sacrificed on day 10.

Serum transfer arthritis was induced in recipient mice by intraperitoneal injection of 200 μL arthritogenic serum from K/BxN mice (provided by K. Sarter-Zaiss, University Hospital Erlangen)^42^.

### Adoptive transfer of G-MDSCs or precursor cells

For adoptive transfer of mature G-MDSCs, bone marrow cells were harvested from 8-week-old *C57BL/6Rj* mice by flushing femora and tibiae, followed by centrifugation and red blood cell lysis by applying RBC lysis buffer (Cat# 422401, BioLegend). Ly6G⁺ cells were isolated using magnetic microbeads (Cat# 130-120-337, Miltenyi Biotec). Purified cells were cultured for 2 days in RPMI 1640 supplemented with 10% fetal calf serum (Cat# 10270106, Gibco), 2 mM L-glutamine (Cat# G7513, Gibco), penicillin/streptomycin (Cat# 11548876, Gibco), 10 ng/mL GM-CSF (Cat# 415-ML-010/CF, R&D Systems) and 10 μg/mL CD200R1 agonist (Cat# 123917, BioLegend, OX-110). PD-L1 expression was confirmed by flow cytometry. In parallel, serum transfer arthritis was induced in recipient mice as described above. 1.5 × 10^6^ cells in 150 μl 0.1%BSA/PBS were adoptively transferred intravenously into recipient mice on days 0, 5 and 7 of serum transfer arthritis. Mice were sacrificed on day 9 for analysis at peak of inflammation.

To test the in vivo differentiation by adoptive transfer of precursor cells, bone marrow cells were harvested from 8-week-old *C57BL/6Rj* mice by flushing femora and tibiae, followed by centrifugation and red blood cell lysis by applying RBC lysis buffer (Cat# 422401, BioLegend). Precursor cells were isolated by FACS sorting viable Ly6G^+^CD177^+^SiglecF^−^CD11c^−^CD3^−^B220/CD45R^−^ and labeled with CellTrace CFSE Cell Proliferation Kit (Cat# C34554, Invitrogen) according to manufacturer’s instructions. 3 × 10^6^ cells in 300 μl RPMI were adoptively transferred intravenously into recipient mice on days 2 and 7 of serum transfer arthritis. Arthritic mice receiving 300 μl RPMI intravenously served as adoptive transfer controls. In parallel, recipient and control mice undergoing serum transfer arthritis received either anti-CD200 blocking antibody (Cat# BE0299, OX-90, BioXCell, 100 μg/mouse) or rat IgG2a isotype control (Cat# 400544, RTK2758, BioLegend, 100 μg/mouse) intraperitoneally on days 0 and 5 relative to serum transfer. Mice were sacrificed on day 9 for analysis at peak of inflammation.

### Magnetic resonance imaging of mice

MRI measurements were performed on a preclinical 7 T MRI scanner (ClinScan 70/30, Bruker). For in vivo imaging, animals were anesthetized using 3% isoflurane and maintained at 1.5% isoflurane. The respiration was monitored by breath sensors and kept constant throughout the entire imaging procedure while body temperature was stabilized using a heating circulator bath (Thermo Fisher Scientific). An axial T1-weighted spin echo sequence was acquired two times before and after the administration of contrast agent Gadolinium. The sequence parameters were: field-of-view = 29 × 29 mm^2^, voxel size = 0.067 × 0.067 × 0.7 mm^3^, echo time = 9 ms, repetition time = 600 ms, band width = 205 Hz px^−1^, acquisition time = 8:35 min. After the administration of the contrast agent, an axial T2-weighted turbo spin echo sequence was acquired with a field-of-view of 30 × 30 mm^2^, voxel size = 0.117 × 0.117 × 0.7 mm^3^, echo time = 53 ms, repetition time = 3800 ms, band width = 130 Hz px^−1^, acquisition time = 4:37 min. The volume of inflammation was evaluated on T1-weighted images after subtraction of pre- from post-gadolinium was performed and the volume of contrast-enhancing areas was quantified by manual segmentation (3D Slicer, version 5.6.1^43^).

### Histologic analysis

Murine ankle joints, intervertebral discs, biopsies from human SIJ and spine were fixed in PBS containing 4% (wt/vol) formaldehyde for 16–24 h at 4 °C. Tissue was transferred into decalcification buffer containing 0.5 M EDTA at pH 7.4 for 3–5 weeks and changed twice per week. Tissue was then dehydrated and embedded in paraffin blocks, cut to 3–5 μm thick sections and mounted on slides. For cryosectioning, selected murine intervertebral discs were fixed in 4% (wt/vol) paraformaldehyde overnight at 4 °C, decalcified in 0.5 M EDTA (pH 7.4) for 3 days, cryoprotected in cryoprotection solution overnight (PBS containing 20% (wt/vol) sucrose and 2% (wt/vol) polyvinylpyrrolidone), embedded in Tissue-Tek OCT compound (Cat# 4583, Sakura) and sectioned at 20 μm using a cryotome (Leica). For Hematoxylin & Eosin staining, sections were deparaffinized by heating the slides at 65 °C for 30 min and subsequent washing in Histo Clear (Cat# HS-200-1GAL, National Diagnostics) followed by rehydration with a series of 100% (vol/vol) ethanol, 95% (vol/vol) ethanol, 80% (vol/vol) ethanol, 60% (vol/vol) ethanol and water. Mayer’s Hematoxylin solution (Cat# HT1079-1SET, Merck) was applied for 10 min. Hematoxylin was blued by rinsing the slides for 10 min in tap water. Eosin counter stain (0.3% (wt/vol) Eosin Y (Cat# HT110216-500ML, Sigma-Aldrich), 0.01% (vol/vol) acetic acid) was applied for 3 min and washed off with deionized water. Images were acquired on a NanoZoomer S60 (Hamamatsu, 2.7.39) or Aperio Versa 8 Slide Scanner (Leica Biosystems, 1.0.4). A periarthritis or spondylitis score was established to assess the inflammatory infiltrate in the respective joints: grade 0 (no infiltrate), 1 (mild infiltration), 2 (moderate infiltration) and 3 (severe infiltration). Scores were assessed blinded by a minimum of two researchers, respectively.

### Tissue dissociation

Mice were euthanized by cervical dislocation under anesthesia and ankle joints or spleen were carefully removed. To generate single cell suspensions from ankles, legs with intact bones were dissected. After tibiae were removed, tendons as well as joint capsules were cut to enable optimal entrance of digestive solution into tissue. Tissue was digested in RPMI 1640 medium (Cat# 11875093, Gibco) supplemented with 2 mg/mL collagenase D (Cat# 11088866001, Roche), 0.2 mg/mL Dispase II (Cat# D4693-1G, Sigma-Aldrich) and 0.1 mg/mL DNase I (Cat# 10104159001, Roche). Ankle joints were incubated three times for 30 min at 37 °C on a ThermoShaker (Eppendorf) at 2000 rpm. After each step, the supernatant was collected and filtered through a 100 μm filter and washed in a larger volume of RPMI 1640 supplemented with 10 mM EDTA (Cat# 51201, Lonza) and 10% FCS (Cat# 10270106, Gibco). Red blood cells (RBC) were lysed after digestion by applying RBC lysis buffer (Cat# 422401, BioLegend). Subsequently, debris was removed by Debris Removal Solution (Cat# 130-109-398, Miltenyi Biotec) according to manufacturer’s instructions. Cells were then washed in PBS supplemented with 5 mM EDTA and 2% FCS and filtered through 40-μm cell strainers. Cell counts were obtained by Trypan blue staining using a TC20 automated cell counter (Bio-Rad).

Mouse spleen cells were prepared by mincing the spleen and transferring to a 70 μm filter. The tissue was then passed through the filter and rinsed with PBS. After RBCs were lysed, cells were filtered through a 40 μm filter and resuspended in PBS supplemented with 5 mM EDTA and 2% FCS and counted as described above.

### Flow cytometry and cell sorting

Single cells from murine ankle joints and spleen were stained for 30 min on ice in PBS supplemented with 5 mM EDTA and 2% FCS.

For the assessment of circulating G-MDSCs and granulocytes in human blood venous human blood was collected with a standard collection system and EDTA as anticoagulant (Sarstedt). The collected blood was directly incubated with fluorochrome labeled antibodies. Following incubation with antibodies for 20 min on ice, red blood cells were lysed and nucleated cells were fixed using RBC Lysis and Fixation Solution (Cat# 422401, BioLegend) according to manufacturer’s instructions.

Fc receptor blocking (Cat# 101302, BioLegend) was done prior to staining with fluorophore-labeled antibodies. Dead cells were excluded using Zombie Violet (1:500, Cat# 423113, BioLegend), Fixable Viability Stain 510 (1:1000, Cat# 564406, BD Biosciences) or Fixable Viability Dye eFluor 780 (1:4000, Cat# 65-0865-14, Thermo Fisher).

The following antibodies were used to detect G-MDSCs in murine spleen and ankle joints: anti-CD45 (30-F11, 1:1000, Cat# 109822), anti-CD3ε (145-2C11, 1:100, Cat# 100306), anti-CD45R/B220 (RA3-6B2, 1:500, Cat# 103206), anti-CD49b (DX5, 1:100, Cat# 108906), anti-CD11c (N418, 1:100, Cat# 117306), anti-CD170/SiglecF (S17007L, 1:50, Cat# 155504), anti-Ly6G (1A8, 1:100, Cat# 127641), anti-CD11b (M1/70, 1:1,000, Cat# 101212), anti-Ly6C (HK1.4, 1:100, Cat# 128033), anti-CD182/CXCR2 (SA044G4, 1:100, Cat# 149304), anti-CD101 (Moushi101, 1:100, Cat# 25-1011-82, Thermo Fisher), anti-CD274/PD-L1 (10F.9G2, 1:100, Cat# 124308). Antibodies to detect granulocytic cells in human blood were: anti-CD45 (HI30, 1:250, Cat# 304036), anti-CD66b (G10F5, 1:1000, Cat# 305104), anti-CD15 (W6D3, 1:1500, Cat# 323030). Antibodies to detect G-MDSCs from human blood were: anti-CD45 (5B1, 1:500, Cat# 130-096-906, Miltenyi Biotec), anti-CD33 (AC104.3E3, 1:50, Cat# 130-113-345, Miltenyi Biotec), anti-HLA-DR (L243, 1:400, Cat# 307606), anti-CD14 (M5E2, 1:50, Cat# 301820), anti-CD15 (W6D3, 1:50, Cat# 323030), anti-CD11b (ICRF44, 1:50, Cat# 301315), anti-CD3 (UCHT1, 1:200, Cat# 300406), anti-CD19 (HIB19, 1:200, Cat# 302206), anti-CD56 (HCD56, 1:200, Cat# 318304), anti-CD16 (3G8, 1:200, Cat# 302006). Antibodies to detect CD200R1 expression in murine neutrophils were: anti-CD170/SiglecF (S17007L, 1:50, Cat# 155504), anti-CD25 (3C7, 1:250, Cat# 101904), anti-CD90.2 (30-H12, 1:500, Cat# 105338), anti-CD4 (GK1.5, 1:1500, Cat# 100422), anti-CD200R1 (OX-110, 1:200, Cat# 566345, BD Biosciences), anti-CD3ε (145-2C11, 1:100, Cat# 100341), anti-CD11b (M1/70, 1:1000, Cat# 101263). Antibodies to sort precursor cells from murine bone marrow were: anti-Ly6G (1A8, 1:500, Cat# 127628), anti-CD177 (Y127, 1:100, Cat# 566599, BD Biosciences), anti-CD3 (17A2, 1:100, Cat# 100222), anti-CD45R/B220 (RA3-6B2, 1:500, Cat# 103224), anti-CD11c (N418, 1:100, Cat# 117324), anti-CD170/SiglecF (S17007L, 1:100, Cat# 155532). Antibodies to detect PD-L1 expression in CFSE labeled cells in murine ankle joints were: anti-Ly6G (1A8, 1:500, Cat# 127628), anti-CD274/PD-L1 (10F.9G2, 1:100, Cat# 124308), anti-CD45 (30-F11, 1:2000, Cat# 103115), anti-F4/80 (BM8, 1:100, Cat# 123113). The following antibodies were used to detect in vitro generated PD-L1^+^ G-MDSCs: anti-CD11b (M1/70, 1:500, Cat# 101212), anti-Ly6G (1A8, 1:100, Cat# 127606), anti-PD-L1 (10F.9G2, 1:100, Cat# 124308). The following antibodies were used to detect T cells after co-culture with PD-L1^+^ G-MDSCs or PD-L1^−^ granulocytes: anti-CD4 (RM4.5, 1:1000, Cat# 100526), anti-TCR-β (H57-597, 1:500, Cat# 109212). Antibodies were purchased from BioLegend if not indicated otherwise. Samples were acquired using a Beckman Coulter Gallios (Human G-MDSC and murine CD200R1 neutrophils, Gallios Software v1.2), a Beckman Coulter Cytoflex S (Murine G-MDSC and T cell suppression assays, CytExpert v2.2), a Cytek Aurora (Human Granulocytes, SpectroFlo 3.1.0) or Cytek Northern Lights (PD-L1^+^ G-MDSCs, SpectroFlo 3.1.0) and analysed using FlowJo v.10.10 or Kaluza 2.1. Unmixing reference controls for Cytek Aurora were generated utilizing primary human peripheral blood mononuclear cells and single stainings of the corresponding antibodies. For unmixing of fluorescent signal at Cytek Northern Lights single stainings of cells were performed. Cell sorting was performed using a Beckman Coulter MoFlo Astrios EQ (running with Beckman Coulter Astrios Summit v6.3.1). Sorted populations were re-analysed to determine target cell purity after sorting (>98% purity).

### T cell suppression assay

To test the suppressive capacity of murine G-MDSCs, cells were FACS sorted from the ankle joints and spleens 7 days after *Il23mc* induction in hTNFtg mice, while naïve CD4^+ ^T cells were isolated from spleen with Naïve CD4^+^ mouse T cell isolation kit (Cat# 130-104-453, Miltenyi Biotec) according to manufacturer’s instructions. 1 × 10^7^ cells/mL T cells were labeled in 1 μM CFSE for 6 min at 37 °C. Labeling was stopped by adding 2 volumes of FCS and washing cells twice in PBS supplemented with 5 mM EDTA and 2% FCS. 1 × 10^5^ of labeled naïve CD4^+ ^T cells were then plated together with G-MDSC subsets in a 1:2 or 1:10 ratio in U-shaped 96-well plates coated with CD3ε (145-2C11, Cat# 100359, BioLegend, 5 μg/mL) and CD28 (37.51, Cat# 102121, BioLegend, 3 μg/mL) for 2 h at 37 °C. The culture medium was RPMI supplemented with 10% FCS, 100 U/mL penicillin, 100 μg/mL streptomycin, 2 mM L-glutamine, 2.5 μg/mL amphotericin B and 50 ng/mL recombinant IL-2 (Cat# 575406, BioLegend). Labeled naïve CD4^+ ^T cells alone with CD3/CD28/IL-2 stimulus and labeled naïve CD4^+ ^T cells without any activation were used as positive and negative controls, respectively. Cells were incubated for 3 days followed by FACS acquisition at Beckman Coulter Cytoflex S.

To test whether T cell suppression is PD-L1-dependent we sorted Ly6G⁺ cells from the bone marrow using the Ly6G⁺ microbeads isolation kit (Cat# 130-120-337, Miltenyi Biotec) according to manufacturer’s instructions and cultured with either GM-CSF (Cat# 576304, BioLegend, 10 ng/mL), G-CSF (Cat# 574604, BioLegend, 10 ng/mL) or GM-CSF with anti-PD-L1 antibody (10F.9G2, Cat# BE0101, BioXCell, 10 μg/mL) for 48 h in RPMI 1640 supplemented with 10% fetal calf serum, 2 mM L-glutamine, penicillin/streptomycin. Naïve CD4^+ ^T cells were isolated from spleen with Naïve CD4^+^ T Cell Isolation Kit (Miltenyi Biotec) according to manufacturer’s instructions. 1 × 10^6^ cells/mL T cells were labeled with 0.5 μM CFSE for 5 min at room temperature. Labeling was stopped by adding five times the original staining volume of culture medium to the cells and incubating for 5 min. 1 × 10^5^ of labeled naïve CD4^+ ^T cells were then plated together with Ly6G⁺ cells after 48 h pre-treatment in a 1:2 ratio in U-shaped 96-well plates. Activation of T cells was achieved by pre-coating the wells with antibody against CD3ε (BioLegend, 145-2C11, 1 μg/mL) for 2 h at 37 °C. The culture medium was Advanced RPMI 1640 (Gibco) supplemented with 10% FCS, 100 U/mL penicillin, 100 μg/mL streptomycin, 2 mM L-glutamine, 2.5 μg/mL amphotericin B, 10 mM HEPES, 0.05 mM β-mercaptoethanol and recombinant mouse IL-2 (BioLegend, 5 ng/mL). Soluble anti-CD28 antibody (BioLegend, 37.51) was added to the culture medium at a final concentration of 5 μg/mL. Labeled naïve CD4^+ ^T cells alone with CD3/CD28/IL-2 stimulus and labeled naïve CD4^+ ^T cells without any activation served as positive and negative controls, respectively. Cells were incubated for 3 days followed by FACS acquisition at Beckman Coulter Cytoflex S. T cell suppression (%) was calculated per mouse as (MFI_sample_/MFI_activated control_ −1) × 100, where MFI denotes the geometric mean fluorescence intensity of CFSE in CD4^+^ T cells. Higher values indicate stronger suppression.

### Fluorescence imaging

Deparaffinization, rehydration and epitope retrieval of paraffin-embedded sections from human SIJ, spine and murine intervertebral discs was performed as described above. Blocking step was performed in PBS supplemented with 2% BSA (wt/vol) and 5% donkey serum (vol/vol) for 1 h at room temperature. The primary antibody mix was applied on the sections and incubated in a hybridization chamber for 16 h at 4 °C. Secondary antibodies were stained for 1 h at room temperature followed by incubation with DAPI nuclear staining (1:1000, Cat# sc-3598, Santa Cruz). The following antibodies were used in human tissues: anti-CD33 (Polyclonal, 1:100, Cat# bs-2042R, Bioss), anti-HLA-DR (LN3, 1:100, Cat# LS-B8114, LSBio), anti-CD20 (H1, 1:500, Cat# 555677, BD Biosciences), anti-CD56 (123A8, 1:1000, Cat# NBP2-44713, Novus), anti-CD11c (Polyclonal, 1:300, Cat# orb13554, Biorbyt), anti-CD3 (PS1, 1:50, Cat# ab699, Abcam), anti-CD14 (4B4F12, 1:100, Cat# ab182032, Abcam), anti-CD16 (2Q1240, 1:50, Cat# sc-70548, Santa Cruz), anti-CD123 (6H6, 1:100, Cat# 306002, BioLegend), anti-CD274/PD-L1 (E1L3N, 1:100, Cat# 85164SF, Cell Signaling), anti-CD200 (Polyclonal, 1:100, Cat# AF2724, R&D Systems), anti-Podoplanin (Polyclonal, 1:100, Cat# AF3670, R&D Systems). The following antibodies were used in murine intervertebral discs: anti-CD182/CXCR2 (Polyclonal, 1:200, Cat# PA5-100951, Invitrogen), anti-Ly6G (1A8, 1:100, Cat# 127602, BioLegend). Following fluorochrome-labeled secondary antibodies were used: Donkey anti-rabbit AlexaFluor 488 (Polyclonal, 1:200, Cat# A21206, Thermo Fisher Scientific), Donkey anti-rabbit AlexaFluor Plus 555 (Polyclonal, 1:500, Cat# A32794, Thermo Fisher Scientific), Donkey anti-rabbit AlexaFluor 555 (Polyclonal, 1:200, Cat# ab150062, Abcam), Donkey anti-sheep AlexaFluor 555 (Polyclonal, 1:200, Cat# A21436, Thermo Fisher Scientific), Donkey anti-rat AlexaFluor Plus 647 (Polyclonal, 1:500, Cat# A48272, Thermo Fisher Scientific), Donkey anti-mouse AlexaFluor 488 (Polyclonal, 1:200, Cat# ab150105, Abcam) and Donkey anti-Goat AlexaFluor 647 (Polyclonal, 1:200, Cat# A-21447, Thermo Fisher Scientific).

Cryosections were washed in PBS and blocked for 1 h at room temperature in PBS supplemented with 2% BSA (wt/vol), 5% donkey serum (vol/vol) and 0.3% Triton X-100 (vol/vol). Primary antibodies were diluted in blocking buffer and applied overnight at 4 °C. Sections were washed three times with PBS, incubated with DAPI nuclear stain (1:1000, Santa Cruz), washed again in PBS and mounted using fluorescence mounting medium. The following directly conjugated antibodies were used for murine cryosections: anti-PD-L1 Alexa Fluor 647 (10F.9G2, 1:25, Cat# 568306, BD Biosciences) and anti-Ly6G Alexa Fluor 594 (1A8, 1:100, Cat# 127636, BioLegend).

Stained sections were imaged on a Leica Thunder DMi8 microscope (Leica Microsystems) using z-stacks processed via maximum intensity projection. Data were exported as TIFFs via Leica Application Suite X (3.7.4.23463). For G-MDSC localization, human SIJ/spine and murine intervertebral disc sections were analysed in ROIs (0.5–1.5 mm²) with manual counts. The ROIs in human tissue analysis were selected randomly and in a blinded manner, focusing on areas of cellular infiltration within the subchondral bone marrow. For niche analysis, CD200^+^ and PD-L1^+^ cells were quantified using Fiji’s Thresholding (Moments method). PD-L1^+^ cells within a 100 μm radius^44^ of CD200^+^ centroids were classified as niche-associated via a custom distance-calculation script. CD200^+^ cell density was derived by normalizing CD200^+^ counts to DAPI^+^ nuclei, the latter auto-detected using Fiji’s Analyze Particles (size: 2–200 px²) after Subtract Background, Gaussian Blur (σ = 0.5) and Watershed segmentation. Whole-tissue analysis focused on SIJ regions, excluding artifacts. All tools were scripted to ensure reproducibility.

### Preprocessing and analysis of neutrophil single-cell RNA sequencing datasets

Count matrices and metadata of cross tissue and cross model mice neutrophils were acquired from GSE165276^17^ and imported into R (4.3.2) as a Seurat (5.1.0)^45^ object. Cells with low quality were identified by visualizing the distributions and determining appropriate thresholds: number of UMIs <1200, number of genes <700 and mitochondrial gene ratio >0.015. Integration across sequencing libraries was performed using scVI (1.1.1)^46^ and Scanpy (1.9.8)^47^ in Python (3.9.18), following the standard workflow. Briefly, genes expressed in fewer than 5 cells were discarded. Counts were normalized using “pp.normalize_total”, scaled using “pp.log1p” and the top 2000 highly variable genes were selected. The scVI model was initialized with library as the categorical batch variable and cell cycle score and ribosomal gene ratio as continuous covariates. The model was run with 2 layers and a 30-dimensional latent space on the count layer. Clustering was performed using the Leiden algorithm^48^ from Scanpy with a resolution of 0.7. The resulting clustering and scVI embeddings were imported back into R and added to the Seurat object for downstream analysis. Cluster markers were identified using “FindAllMarkers” and clusters were annotated based on the top-expressing genes. Subclustering of the G-MDSCs was performed in Seurat by first regenerating the neighborhood graph based on the scVI embeddings, then using “FindNeighbors” followed by “FindSubCluster” with a resolution set to 0.3.

### Early neutrophil score

The early neutrophil score was calculated using UCell (v2.6.2)^49^ by assigning genes relevant to the early neutrophil signature with a “+” sign and genes related to the late neutrophil signature with a “−” sign. This gene list was obtained from a previous publication^17^. The exact genes used for the early and late signatures are provided in Table S2. To compare this signature among neutrophil subsets, the UCell score was summarized (median) for all cells within each cluster and each replicate and visualized as a boxplot. Statistical significance was first assessed using a Kruskal–Wallis test (*P* = 0.0014), followed by a one-vs-rest Wilcoxon rank-sum test with multiple testing correction using the Benjamini-Hochberg (BH) method.

### Gene ontology enrichment analysis

Gene ontology enrichment analysis was performed by first identifying markers for all neutrophil subsets using Seurat’s “FindAllMarkers”. The top 50 genes (ranked by adjusted *P* value and positive log2 fold change) were selected for testing using clusterProfiler’s (4.10.0)^50^ “compareCluster” function with “enrichGO” as the enrichment method.

### Neutrophil trajectory estimation

The trajectory of neutrophil differentiation in the neutrophil scRNA-seq dataset was inferred using Slingshot (2.10.0)^22^ on the principal component analysis (PCA) embeddings, with the “start.clus” parameter set to the *Hmgb2*^+^ precursor cluster. Lineages were visualized using “dittoDimPlot” from dittoSeq (1.14.2)^51^ in PCA space.

Potential of heat-diffusion for affinity-based trajectory embedding (PHATE) dimension reduction was generated using Harmony embeddings from the whole-tissue single-cell RNA-sequencing dataset derived from inflamed ankle joints of arthritic mice (serum transfer arthritis) with phateR (v1.0.7)^52^. Developmental potential along the neutrophil trajectory was further assessed using CytoTRACE (v0.3.3)^24^ and CytoTRACE 2 (v1.1.0)^25^ with default parameters.

### Preprocessing and analysis of whole tissue single-cell RNA sequencing datasets

Single-cell RNA-seq data from whole tissue of healthy and KBxN-induced arthritis mouse models were acquired from GSE253974^23^. Data were imported into R and subjected to hashtag oligo (HTO) demultiplexing using Seurat. The HTO assay was normalized using “NormalizeData” with normalization.method = “CLR”. Sample separation was performed using “HTODemux” with the positive quantile set to 0.99. Quality control on singlets was performed and cells with <400 UMIs, <300 detected genes, mitochondrial gene ratio >0.15, or complexity (log10(genes/UMI)) <0.8 were discarded. Genes expressed in fewer than 10 cells were removed. Integration across sequencing libraries (two sequencing libraries) was performed using Harmony (0.1.0)^53^. Clustering was done in Seurat with the resolution set to 2 and annotation was done using known marker genes. Annotation of neutrophil and fibroblast subclusters was done using Seurat label transfer from our previously annotated neutrophil scRNA-seq dataset (see above) and from a previously published synovial fibroblast dataset (GSE228980)^16^. To achieve this, an additional reciprocal PCA (RPCA) integration was performed on the reference datasets, followed by “FindTransferAnchors”, “TransferData” and “AddMetaData” with default parameters on the subsetted Seurat objects containing neutrophils or fibroblasts.

### Ligand-receptor interaction analysis

Interaction analysis using the CellChat (2.1.2)^54^ ligand-receptor framework was implemented on the entire KBxN model scRNA-seq dataset using default parameters separately for each replicate. Briefly, the CellChat database was subset to “Secreted Signaling” and “Cell-Cell Contact”, followed by “identifyOverExpressedGenes”, “identifyOverExpressedInteractions”, “projectData”, “computeCommunProb”, “filterCommunication”, “computeCommunProbPathway”, “aggregateNet” and “netAnalysis_computeCentrality”.

Median interaction probability (across all replicates) from or to neutrophils and all other major cell types or fibroblast subsets, was visualized using the “netVisual_circle”. These values were also visualized using boxplots and a one-vs-rest Wilcoxon rank-sum test followed by BH correction was used to calculate *P* values.

### Statistical analysis

Statistical analysis was done with GraphPad Prism 10. Data are shown as Mean ± SD, unless otherwise indicated in the figure legends. For comparison of two groups two-sided Mann-Whitney *U*-test for nonparametric data was performed, if not otherwise stated. When two groups of samples were compared for iterating parameters or more than two groups of samples were compared, a one-way ANOVA was used, if not otherwise stated. Tukey’s range test was used as post hoc analysis of ANOVA. All statistical tests were two-tailed, unless otherwise stated in the figure legends. Simple linear regression was used to assess relationships between continuous variables, unless otherwise stated. No statistical methods were used to pre-specify sample sizes, but our sample sizes are similar to those reported in previous publications.

Investigators were not blinded to group allocation during treatment administration, as German animal welfare regulations require documentation of individual severity grading per animal by the treating investigator. All other data collection and analyses were performed blinded by at least two independent researchers. Differences were considered significant when *P* < 0.05.

### Reporting summary

Further information on research design is available in the Nature Portfolio Reporting Summary linked to this article.

## Supplementary information

Supplementary Information

Transparent Peer Review file

Reporting Summary

## Source data

Source data File

## Data Availability

No new sequencing datasets were generated in this study. Previously published datasets analysed are cited in the Methods, with their accession codes and references. Source data are provided with this paper.
