# Supplementary material for: CD200+ fibroblasts prime the immunoregulatory function of granulocytic myeloid-derived suppressor cells in inflammatory arthritis
Source: Nat Commun. 2026 Oct 13;17:10746. doi: 10.1038/s41467-026-78351-w (PMC13661617; doi:10.1038/s41467-026-78351-w)
Supplement: Supplementary file 1 [file 41467_2026_78351_MOESM1_ESM.pdf]

CD200⁺ fibroblasts prime the immunoregulatory function of granulocytic myeloid-derived suppressor cells in inflammatory arthritis

Supplementary figures and tables

Mario R. Angeli, Christian Schütz, Hashem Schmidt, Richard Demmler, Jiyang Chang, Hannah Labinsky, Maria Gabriella Raimondo, Brikena Lalazi, Katerina Stergioula, Kaiyue Huang, Vladyslav Fedorchenko, Mohan Li, Zoltán Winter, Styliani Tsiami, Arnd Lienert, Sebastian Rütten, Stefanie Weber, Markus Luber, Tobias Bäuerle, Reza Bahrami, Heinrich Boehm, Georg Schett, Simon Rauber, Xenofon Baraliakos[#] & Andreas Ramming[#]

[#] Equally contributed

Corresponding author: Andreas Ramming, Department of Internal Medicine 3, Rheumatology and Immunology, Friedrich-Alexander-University (FAU) Erlangen-Nürnberg and Universitätsklinikum Erlangen, Ulmenweg 18, 91054 Erlangen, Germany. Phone: +49-9131-85-39109. E-mail address: andreas.ramming@uk-erlangen.de

Table of contents

CD200⁺ fibroblasts prime the immunoregulatory function of granulocytic myeloid-derived suppressor cells in inflammatory arthritis 1

Supplementary figures and tables 1

- Supplementary Figure 1. Flow cytometric gating and quantification of granulocytes and MDSC subsets in arthritis patients. 2
- Supplementary Figure 2. Gating strategy and functional assessment of murine G-MDSCs in hTNFtg/*Il23mc* mice. 5
- Supplementary Figure 3. Single-cell analysis of neutrophil and fibroblast subsets in inflamed mouse joints. 7
- Supplementary Figure 4. Subclustering identifies a *Cd274*⁺ G-MDSC subpopulation in mice. 8
- Supplementary Figure 5. Adoptive transfer of *in vitro* polarized G-MDSCs and CD177⁺Ly6G⁺ precursors in serum transfer arthritis. 9
- Supplementary Table S1: Patient characteristics for sacroiliac joint CD200/PD-L1 analysis, related to Figure 4B-C and 6F 11
- Supplementary Table S2: Selected genes for early Neutrotime signature [7] 11
- Supplementary References 12

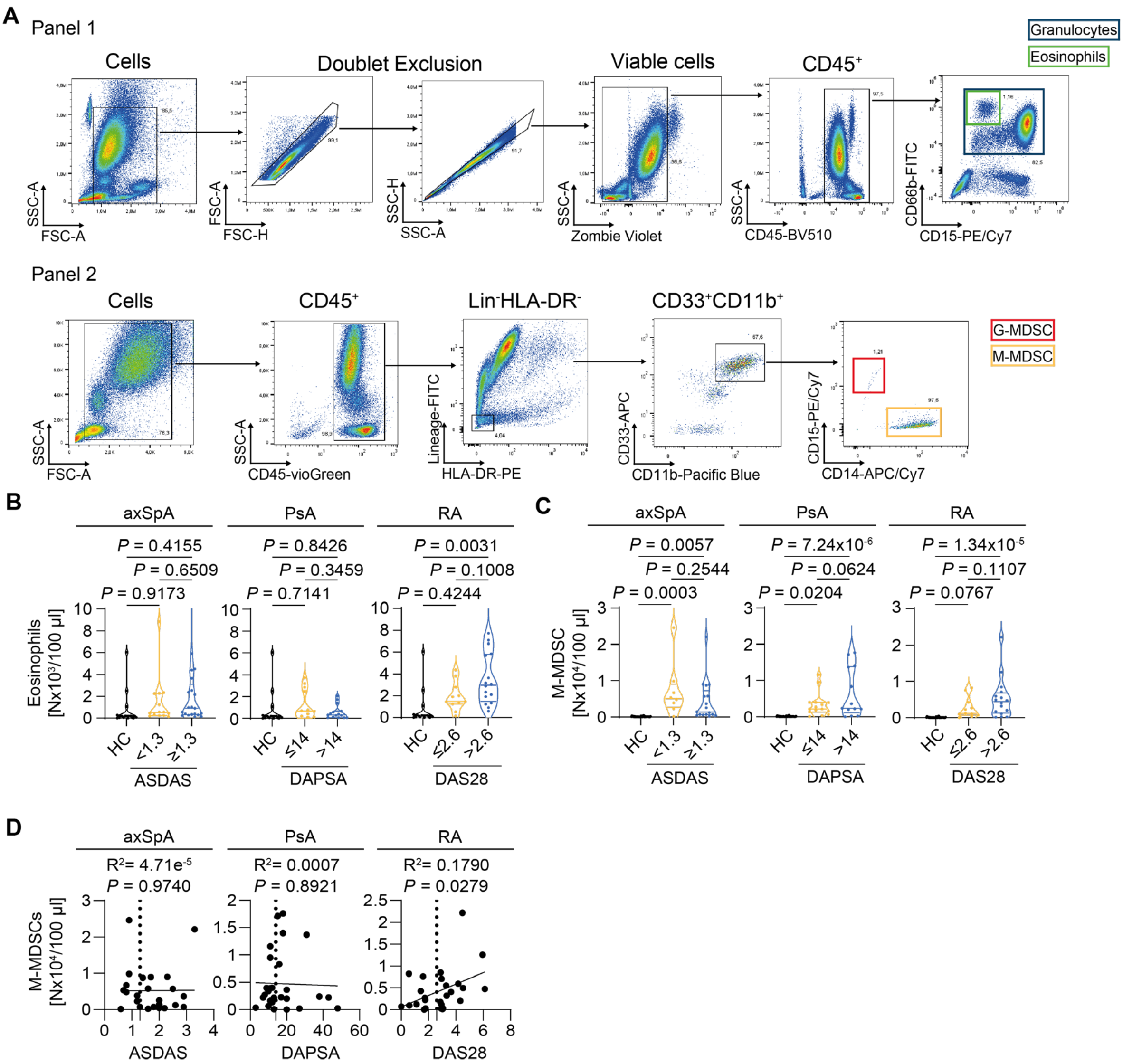

Supplementary Figure 1. Flow cytometric gating and quantification of granulocytes and MDSC subsets in arthritis patients.

(**A**) Representative gating strategy to detect total granulocytes, eosinophils (panel 1) and monocytic myeloid-derived suppressor cells (M-MDSCs) as well as granulocytic myeloid-derived suppressor cells (G-MDSCs, panel 2) from peripheral blood. Lineage cocktail in panel 2 consists of CD3, CD14, CD16, CD19, CD56. This gating strategy applies to the data shown in Figure 1B-D and Supplementary Figure 1B-D. (**B**) Quantification of blood eosinophils via flow cytometry of patients with different forms of arthritis separated by individual disease activity and healthy controls (HC; *n*=12 healthy controls); Disease activity of axial spondyloarthritis (axSpA, *n*=35 patients) measured by axial spondyloarthritis disease activity score (ASDAS), psoriatic arthritis (PsA; *n*=29 patients) by disease activity in psoriatic arthritis score (DAPSA) and rheumatoid arthritis (RA; *n*=27 patients) by disease activity score 28 (DAS28). Violin plots show the kernel density distribution of all data points; the centre line indicates the median and the dashed lines the 25th and 75th percentiles. **(C)** Quantification of blood M-MDSCs via flow cytometry in HC (*n*=29 healthy controls) and different forms of arthritis: axSpA (*n*=25 patients), PsA (*n*=28 patients) and RA (*n*=27 patients) by individual disease activity scores. Violin plots show the kernel density distribution of all data points; the centre line indicates the median and the dashed lines the 25th and 75th percentiles. (**D**) Simple linear regression analysis of blood M-MDSCs via flow cytometry in axSpA (*n*=25 patients),

PsA (n=28 patients) and RA (n=27 patients). Statistical significance was tested using (**B-C**) two-sided unpaired one-way ANOVA with Tukey post hoc test; (**D**) R^2 indicating goodness of fit and P value testing whether the slope differs from zero. Source data are provided as a Source Data file.

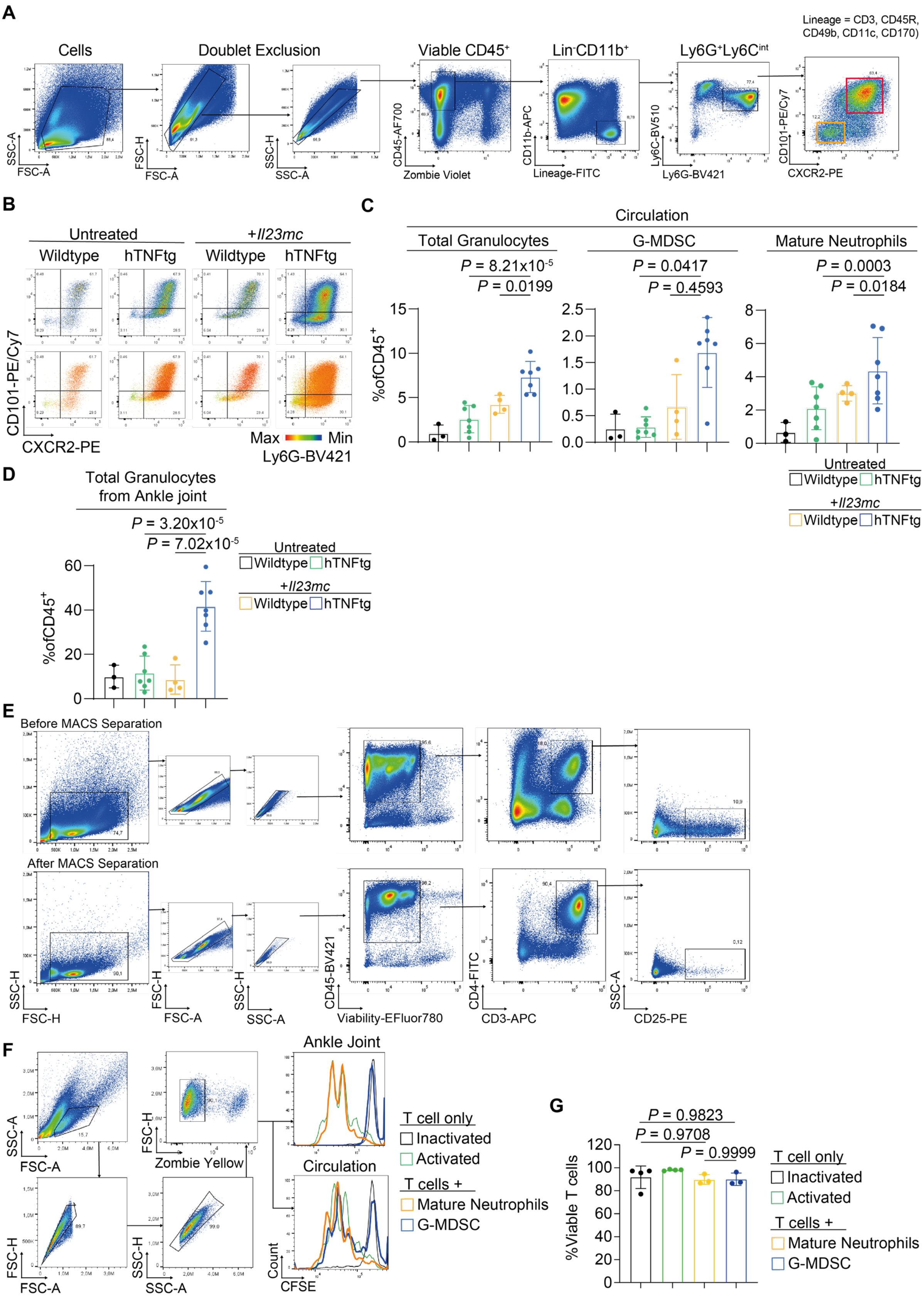
A
Lineage = CD3, CD45R, CD49b, CD11c, CD170)
Cells
Doublet Exclusion
Viable CD45+
Lin-CD11b+
Ly6G+Ly6Cint
SSC-A
FSC-A
FSC-H
FSC-A
SSC-H
SSC-A
CD45-AF700
Zombie Violet
CD11b-APC
Lineage-FITC
Ly6C-BV510
Ly6G-BV421
CD101-PE/Cy7
CXCR2-PE
B
Untreated
+Il23mc
Wildtype
hTNFtg
Wildtype
hTNFtg
CD101-PE/Cy7
CXCR2-PE
Max
Min
Ly6G-BV421
C
Circulation
Total Granulocytes
G-MDSC
Mature Neutrophils
P = 8.21x10-5
P = 0.0199
P = 0.0417
P = 0.4593
P = 0.0003
P = 0.0184
%ofCD45+
Untreated
Wildtype
hTNFtg
+Il23mc
Wildtype
hTNFtg
D
Total Granulocytes
from Ankle joint
P = 3.20x10-5
P = 7.02x10-5
%ofCD45+
Untreated
Wildtype
hTNFtg
+Il23mc
Wildtype
hTNFtg
E
Before MACS Separation
After MACS Separation
SSC-H
FSC-H
FSC-H
FSC-A
SSC-H
SSC-A
CD45-BV421
Viability-EFluor780
CD4-FITC
CD3-APC
SSC-A
CD25-PE
F
SSC-A
FSC-A
FSC-H
Zombie Yellow
Ankle Joint
Circulation
FSC-H
FSC-A
SSC-H
SSC-A
Count
CFSE
T cell only
Inactivated
Activated
T cells +
Mature Neutrophils
G-MDSC
G
P = 0.9823
P = 0.9708
P = 0.9999
%Viable T cells
T cell only
Inactivated
Activated
T cells +
Mature Neutrophils
G-MDSC

Supplementary Figure 2. Gating strategy and functional assessment of murine G-MDSCs in hTNFtg/*Il23mc* mice.

(A) Representative gating strategy to detect mature neutrophils (red) and G-MDSCs (yellow). This gating strategy applies to the data shown in Figure 2F, Figure 4E and Supplementary Figure 2B-D. **(B)** Representative plot of protein expression of CXCR2, CD101 and Ly6G analysed with flow cytometry. (**C**) Frequency of total granulocytes, G-MDSCs and mature neutrophils in circulation (spleen) from wildtype mice, hTNFtg mice, wildtype mice treated with *Il23mc* and hTNFtg mice treated with *Il23mc* after 6 days (*n*=3, 7, 4, 7 mice, pooled from three independent experiments) analysed by flow cytometry. (**D**) Frequency of total granulocytes from ankle joints from wildtype mice, hTNFtg mice, wildtype mice treated with *Il23mc* and hTNFtg mice treated with *Il23mc* after 6 days (*n*=3, 7, 4, 7 mice, pooled from three independent experiments) analysed by flow cytometry. (**E**) Representative flow cytometry plots showing CD25 expression among $CD4^+$ splenocytes before and after isolation of naïve $CD4^+CD25^-$ T cells by magnetic-activated cell sorting (MACS), assessed prior to co-culture. Numbers indicate the percentage of $CD25^+$ cells within the $CD4^+$ gate. These plots document the purity of the T cell preparations used for the suppression assays shown in Figure 2H and Supplementary Figure 2F-G. (**F**) Representative gating strategy for the T cell suppression assay followed by representative histograms of CFSE dilution, indicating proliferation of T cells with (green line) or without (black line) CD3/CD28 stimulation, co-cultured with mature neutrophils (orange) or G-MDSCs (dark blue). This gating strategy applies to the data shown in Figure 2H and Supplementary Figure 2G. (**G**) Viability of T cells determined by Live/Dead staining (Zombie Dye yellow) after culture with mature neutrophils or G-MDSCs derived from ankle joint of hTNFtg mice treated with *Il23mc* (*n*=3 mice each). Controls are IL-2 stimulated T cells only without CD3/CD28 and IL-2 stimulated T cells only with CD3/CD28 (*n*=4 mice each). Statistical significance was tested using two-sided unpaired one-way ANOVA with Tukey post hoc test (**C, D, G**). Source data are provided as a Source Data file.

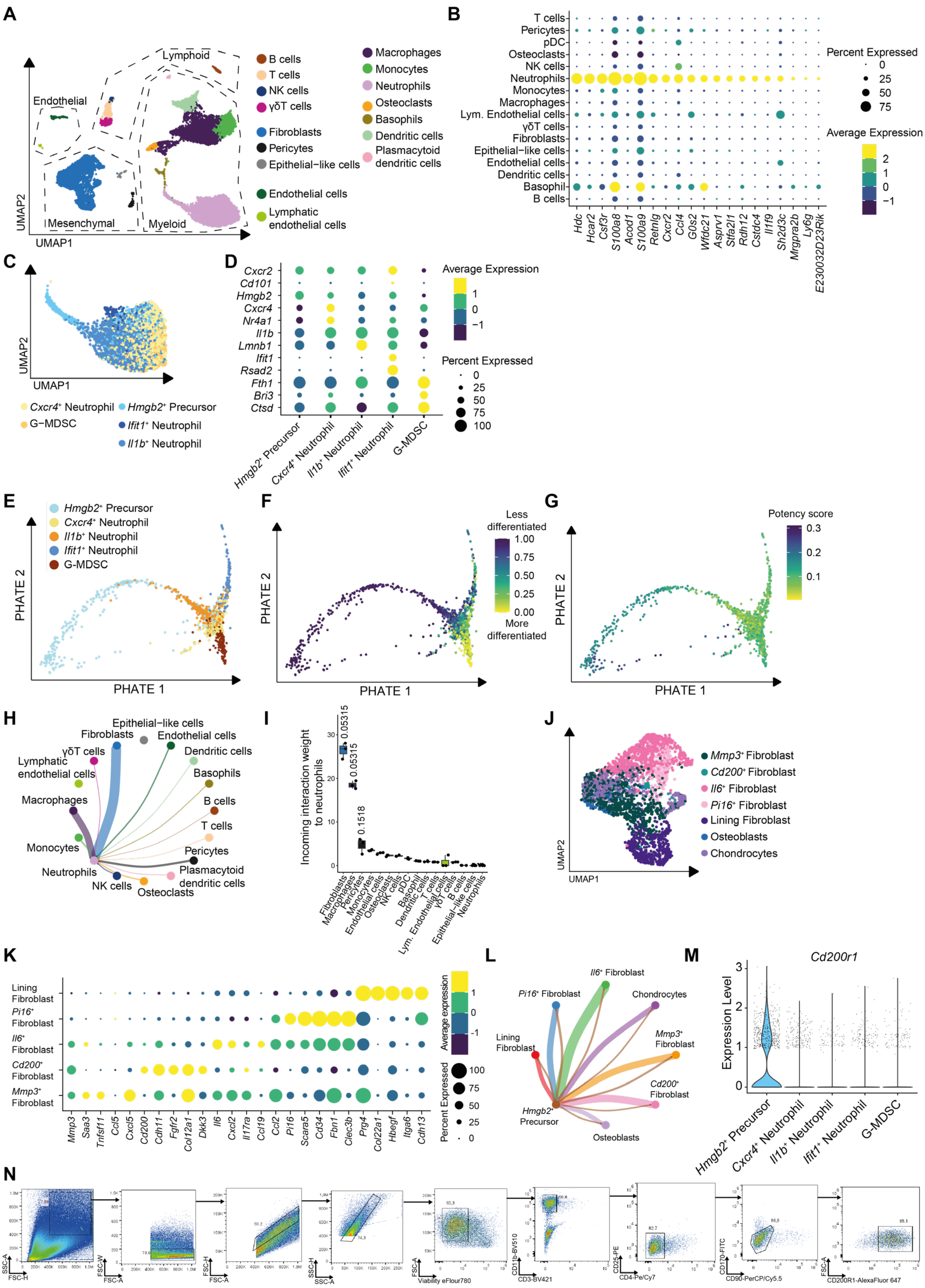
A
Lymphoid
Endothelial
Mesenchymal
Myeloid
UMAP2
UMAP1
B cells
T cells
NK cells
γδT cells
Fibroblasts
Pericytes
Epithelial−like cells
Endothelial cells
Lymphatic endothelial cells
Macrophages
Monocytes
Neutrophils
Osteoclasts
Basophils
Dendritic cells
Plasmacytoid dendritic cells
B
T cells
Pericytes
pDC
Osteoclasts
NK cells
Neutrophils
Monocytes
Macrophages
Lym. Endothelial cells
γδT cells
Fibroblasts
Epithelial−like cells
Endothelial cells
Dendritic cells
Basophil
B cells
Hdc
Hcar2
Csf3r
S100a8
Acod1
S100a9
Retnlg
Cxcr2
Ccl4
G0s2
Wfdc21
Asprv1
Stfa2l1
Rdh12
Cstdc4
Il1f9
Sh2d3c
Mrgpra2b
Ly6g
E230032D23Rik
Percent Expressed
0
25
50
75
Average Expression
2
1
0
−1
C
UMAP2
UMAP1
Cxcr4+ Neutrophil
Hmgb2+ Precursor
G−MDSC
Ifit1+ Neutrophil
Il1b+ Neutrophil
D
Cxcr2
Cd101
Hmgb2
Cxcr4
Nr4a1
Il1b
Lmnb1
Ifit1
Rsad2
Fth1
Bri3
Ctsd
Hmgb2+ Precursor
Cxcr4+ Neutrophil
Il1b+ Neutrophil
Ifit1+ Neutrophil
G−MDSC
Average Expression
1
0
−1
Percent Expressed
0
25
50
75
100
E
Hmgb2+ Precursor
Cxcr4+ Neutrophil
Il1b+ Neutrophil
Ifit1+ Neutrophil
G−MDSC
PHATE 2
PHATE 1
F
Less differentiated
1.00
0.75
0.50
0.25
0.00
More differentiated
PHATE 2
PHATE 1
G
Potency score
0.3
0.2
0.1
PHATE 2
PHATE 1
H
Epithelial−like cells
Fibroblasts
Endothelial cells
γδT cells
Dendritic cells
Lymphatic endothelial cells
Basophils
Macrophages
B cells
T cells
Monocytes
Pericytes
Plasmacytoid dendritic cells
Neutrophils
NK cells
Osteoclasts
I
Incoming interaction weight to neutrophils
0.05315
0.05315
0.1518
30
20
10
0
Fibroblasts
Macrophages
Pericytes
Monocytes
Endothelial cells
Osteoclasts
NK cells
pDC
Basophil
Dendritic cells
T cells
Lym. Endothelial cells
γδT cells
B cells
Epithelial−like cells
Neutrophils
J
Mmp3+ Fibroblast
Cd200+ Fibroblast
Il6+ Fibroblast
Pi16+ Fibroblast
Lining Fibroblast
Osteoblasts
Chondrocytes
UMAP2
UMAP1
K
Lining Fibroblast
Pi16+ Fibroblast
Il6+ Fibroblast
Cd200+ Fibroblast
Mmp3+ Fibroblast
Mmp3
Saa3
Tnfsf11
Ccl5
Cxcl5
Cd200
Cdh11
Fgfr2
Col12a1
Dkk3
Il6
Cxcl2
Il17ra
Ccl19
Ccl2
Pi16
Scara5
Cd34
Fbn1
Clec3b
Prg4
Col22a1
Hbegf
Itga6
Cdh13
Average expression
1
0
-1
Percent Expressed
100
75
50
25
0
L
Il6+ Fibroblast
Pi16+ Fibroblast
Chondrocytes
Lining Fibroblast
Mmp3+ Fibroblast
Cd200+ Fibroblast
Hmgb2+ Precursor
Osteoblasts
M
Cd200r1
Expression Level
3
2
1
0
Hmgb2+ Precursor
Cxcr4+ Neutrophil
Il1b+ Neutrophil
Ifit1+ Neutrophil
G-MDSC
N
SSC-A
FSC-H
FSC-W
FSC-A
FSC-H
FSC-A
SSC-H
SSC-A
FSC-A
Viability eFlour780
CD11b-BV510
CD3-BV421
CD25-PE
CD4-Pe/Cy7
CD170-FITC
CD90-PerCP/Cy5.5
FSC-A
CD200R1-AlexaFluor 647

Supplementary Figure 3. Single-cell analysis of neutrophil and fibroblast subsets in inflamed mouse joints.

(A) Uniform manifold approximation and projection (UMAP) visualization of major cell clusters from GSE253974 [1]. (**B**) Expression of the most relevant marker genes to describe neutrophils in the dataset from **A**. (**C, D**) UMAP visualization of neutrophil subsets (**C**) with expression of the most relevant marker genes to describe neutrophil subsets shown as dot plot (**D**). (**E-G**) Trajectory analysis of neutrophils in the serum transfer arthritis dataset. (**E**) PHATE [2] embedding of neutrophil subsets. (**F**) CytoTRACE [3] score projected onto the PHATE embedding, with higher scores indicating less differentiated cells. (**G**) CytoTRACE2 [4] absolute potency score per neutrophil subset. A high potency score indicates a precursor-like state, whereas a low score reflects a more mature, differentiated cell state. (**H-I**) Chord diagram (**H**) of the communication probability between neutrophils and other main cell types, inferred using CellChat [5] and cell interaction probability with neutrophils (**I**, n=3 samples each); Box plots show the median (centre line), the 25th and 75th percentiles (box bounds) and the minimum and maximum values (whiskers). Two-sided one-vs-rest Wilcoxon test. (**J-K**) UMAP visualization of fibroblast subsets (**J**) with expression of the most relevant marker genes to describe fibroblast subsets shown as dot plot (**K**). (**L**) Chord diagram of communication probability between *Hmgb2*$^{+}$ precursors and fibroblast subsets. **(M)** *Cd200r1* expression among neutrophil clusters, imputed using ALRA [6]. (**N**) Representative gating strategy to detect CD200R1^{+} precursors. This gating strategy applies to the data shown in Figure 4A. Source data are provided as a Source Data file.

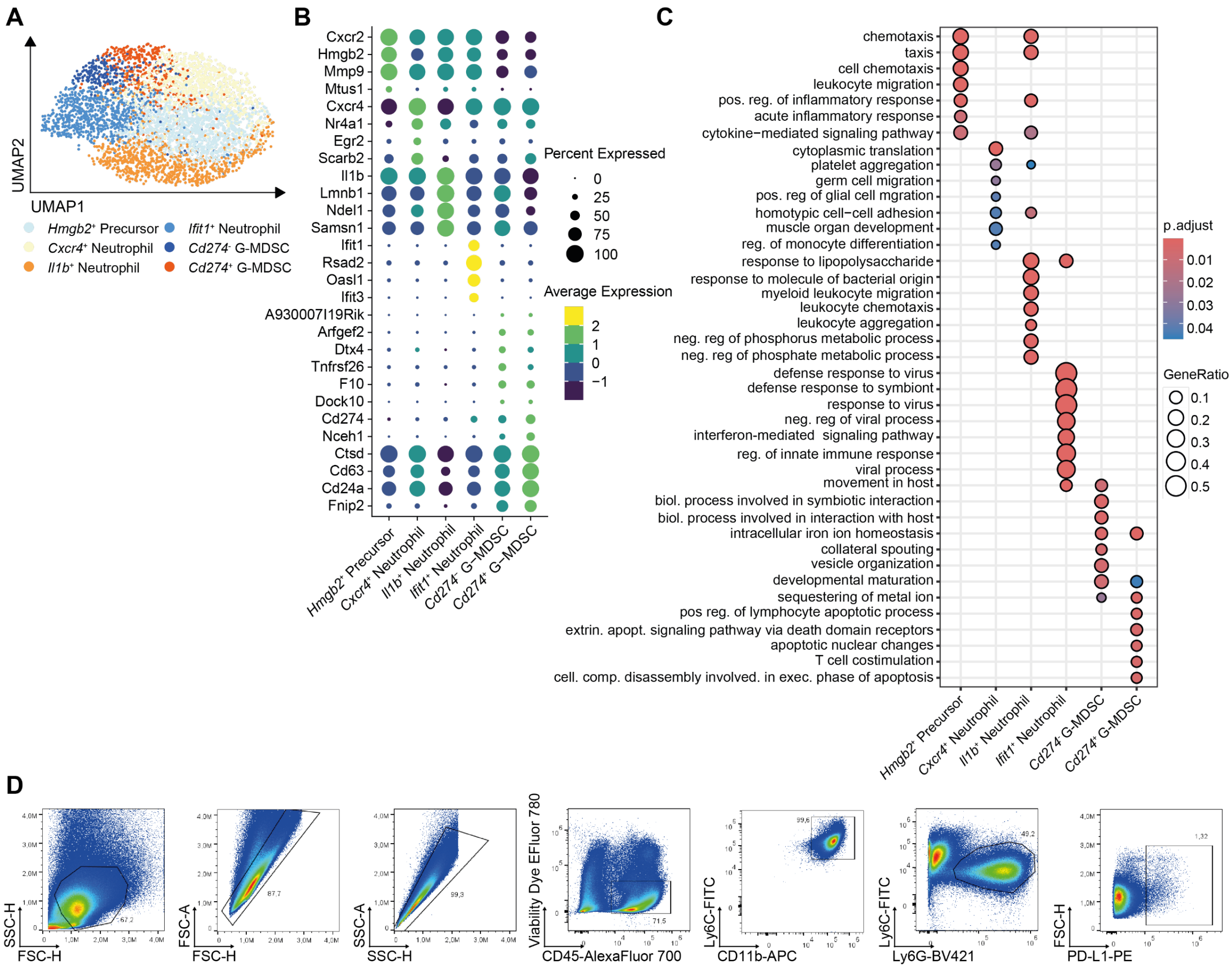

Supplementary Figure 4. Subclustering identifies a *Cd274*$^+$ G-MDSC subpopulation in mice.

(**A**) UMAP plot of granulocytic cells subclustered based on transcriptional heterogeneity. Cells were subclustered from the G-MDSC population identified in the cross-tissue neutrophil atlas (GSE165276 [7]). (**B**) Expression of the most relevant marker genes to describe subclustered G-MDSCs. (**C**) Significantly enriched gene ontology biological processes in the respective subcluster. (**D**) Representative gating strategy to detect PD-L1$^+$ G-MDSCs in murine ankle joints of hTNFtg mice treated with *Il23mc*. This gating strategy applies to the data shown in Figure 5F-G.

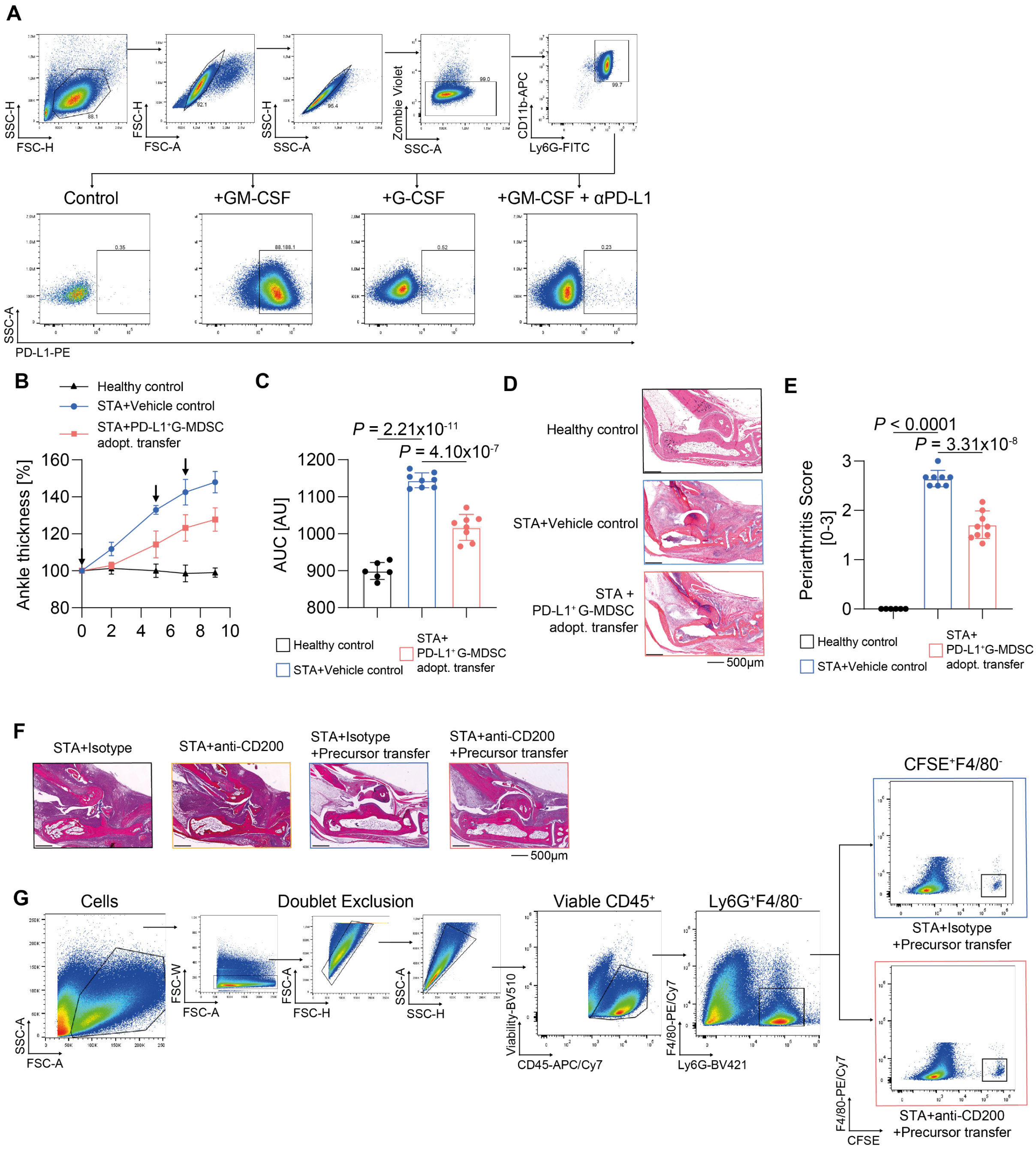

Supplementary Figure 5. Adoptive transfer of *in vitro* polarized G-MDSCs and CD177$^+$Ly6G$^+$ precursors in serum transfer arthritis.

(**A**) Representative FACS plots displaying PD-L1 expression of bone marrow-derived Ly6G$^+$ cells polarized with GM-CSF (PD-L1$^+$), G-CSF (PD-L1$^-$), GM-CSF with anti-PD-L1 blocking antibody or control. This gating strategy applies to the data shown in Figure 6A. (**B-E**) Measurement of arthritis after intraperitoneal serum transfer arthritis (STA) induction in the ankle joints (**B**) with area under the curve analysis (**C**), representative hematoxylin & eosin images of ankle joints (**D**) and semiquantitative periarthritis score (0-3, **E**) from healthy control mice, arthritic mice with vehicle control (PBS + 0.1%BSA, serum transfer) and arthritic mice (serum transfer) adoptively transferred intravenously with PD-L1$^+$ G-MDSCs induced *in vitro* by GM-CSF and CD200R1-agonist OX-110 (n=6, 8, 8 mice, data are from one experiment). Arrows in **B** indicate the timepoints of the adoptive transfers of G-MDSCs. Mean ± SD. (**F**) Representative hematoxylin & eosin images of ankle joints from arthritic mice treated with

isotype control rat IgG2a or anti-CD200 blocking antibody (OX-90) without cell transfer, and arthritic mice adoptively transferred with CFSE-labeled CD177$^+$Ly6G$^+$ precursors under isotype control or anti-CD200 blockade, respectively. (**G**) Representative gating strategy to detect adoptively transferred CFSE$^+$ cells in murine ankle joints, shown for recipients treated with isotype control or anti-CD200 blocking antibody (OX-90). This gating strategy applies to the data shown in Figure 6E. Statistical testing: (**C, E**) two-sided unpaired one-way ANOVA with Tukey post hoc test. Source data are provided as a Source Data file.

Supplementary Table S1: Patient characteristics for sacroiliac joint CD200/PD-L1 analysis, related to Figure 4B-C and 6F

Parameter	axSpA (*n*=8)
Age, years, mean (s.d.)	50.1 (9.8)
Female/Male, n/n	2/6
BASDAI, mean (s.d.)	6.3 (0.8)
Treatments	4 NSAID, 3 TNFi, 1 JAKi

Abbreviations: BASDAI, Bath Ankylosing Spondylitis Disease Activity Index; JAKi, Janus kinase inhibitor; n, number of individuals; NSAID, non-steroidal anti-inflammatory drug; TNFi, tumor necrosis factor inhibitor; s.d., standard deviation.

Supplementary Table S2: Selected genes for early Neutrotime signature [7]

Genes for early Neutrotime signature	Genes downregulated in early Neutrotime signature
Cd177	*Lyz2*
Mgst1	*Retnlg*
Pglyrp1	*Lgals3*
Ltf	*Mmp8*
Prdx5	*Ifitm6*
Lcn2	*Ly6g*
Ngp	
Adpgk	
Anxa1	
Arhgdib	
Ly6c2	
Camp	
Dstn	
Chil3	
Wfdc21	
Serpinb1a	

Supplementary References

1. Swarnkar, G., Semenkovich, N.P., Arra, M., Mims, D.K., Naqvi, S.K., Peterson, T., Mbalaviele, G., Wu, C.L., and Abu-Amer, Y. (2024). DNA hypomethylation ameliorates erosive inflammatory arthritis by modulating interferon regulatory factor-8. Proc Natl Acad Sci U S A *121*, e2310264121. 10.1073/pnas.2310264121.
2. Moon, K.R., van Dijk, D., Wang, Z., Gigante, S., Burkhardt, D.B., Chen, W.S., Yim, K., Elzen, A.V.D., Hirn, M.J., Coifman, R.R., et al. (2019). Visualizing structure and transitions in high-dimensional biological data. Nat Biotechnol *37*, 1482–1492. 10.1038/s41587-019-0336-3.
3. Gulati, G.S., Sikandar, S.S., Wesche, D.J., Manjunath, A., Bharadwaj, A., Berger, M.J., Ilagan, F., Kuo, A.H., Hsieh, R.W., Cai, S., et al. (2020). Single-cell transcriptional diversity is a hallmark of developmental potential. Science *367*, 405–411. 10.1126/science.aax0249.
4. Kang, M., Gulati, G.S., Brown, E.L., Qi, Z., Avagyan, S., Armenteros, J.J.A., Gleyzer, R., Zhang, W., Steen, C.B., D'Silva, J.P., et al. (2025). Improved reconstruction of single-cell developmental potential with CytoTRACE 2. Nat Methods *22*, 2258–2263. 10.1038/s41592-025-02857-2.
5. Jin, S., Plikus, M.V., and Nie, Q. (2025). CellChat for systematic analysis of cell-cell communication from single-cell transcriptomics. Nat Protoc *20*, 180–219. 10.1038/s41596-024-01045-4.
6. Linderman, G.C., Zhao, J., Roulis, M., Bielecki, P., Flavell, R.A., Nadler, B., and Kluger, Y. (2022). Zero-preserving imputation of single-cell RNA-seq data. Nat Commun *13*, 192. 10.1038/s41467-021-27729-z.
7. Grieshaber-Bouyer, R., Radtke, F.A., Cunin, P., Stifano, G., Levescot, A., Vijaykumar, B., Nelson-Maney, N., Blaustein, R.B., Monach, P.A., Nigrovic, P.A., and ImmGen, C. (2021). The neutrotime transcriptional signature defines a single continuum of neutrophils across biological compartments. Nat Commun *12*, 2856. 10.1038/s41467-021-22973-9.
