# Supplementary material for: CD200+ fibroblasts prime the immunoregulatory function of granulocytic myeloid-derived suppressor cells in inflammatory arthritis
Source: Nat Commun. 2026 Oct 13;17:10746. doi: 10.1038/s41467-026-78351-w (PMC13661617; doi:10.1038/s41467-026-78351-w)
Supplement: Supplementary file 2 [file 41467_2026_78351_MOESM2_ESM.pdf]

nature portfolio

Peer Review File

CD200+ fibroblast prime the immunoregulatory function of granulocytic myeloid-derived suppressor cells in inflammatory arthritis

Corresponding Author: Professor Andreas Ramming

This file contains all reviewer reports in order by version, followed by all author rebuttals in order by version.

Version 0:

Reviewer comments:

Reviewer #1

(Remarks to the Author)
This study by Angeli et al. investigates the role of CD200+ fibroblasts and granulocytic myeloid-derived suppressor cells (G-MDSCs) in axSpA. Mechanistic studies were performed in mouse models of inflammatory arthritis (hTNFtg mice injected with IL-23 minicircles, K/BxN serum transfer arthritis), which demonstrate that Hmgb2+ myeloid precursors acquire an immunosuppressive phenotype in situ under the influence of CD200+ fibroblasts signaling through CD200R expressed by these precursors. The experimental design of these studies is robust and methodologically sound. Additional human studies were performed with peripheral blood and biopsy specimens from the spine and SI joints of patients with axSpA and controls with degenerative disease. It is concluded that the CD200 - G-MDSC mechanism is defective in axSpA.
The authors deserve credit for this effort, as studies using diseased tissue in axSpA are difficult to perform and very rarely done. Nonetheless, the human experiments constitute the weaker part of the study. Considering the potential significant impact of this report, additional clarification and supporting evidence is needed to ensure that the conclusions are fully substantiated by the data presented.

Major comments:
1. The origin of the human biopsy specimens is insufficiently described. Which approach/technique was used for the CT-guided biopsies? Are the specimens derived from bone marrow or from another part of the SI joint? How was the "inflamed region of interest" defined (presumably based on the presence of bone marrow edema on MRI)?
2. The abstract states that “if CD200 expression in tissues is diminished, such as in axial spondyloarthritis, a chronic inflammatory joint disease, this physiologic priming is impaired and, as compensatory mechanism, circulating G-MDSCs are increased.”
However, the manuscript does not provide direct evidence for the diminished expression of CD200 in axSpA. Fig. 4G only shows a negative correlation between the abundance of podoplanin+CD200+ cells in the axSpA samples and BASDAI scores, without comparison to non-axSpA controls. What is the hypothetical compensatory mechanism leading to the observed increased frequency of circulating G-MDSCs in patients with axSpA?
3. How does the proposed CD200 - G-MDSC axis fit into the broader pathogenetic framework of axSpA? Why would this mechanisms be particularly relevant to axSpA and the SI joints? Have the authors considered that the presumed (but not demonstrated) reduction in CD200 expression is a consequence rather than the cause of chronic inflammation?
4. A higher number of CD33+lin- G-MDSCs in axSpA biopsy specimens is shown compared to controls (Fig. 1G). The authors attribute this to reduced CD200 expression. This interpretation appears inconsistent with their conclusion that CD220R agonism affects the differentiation and function but not the number of G-MDSCs (Fig. 5E), as the authors themselves emphasize. Moreover, why does the accumulation of G-MDSCs “suggest that G-MDSCs have lost their suppressive function”?

Minor comments:
- Mice: “…were maintained on a C56Bl/5N background”. This is likely a typo (C57BL/6N being correct). What are C57BL/6Rj mice? Please review strain nomenclature and provide information on the origin of the animals (vendor)?
- How much anti-CD200R antibody was injected into the mice? How often was K/BxN serum injected into recipient animals?
- The procedure of isolating cells from murine ankle joints would benefit from additional detail. Please explain such so that other investigators can reproduce the experiment.

- The gating strategy (Fig. S1A) contains two rows. What is the difference? Is one for mouse and the other for human specimens? Please explain in the figure/legend.
- Which types of plates and which antibody clones/concentrations were used for the T cell stimulation assay?
- How was the region of interest defined for the enumeration of G-MDSCs in human tissue? The H&E stains in Fig. 1F look hugely different. (The images are also very small.)
- Fig. 2 legend: "Representative images of hind paws and MRI scans of ankle joints T1stir sequence (MRIT1w CE)" Please review for accuracy. Based on the methods, the imaging was done with gadolinium administration, i.e. using T1w CE sequences (= T1 weighted contrast enhanced). STIR is a T2 weighted fat-suppressed sequence that does not rely on gadolinium administration.
- What is the meaning of the arrows in Fig. 2D?
- The labeling of the X axes in Fig. 2H is somewhat confusing. I would also suggest to select a color palette in Fig. 2 and Fig. 5 that provides a little more contrast than the shades of grey and blue currently used (see Rauber et al. Nat Immunol 2024).
- Panel I to L in Fig. 5 use the abbreviation STA, which likely means serum transfer arthritis, but is not explained in the legend or text.

Reviewer #2

(Remarks to the Author)
In this manuscript by Angeli et al, the authors propose that G-MDSCs exert their immunoregulatory properties in inflammatory joints in axSpa, via interactions with CD200+ resident fibroblasts.
Although the concept is novel, and the findings may hold significant translational implications, I believe that the conclusions are not strongly supported by the data, and several overinterpretations appear in the manuscript. Specifically, I don't see direct evidence for the differentiation of precursor Hmgb2+ cells into GMDSCs in vivo. In addition, the manuscript lack data demonstrating that the immunoregulatory role of GMDSCs is influenced by the CD200/CD200R axis (for example through ablation of the pathway in the specific cell subsets since CD200R1 agonist treatment may have pleiotropic effects). Finally, the focus on fibroblasts over other cells (i.e macrophages) is not experimentally justified.

Major comments
• In figure 1E,F is expression of CD33+Lin- enough to characterize the G-MDSCs? What staining the authors would be perform to distinguish GMDSCs from neutrophils?
• In Figure 2G the authors annotate G-MDSCs as CXCR2 and Gr1….these markers have general expression pattern, with CXCR2 to be expressed also in monocytes while the Gr1 antibody detects both Ly6G and Ly6C.
• The results of the in vitro suppression of T cell proliferation are elegantly presented. However, what would be interesting to be demonstrated is whether tissue G-MDSCs promote apoptosis of T cells or just inhibit their proliferation. What about their activation (i.e CD44)? Also the CD4+ T cells used in this experiments were depleted for Tregs? This is important considering that MDSCs may also instruct Treg cell function, which in turn may affect CD4+ T cell proliferation
• It seems that Hmgb2+ cells are abundant in blood of healthy individuals, as well as arthritic blood and arthritic joints (up to 50% of total granulocytes). Was that finding expected? Are these indeed precursors? Do they have mature granules as compared to il1b+ neutrophils? Did the authors validate these results in their mouse model?
• In Figure 4A, macrophages appear as another strong communication partner with neutrophils. Why the authors focused only in fibroblasts, ignoring macrophages? Also in 4D I don't see any difference in G-MDSCs compared to other neutrophil subsets. Overall, I found these data analyzed with a strong bias.
• It would be of interest to examine colocalization of Hmgb2+ cells with macrophages in SIJ
• What is the suppressive capacity of PDL1+ GMDSCs over PDL1- cells in vitro? In the Fog5I, transfer of control GMDSCs PDL1- is missing.

Minor comments
In Figure legend 2 GMDSCs are referred as Ly6Glow
The colors of boxes used in several figures to annotate the different groups, are not clearly distinguished

Reviewer #3

(Remarks to the Author)
This is an interesting study and I commend the authors on trying to understand the biology at the human SpA joint where obtaining tissue is very difficult. However I have a number of questions and a fundamental issue with the mouse work in this paper. The model run is a TNF-transgenic IL-23 minicircle but the authors draw a lot of their conclusions from the re-analysis of sinle cell experoments from different mosue models with differing types of arthritis. Why was there not a single cell analysis of the mice in the model the authors ran for this study.

My specific additional specific points are:

1- For the peripheral blood analysis in fig11, most patients have relatively low disease activity and are on anti TNF, how does this justify use of a TNF driven mouse mdoel?
2- Statement around "These data suggest that G-MDSCs have lost their suppressive function in patients with axSpA" is not suppoted by the data.
3- Why minicircle model and not SKG or B27 Transgenic rat?

4- In mouse model Fig 2: Joint GMDCs are able to supress, is this via PDL1? functional experiments with and PDL1 would be very informative.
5- Fig 3, why not analyse the Neutrophils from the model used in the experiment?
6- Fig 4, what is relevance of hmgb2 to human neutrophils?
7- Fig 4G, data presented from 8 patients, but the table has only 7 patients. Mean BASDAI on the figure is ~4.8 but in the table it is 6.3. Are these the same patients or from a different cohort? please explain the discrepancy
8- Fig 5 subclustering of GMDCs, which data is this from, the serum transfer or the TNF TG with IL23 minicircle?
9- On CD200-PDL1 axis…The key experiment here is to block PDL1 in this system as an additional control in fig 5A
10- Was the adoptive transfer into the joint or IV?
11- Fig 5M, Where do these patients come from? There are 8 again, not 7, the same 8 as fig 4G or the patients from the table?
12- Fig 5M, how is the CD200 niche defined? I'm not sure the immunoflouresence data presents suppots the co-localisation of CD200 and PDL1

Version 2:

Reviewer comments:

Reviewer #1

(Remarks to the Author)
1. In the biopsy technique published by Egund et al., the biopsy needle penetrates the SI joints at a perpendicular angle, thereby yielding tissue from subchondral bone marrow as well as the iliac and sacral bone plates. The authors explain that the biopsy site was chosen based on the presence of bone marrow edema on MRI. This suggests that the subchondral bone marrow was targeted rather than the SI joint. Please state explicitly which tissue(s) were sampled and reconcile this with the MRI-based site-selection criterion. An example CT image of the placed biopsy needle would be highly informative for the reader.
2. The animal data supporting the CD200-CD200R-G-MDSC axis are compelling. However, I remain unconvinced that the human data (tissue CD200 levels, circulating G-MDSC numbers) establish the same relationship in axSpA - the rebuttal and revised discussion do not fully resolve this. I would ask that the discussion states this limitation more plainly: that the human observations are consistent with, but do not establish, the mechanism demonstrated in mice. This is a question the field will need to resolve going forward.
3. For clarity, I suggest the following change in the abstract: "CD200R agonists can restore PD-L1 expression in G-MDSCs and control axial and peripheral joint inflammation in vivo, whereas…" => "CD200R agonists can restore PD-L1 expression in G-MDSCs and control axial and peripheral joint inflammation in vivo in mice, whereas…" The addition of "in mice" is critical, as the previous sentence in the abstract describes findings in human axSpA while the mechanistic data are from mice, which may or may not apply to human disease.

Reviewer #2

(Remarks to the Author)
The authors of this manuscript have done a commendable job with their revision, and successfully addressed my concerns. I still feel that they should report the Treg cell frequencies at the end of their suppression assay, especially since they report that Treg cells are included in the assay based on the kit they use for CD4+ T cell isolation. This is an important point considering the dominant suppression that Treg cells may exert.

Reviewer #3

(Remarks to the Author)
I thank the authors for a their comprehensive resposne to my comments and the revision of the manuscript. In particular I think the additonal experiments elucidating the mechanistic role of PDL1 improve the paper. I take on board the explanation rgearding the re-analysis of the data from the K/BxN serum transfer arthritis model by Grieshaber-Bouyer et al, but given that this model to my knowledge does not feature axial arhtritis and this is fundamentally a paper about axial inflammation I am still struggling with the fact this data features prominently in 3 out of the 6 main figures in the paper.

Open Access This Peer Review File is licensed under a Creative Commons Attribution 4.0 International License, which permits use, sharing, adaptation, distribution and reproduction in any medium or format, as long as you give appropriate credit to the original author(s) and the source, provide a link to the Creative Commons license, and indicate if changes were made.
In cases where reviewers are anonymous, credit should be given to 'Anonymous Referee' and the source.
The images or other third party material in this Peer Review File are included in the article's Creative Commons license, unless indicated otherwise in a credit line to the material. If material is not included in the article's Creative Commons license and your intended use is not permitted by statutory regulation or exceeds the permitted use, you will need to obtain permission directly from the copyright holder.
To view a copy of this license, visit https://creativecommons.org/licenses/by/4.0/

Response to Reviewers

CD200+ Fibroblast Interactions Prime the Immunoregulatory Function of Granulocytic Myeloid-Derived Suppressor Cells

We thank the reviewers for their thoughtful evaluation of our manuscript. Below, we provide point-by-point responses to each comment. Reviewer comments are in *italics* and our responses follow in regular text. Changes to the manuscript are highlighted in yellow.

Reviewer #1

> *This study by Angeli et al. investigates the role of CD200+ fibroblasts and granulocytic myeloid-derived suppressor cells (G-MDSCs) in axSpA. Mechanistic studies were performed in mouse models of inflammatory arthritis (hTNFtg mice injected with IL-23 minicircles, K/BxN serum transfer arthritis), which demonstrate that Hmgb2+ myeloid precursors acquire an immunosuppressive phenotype in situ under the influence of CD200+ fibroblasts signaling through CD200R expressed by these precursors. The experimental design of these studies is robust and methodologically sound. Additional human studies were performed with peripheral blood and biopsy specimens from the spine and SI joints of patients with axSpA and controls with degenerative disease. It is concluded that the CD200 - G-MDSC mechanism is defective in axSpA. The authors deserve credit for this effort, as studies using diseased tissue in axSpA are difficult to perform and very rarely done. Nonetheless, the human experiments constitute the weaker part of the study. Considering the potential significant impact of this report, additional clarification and supporting evidence is needed to ensure that the conclusions are fully substantiated by the data presented.*

We would like to thank the reviewer for this precise and concise summary of our study and are eager to address the concerns raised.

Major Comment 1

> *"The origin of the human biopsy specimens is insufficiently described. Which approach/technique was used for the CT-guided biopsies? Are the specimens derived from bone marrow or from another part of the SI joint? How was the 'inflamed region of interest' defined (presumably based on the presence of bone marrow edema on MRI)?"*

Response: We thank the reviewer for highlighting this important methodological detail. We have substantially expanded the Methods section to clarify the biopsy procedure:

Briefly, MRI was performed prior to biopsy to identify regions of active inflammation (bone marrow edema) in the sacroiliac joint. CT-guided needle biopsy was then performed by experienced interventional radiologists following standard protocols (Egund et al., 2020), with needle placement confirmed by imaging prior to sample collection.

Major Comment 2

> *"The abstract states that "if CD200 expression in tissues is diminished, such as in axial spondyloarthritis, a chronic inflammatory joint disease, this physiologic priming is impaired and, as compensatory mechanism, circulating G-MDSCs are increased."*

However, the manuscript does not provide direct evidence for the diminished expression of CD200 in axSpA. Fig. 4G only shows a negative correlation between the abundance of podoplanin+CD200+ cells in the axSpA samples and BASDAI scores, without comparison to non-axSpA controls. What is the hypothetical compensatory mechanism leading to the observed increased frequency of circulating G-MDSCs in patients with axSpA?"

Response: We have revised the statement to: "[…] if the abundance of $CD200^+$ fibroblasts decreases with increasing disease activity […]". This accurately reflects our data showing a negative correlation between $CD200^+$ fibroblast abundance and disease activity (BASDAI), rather than implying a comparison to healthy tissue. Additionally, we acknowledge that direct comparison to non-axSpA controls would strengthen our conclusions. However, obtaining sacroiliac joint biopsies from healthy individuals is ethically not justifiable given the invasive nature of the procedure. Our data demonstrate that $CD200^+$ fibroblast abundance decreases with increasing disease activity within axSpA patients, supporting a disease activity-dependent reduction in $CD200^+$ cells.

We have substantially expanded the Discussion section highlighting the hypothetical compensatory mechanism leading to the increased frequency of circulating G-MDSCs: The increase in circulating G-MDSCs in axSpA likely reflects disease-specific alterations in myelopoiesis. Recent studies demonstrate that experimental spondyloarthritis is characterized by GM-CSF-driven dysregulation of hematopoietic stem cell activity, with bias toward granulocyte-monocyte progenitors (GMP) and extramedullary myelopoiesis in inflamed joints (Regan-Komito et al., 2020). Serum GM-CSF levels are elevated in axSpA patients and persist despite anti-TNF therapy (Papagoras et al., 2022). Transcriptomic analyses of axSpA monocytes reveal a neutrophil-like phenotype with G-CSF response signatures, consistent with GMP-biased monocytopoiesis (Karow et al., 2021). Both GM-CSF and G-CSF are known cytokines for polarization of G-MDSC (Drujont et al., 2014; Marigo et al., 2010), supporting a mechanistic link between elevated serum cytokine levels and increased circulating G-MDSCs in axSpA. We propose that this enhanced myeloid output represents a compensatory attempt to generate immunoregulatory cells. However, when these precursors encounter tissue niches with diminished $CD200^+$ fibroblasts, they fail to acquire full suppressive function, perpetuating inflammation.

Major Comment 3

"How does the proposed CD200 - G-MDSC axis fit into the broader pathogenetic framework of axSpA? Why would this mechanism be particularly relevant to axSpA and the SI joints? Have the authors considered that the presumed reduction in CD200 expression is a consequence rather than the cause of chronic inflammation?"

Response: We appreciate the reviewer raising this important point regarding disease specificity and causality.

Why G-MDSCs and the CD200 axis are particularly relevant to axSpA and the SI joints: As detailed in our response to Major Comment 2, axSpA is characterized by pronounced alterations in myelopoiesis with GMP-biased hematopoiesis and neutrophil-dominated inflammation. G-MDSCs represent an endogenous regulatory mechanism specifically controlling neutrophilic inflammation, making them particularly relevant to axSpA pathophysiology. The sacroiliac joints, as the principal axial entheseal sites in axSpA, are subject to local tissue cues that activate resident mesenchymal cells and shape the immune

microenvironment (Cambre et al., 2018; Jacques et al., 2014; Schett et al., 2017). We propose that under such conditions the immunoregulatory capacity of tissue-resident fibroblasts becomes compromised, thereby impairing local G-MDSC priming at these sites.

Causality and timing of CD200$^+$ fibroblast reduction: We agree this is a critical question. To address whether reduced CD200 is cause or consequence of inflammation, we previously evaluated the kinetics of CD200$^+$ fibroblast abundance in the *Il23mc* model in our recent work (Raimondo et al., 2026). In this model, reduction of CD200$^+$ fibroblasts occurred already at day 7, before peak of inflammation at day 14, indicating that loss of CD200$^+$ fibroblasts precedes rather than follows the inflammatory peak. However, we do not aim to definitively resolve cause versus consequence in the present manuscript, as this does not affect the validity of the hypothesis or the translational implication of the axis: targeting CD200/CD200R signaling could break the inflammatory cycle regardless of its position in the causal chain.

Major Comment 4

> *"A higher number of CD33+lin- G-MDSCs in axSpA biopsy specimens is shown compared to controls. The authors attribute this to reduced CD200 expression. This interpretation appears inconsistent with their conclusion that CD200R agonism affects the differentiation and function but not the number of G-MDSCs. Moreover, why does the accumulation of G-MDSCs 'suggest that G-MDSCs have lost their suppressive function'?"*

Response: We thank the reviewer for identifying this apparent inconsistency, which we are happy to clarify.

Our data support a model where: (1) Hmgb2$^+$ precursors traffic to inflamed tissue independently of CD200 status; (2) CD200 signaling programs their functional maturation, namely PD-L1 expression and suppressive capacity, but not their recruitment; (3) in the absence of adequate CD200 signaling, G-MDSCs accumulate in tissue but remain dysfunctional, failing to resolve inflammation; (4) persistent inflammation drives continued precursor recruitment, creating a paradox of high G-MDSC numbers with impaired regulatory function. We have revised the text accordingly to state that accumulation "suggests impaired regulatory function" rather than "lost suppressive function."

This model is directly supported by our newly added adoptive transfer experiments, in which CFSE-labeled precursor cells homed to inflamed joints independently of CD200 status (revised Figure S6D), but anti-CD200 blockade during differentiation significantly impaired PD-L1$^+$ G-MDSC generation from the transferred precursor pool and abolished their therapeutic benefit (revised Figure 6E-H, S6C). These data provide direct *in vivo* evidence that CD200 signaling governs functional maturation rather than recruitment, fully reconciling the apparently contradictory observations across figures. Consistently, anti-CD200 blockade in the hTNFtg/*Il23mc* model reduced the number of PD-L1$^+$ G-MDSCs in the inflamed axial joint and aggravated arthritis (revised Figure 5I-K), confirming this mechanism within the disease model itself.

Minor Comments

"Mice: '...were maintained on a C56Bl/5N background'. This is likely a typo. What are C57BL/6Rj mice?"

Response: We apologize for this error. The correct strain is C57BL/6N. C57BL/6Rj refers to C57BL/6 mice from Janvier Labs (Rj = Janvier designation). We have corrected this and added vendor information.

"How much anti-CD200R antibody was injected into the mice? How often was K/BxN serum injected?"

Response: We have added these details: Anti-CD200R1 (OX-110) was administered at 100 μg/mouse intraperitoneally on days -1, 3, 5 and 7. K/BxN serum was injected as a single 200 μL intraperitoneal injection on day 0 (see Methods, page 29).

"The procedure of isolating cells from murine ankle joints would benefit from additional detail. Please explain such so that other investigators can reproduce the experiment"

Response: A detailed protocol for isolating cells from murine ankle joints is described in the Methods section "Tissue dissociation", page 32-33.

"The gating strategy (Fig. S1A) contains two rows. What is the difference? Is one for mouse and the other for human specimens? Please explain in the figure/legend.

Response: We have clarified in the legend that the top row shows the gating strategy to detect total granulocytes and eosinophils (panel 1) from peripheral blood. Panel 2 was used to detect and monocytic myeloid derived suppressor cells (M-MDSCs) as well as granulocytic myeloid derived suppressor cells (G-MDSCs) from peripheral blood.

"Which types of plates and which antibody clones/concentrations were used for the T cell stimulation assay?"

Response: We have added this information: T cell suppression assays were performed in 96-well U-bottom plates (Corning) coated with CD3ε (clone: 145-2C11, Biolegend, 5μg/ml) and CD28 (clone: 37.51, Biolegend, 3μg/ml) for 2 hours at 37°C.

"How was the region of interest defined for the enumeration of G-MDSCs in human tissue? The H&E stains in Fig. 1F look hugely different. (The images are also very small.)"

Response: We thank the reviewer for raising this point. The regions of interests in Fig. 1G were selected randomly and in a blinded manner, focusing on areas of cellular infiltration within the subchondral bone marrow. The variability in H&E appearance reflects in Fig. 1F the inherent histopathological heterogeneity of axSpA spinal and sacroiliac joint biopsies, which is characteristic of the disease. We have enlarged the images in revised Figure 1F.

"Fig. 2 legend: "Representative images of hind paws and MRI scans of ankle joints T1stir sequence (MRIT1w CE)" Please review for accuracy. Based on the methods, the imaging was done with gadolinium administration, i.e. using T1w CE sequences (= T1 weighted contrast enhanced). STIR is a T2 weighted fat-suppressed sequence that does not rely on gadolinium administration."

Response: We thank the reviewer for catching this error. The imaging was performed using T1-weighted contrast-enhanced (T1w CE) sequences after gadolinium administration, measuring contrast enhancement volume. We have corrected the figure legend accordingly.

"What is the meaning of the arrows in Fig. 2D?"

Response: The arrows indicate regions of contrast enhancement corresponding to active inflammation in the spine and sacroiliac joints. We have added this explanation to the figure legend.

" The labeling of the X axes in Fig. 2H is somewhat confusing. I would also suggest to select a color palette in Fig. 2 and Fig. 5 that provides a little more contrast than the shades of grey and blue currently used (see Rauber et al. Nat Immunol 2024)."

Response: We have revised the axis labels in Figure 2H for clarity and implemented a new color palette with improved contrast throughout all figures.

"Panel I to L in Fig. 5 use the abbreviation STA, which likely means serum transfer arthritis, but is not explained in the legend or text”

Response: We have added abbreviation STA as Serum Transfer Arthritis to the figure legend at first use. Note that these panels are now shown in Figure 6B-G following the split of the former Figure 5.

Reviewer #2

In this manuscript by Angeli et al, the authors propose that G-MDSCs exert their immunoregulatory properties in inflammatory joints in axSpa, via interactions with CD200+ resident fibroblasts.
Although the concept is novel, and the findings may hold significant translational implications, I believe that the conclusions are not strongly supported by the data, and several overinterpretations appear in the manuscript. Specifically, I don't see direct evidence for the differentiation of precursor Hmgb2+ cells into GMDSCs in vivo. In addition, the manuscript lack data demonstrating that the immunoregulatory role of GMDSCs is influenced by the CD200/CD200R axis (for example through ablation of the pathway in the specific cell subsets since CD200R1 agonist treatment may have pleiotropic effects). Finally, the focus on fibroblasts over other cells (i.e macrophages) is not experimentally justified.

Response: We thank the reviewer for acknowledging the novelty of our findings and the potential translational implications of our work. We appreciate the constructive criticism and agree that direct mechanistic evidence was needed to strengthen our conclusions. We have carefully addressed all concerns raised and are confident that the new data substantially support our proposed model.

***In vivo* differentiation of Hmgb2$^+$ precursors into G-MDSCs and CD200/CD200R specificity:**
We addressed both concerns by performing adoptive transfer experiments of CFSE-labeled neutrophil precursors (Grieshaber-Bouyer et al., 2021) into arthritic recipient mice treated with either anti-CD200 blocking antibody or isotype control. CFSE$^+$ cells recovered from inflamed joints directly demonstrate that transferred precursors differentiate into PD-L1$^+$ G-MDSCs *in vivo* (Figure 6E-H). Critically, anti-CD200 blockade significantly impaired this PD-L1$^+$ G-MDSC generation and abolished the therapeutic benefit of the transfer, whereas isotype-treated recipients showed significantly reduced arthritis. This experiment simultaneously provides direct *in vivo* evidence for precursor-to-G-MDSC differentiation and demonstrates that this process is specifically CD200-dependent, excluding pleiotropic effects of CD200R1 agonist treatment as an alternative explanation.
In addition, we now provide loss-of-function evidence within the axSpA model itself: anti-CD200 blockade in hTNFtg/*Il23mc* mice reduced PD-L1$^+$ G-MDSCs in the inflamed axial joint and aggravated disease (revised Figure 5I-K), thereby demonstrating that an intact CD200/CD200R1 axis is required for local immunoregulation in the disease context.

Focus on fibroblasts over other cells types including macrophages:

The CellChat analysis in Figures 4A and 4B demonstrates significantly higher interaction probabilities between neutrophils and fibroblasts compared to macrophages or any other cell type, providing an unbiased data-driven rationale for our focus on fibroblast-neutrophil crosstalk. A detailed response is provided in Major Comments 5 and 6.

Major Comment 1

"In figure 1E, F is expression of CD33+Lin- enough to characterize the G-MDSCs? What staining the authors would perform to distinguish GMDSCs from neutrophils?"

Response: We appreciate this important methodological question. The CD33+Lin-HLA-DR- phenotype represents the consensus human G-MDSC definition according to published guidelines (Bronte et al., 2016). However, we acknowledge the reviewer's concern regarding potential contamination with mature neutrophils. To address this, we have revised our immunofluorescence staining strategy to include CD16, thereby excluding neutrophils as recommended previously (Damuzzo et al., 2015) (Revised Figure 1G). We additionally expanded our patient cohort and re-analyzed all samples using this refined staining panel. The revised analysis yields similar results to our original staining strategy, confirming that neutrophil contamination was minimal and does not affect our conclusions.

Major Comment 2

> *"In Figure 2G the authors annotate G-MDSCs as CXCR2 and Gr1[…].these markers have general expression pattern, with CXCR2 to be expressed also in monocytes while the Gr1 antibody detects both Ly6G and Ly6C."*

Response: We thank the reviewer for this important methodological concern. We acknowledge that the Gr-1 antibody, which recognizes both Ly6G and Ly6C, lacks specificity for distinguishing granulocytic from monocytic populations.

To address this, we have revised our immunofluorescence staining to use Ly6G (clone 1A8) instead of Gr-1 for identifying granulocytic cells in tissue sections (Revised Figure 2G). Ly6G is highly specific for granulocytes and does not label monocytic cells. The revised analysis confirms our original findings: $CXCR2^{-}Ly6G^{+}$ G-MDSCs are enriched in inflamed axial tissue from hTNFtg/IL-23mc mice.

Major Comment 3

> *" The results of the in vitro suppression of T cell proliferation are elegantly presented. However, what would be interesting to be demonstrated is whether tissue G-MDSCs promote apoptosis of T cells or just inhibit their proliferation. What about their activation (i.e CD44)? Also the CD4+ T cells used in this experiments were depleted for Tregs? This is important considering that MDSCs may also instruct Treg cell function, which in turn may affect CD4+ T cell proliferation*

Response: These are excellent mechanistic questions. Regarding T_{reg} depletion: the naive $CD4^{+}$ T cell isolation kit (Miltenyi Biotec) used for our assays negatively selects for $CD25^{-}$ cells, thereby excluding $CD4^{+}CD25^{+}$ regulatory T cells.

In our assays, T cell viability was unchanged across all conditions while CFSE dilution demonstrated inhibition of $CD4^{+}$ T cell proliferation in the presence of tissue G-MDSCs, consistent with a cytostatic suppressive effect rather than induction of apoptosis (revised supplemental figure S2F) aligning with recent research (Jeong et al., 2025; Srivastava et al., 2010; Yan et al., 2020).

Major Comment 4

> *" It seems that Hmgb2+ cells are abundant in blood of healthy individuals, as well as arthritic blood and arthritic joints (up to 50% of total granulocytes). Was that finding expected? Are these indeed precursors? Do they have mature granules as compared to il1b+ neutrophils? Did the authors validate these results in their mouse model?*

Response: We appreciate this opportunity to clarify the nature of *Hmgb2*$^+$ precursors.

Expected proportion: The high proportion of *Hmgb2*$^+$ precursor cells in circulation is consistent with published literature. The "Neutrotime" continuum established by Grieshaber-Bouyer et al. (Grieshaber-Bouyer et al., 2021) demonstrated that *Hmgb2* marks early/immature neutrophils along the maturation spectrum. In inflammatory conditions, there is a well-documented increase in circulating immature neutrophils as part of "emergency granulopoiesis," characterized by an increased release of immature neutrophils into the circulation (Malengier-Devlies et al., 2021; Paudel et al., 2022). This phenomenon has been documented across diverse autoimmune and autoinflammatory diseases, where "reactive granulopoiesis" increases the proportion of immature neutrophils in the peripheral blood.

Evidence that these are precursors includes:

1. Highest early neutrophil signature score (Figure S3A).
2. Trajectory analysis placing them at the origin of differentiation paths (Figure 3C)
3. We have now additionally validated this finding in the whole tissue serum transfer arthritis dataset (GSE253974) using two complementary trajectory analysis methods, CytoTRACE (Gulati et al., 2020) and CytoTRACE2 (Kang et al., 2025), both of which robustly identify *Hmgb2*$^+$ cells as the least differentiated precursor population giving rise to mature neutrophil subsets including G-MDSCs (revised Figure S4E-G).

Finally, we observed an increase of Hmgb2$^+$CD200R1$^+$ precursor cells in our experimental axSpA model (Figure 4E).

Major Comment 5

"In Figure 4A, macrophages appear as another strong communication partner with neutrophils. Why the authors focused only in fibroblasts, ignoring macrophages? Also in 4D I don't see any difference in G-MDSCs compared to other neutrophil subsets. Overall, I found these data analyzed with a strong bias.

Response: We thank the reviewer for this comment and appreciate the opportunity to clarify our analytical approach.

Fibroblasts as dominant interaction partner: The CellChat analysis in Figures 4A and 4B is based on an unbiased computational assessment of cell-cell interactions in the dataset and demonstrates significantly higher interaction probabilities between neutrophils and fibroblasts compared to macrophages or any other cell type. Our subsequent focus on fibroblast-neutrophil crosstalk is therefore directly guided by these results rather than by a priori assumptions.

Clarification of Figure 4D: Figure 4D shows that *Cd200r1* expression is highest in *Hmgb2*$^+$ precursor cells, identifying these cells, rather than mature G-MDSCs, as the subset capable of receiving CD200 signals. This is consistent with our model in which precursors differentiate upon CD200/CD200R1 engagement to immunosuppressive G-MDSCs, rather than mature G-MDSCs being the direct signaling target.

Major Comment 6:

"It would be of interest to examine colocalization of Hmgb2+ cells with macrophages in SIJ"

Response: We thank the reviewer for this suggestion. Macrophage-neutrophil interactions are certainly interesting and may be explored in future studies. However, our CellChat analysis identified fibroblasts as the primary interaction partner for neutrophils overall and among the various interaction pathways, we focused on CD200-CD200R1 given its established role in immunosuppressive regulation (Gorczynski, 2005; Rauber et al., 2024). Thus, examining macrophage colocalization would not address the mechanism central to our study.

Major Comment 7:

> *"What is the suppressive capacity of PDL1+ GMDSCs over PDL1- cells in vitro? In the Fig5I, transfer of control GMDSCs PDL1- is missing."*

Response: These are critical questions that significantly strengthen our conclusions.

Suppressive capacity of PD-L1^{+} vs PD-L1^{-} cells *in vitro*: We thank the reviewer for this important question. To rigorously test PD-L1 dependence, we employed two complementary approaches. First, we took advantage of the differential PD-L1 expression induced by GM-CSF versus G-CSF polarization (Cerezo-Wallis et al., 2026) to compare the suppressive capacity of PD-L1^{+} and PD-L1^{-} bone marrow-derived Ly6G^{+} cells in a CFSE-based T cell proliferation assay (revised Figure S6A). GM-CSF-polarized, PD-L1^{+} G-MDSCs significantly suppressed T cell proliferation whereas G-CSF-polarized, PD-L1^{-} cells did not (revised Figure 6A). Second, anti-PD-L1 blockade was sufficient to abrogate the suppressive capacity of GM-CSF-polarized G-MDSCs, demonstrating that PD-L1 is functionally required for their immunoregulatory activity.

Adoptive transfer of PD-L1- G-MDSCs: We appreciate this suggestion. However, transferring sorted PD-L1^{-} G-MDSCs presents significant technical and biological limitations. Following cytokine treatment *in vitro*, the vast majority (>85%) of G-MDSCs express PD-L1 (Fig S6A), making isolation of sufficient PD-L1^{-} cells technically unfeasible while potentially selecting for non-responsive cells. Importantly, vehicle control (no cellular intervention) is the established standard for MDSC adoptive transfer studies, as this directly tests whether transferred cells provide therapeutic benefit (Fujii et al., 2013; Park et al., 2018; Wang et al., 2015). Our additional experiment using anti-CD200 blockade during precursor transfer (revised Figure 6E-H) provides mechanistic validation by demonstrating that CD200 signaling is necessary for G-MDSC differentiation and therapeutic function *in vivo*.

Minor Comments

> *" In Figure legend 2 GMDSCs are referred as Ly6Glow.*
>
> *The colors of boxes used in several figures to annotate the different groups, are not clearly distinguished"*

Response: We have corrected the Ly6G nomenclature for consistency and revised the color palette throughout for improved contrast and colorblind accessibility.

We have revised the color palette throughout all figures for improved contrast and colorblind accessibility.

Reviewer #3

> *"This is an interesting study and I commend the authors on trying to understand the biology at the human SpA joint where obtaining tissue is very difficult. However, I have a number of questions and a fundamental issue with the mouse work in this paper. The model run is a TNF-transgenic IL-23 minicircle but the authors draw a lot of their conclusions from the re-analysis of single cell experiments from different mouse models with differing types of arthritis. Why was there not a single cell analysis of the mice in the model the authors ran for this study."*

Response: We thank the reviewer for raising this important methodological point.

Biological justification for cross-model analysis: The K/BxN serum transfer model used in the published scRNA-seq dataset shares key pathomechanistic features with spondyloarthritis, including IL-17-driven inflammation and neutrophil-mediated pathology (Christensen et al., 2016; Shaw et al., 2016).

Animal ethics considerations: Re-analysis of publicly available single-cell datasets aligns with the 3R principles (Replacement, Reduction, Refinement) by maximizing scientific output from existing data and avoiding unnecessary animal experiments.

Functional validation in our model: Importantly, all functional experiments, including suppression assays and CD200R agonist treatment, were performed in our hTNFtg/IL-23mc model, directly validating the proposed mechanism in the relevant disease context. The scRNA-seq re-analysis served to generate hypotheses that we subsequently tested experimentally in our spondyloarthritis model.

Comment 1:

> *"For the peripheral blood analysis in fig11, most patients have relatively low disease activity and are on anti TNF, how does this justify use of a TNF driven mouse model?*

Response: We thank the reviewer for this question. We would respectfully point out that this observation actually supports rather than undermines the use of a TNF-driven model. The fact that patients achieve low disease activity under anti-TNF therapy demonstrates the central role of TNF in driving disease pathology in axSpA. Ethically, we cannot withhold effective treatment to study patients with high disease activity. The hTNFtg/IL-23mc model therefore provides an essential tool to investigate disease mechanisms under conditions of active, untreated inflammation that cannot be ethically studied in human patients. In this context, the mouse model complements rather than contradicts our human data.

Comment 2:

> *"Statement around 'These data suggest that G-MDSCs have lost their suppressive function in patients with axSpA' is not supported by the data."*

Response: We thank the reviewer for this comment, which was also raised by Reviewer 1. We have revised the statement to "These data suggest impaired regulatory function of G-MDSCs in patients with axSpA" and provide a detailed clarification of our model, including the newly added adoptive transfer experiments, in our response to Reviewer 1, Major Comment 4.

Comment 3:

"Why minicircle model and not SKG or B27 Transgenic rat?"

Response: We selected the hTNFtg/IL-23mc model for several reasons:

Molecular definition: The SKG model is a spontaneous arthritis model driven by a ZAP-70 mutation, which is molecularly less well-defined and more reminiscent of rheumatoid arthritis than spondyloarthritis (Sakaguchi et al., 2003). In contrast, the hTNFtg/IL-23mc model directly addresses the two key cytokine pathways most clearly implicated in SpA pathogenesis: TNF and IL-23/IL-17.

Axial involvement: Our hTNFtg/IL-23mc model is well-characterized by our group and recapitulates axial involvement, a hallmark of axSpA that is less prominent in the SKG model.

Species considerations: The HLA-B27 transgenic rat, while relevant to SpA, would introduce a third species into our study. This significantly complicates interpretation given species-specific differences in immune cell biology and limits the use of established reagents optimized for mouse studies.

Neutrophil biology: The TNF-driven model is well-established for studying neutrophil biology in inflammatory arthritis, aligning with the focus of our study.

Comments 4, 9:

"In mouse model Fig 2: Joint GMDCs are able to supress, is this via PDL1? functional experiments with and PDL1 would be very informative"

"On CD200-PDL1 axis…The key experiment here is to block PDL1 in this system as an additional control in fig 5A"

Response:

Suppressive capacity of joint G-MDSCs and PD-L1 dependence:

We thank the reviewer for these highly complementary suggestions, which directly align with experiments we have now performed. To directly test whether PD-L1 is functionally required for G-MDSC-mediated suppression, we polarized bone marrow-derived Ly6G^{+} cells with either GM-CSF or G-CSF and performed CFSE-based T cell proliferation assays. GM-CSF polarizes cells towards PD-L1^{+} G-MDSCs, whereas G-CSF-polarized cells do not express PD-L1 (revised Figure S6A)(Cerezo-Wallis et al., 2026). Importantly, PD-L1^{+} G-MDSCs significantly suppressed T cell proliferation whereas G-CSF-polarized PD-L1^{-} cells did not (revised Figure 6A). Critically, anti-PD-L1 blockade was sufficient to abrogate the suppressive capacity of GM-CSF-polarized G-MDSCs, demonstrating that PD-L1 is functionally required for immunosuppression.

***In vivo* anti-PD-L1 blockade:**

Regarding the *in vivo* anti-PD-L1 blocking experiment, we addressed the *in vivo* requirement of this axis by blocking CD200 upstream: anti-CD200 treatment in the hTNFtg/*Il23mc* model reduced PD-L1^{+} G-MDSCs in the axial joint and aggravated disease (revised Figure 5I-K). We refrained from systemic PD-L1 blockade because it significantly exacerbates joint inflammation, as demonstrated in the CIA model (Wood et al., 2024), which would confound

any G-MDSC-specific interpretation. For the direct mechanistic question, we therefore relied on the *in vitro* approach with defined cell populations (Figure 6A).

Comment 5:

"Fig 3, why not analyse the Neutrophils from the model used in the experiment?"

Response: We appreciate the reviewer's concern regarding the use of publicly available scRNA-seq datasets for hypothesis generation. Our experimental strategy was intentionally designed to leverage existing high-quality single-cell datasets for discovery, while reserving resource-intensive *in vivo* and functional validation for our disease-relevant hTNFtg/IL-23mc model. Performing *de novo* scRNA-seq of neutrophils from hTNFtg/IL-23mc mice would have substantially increased cost and animal usage without providing additional mechanistic insight, for the following reasons:

Pathophysiological relevance of the used dataset: The K/BxN model used for transcriptomic hypothesis generation shares key pathogenic features with spondyloarthritis, including IL-17–driven inflammation and a dominant role of neutrophil/myeloid cells in disease progression (Cho et al., 2016; Wipke & Allen, 2001). This makes it a suitable system for identifying conserved myeloid differentiation states and signaling pathways.

Ethical considerations and the 3R principles: Re-analysis of publicly available single-cell datasets maximizes scientific output while minimizing additional animal use, in accordance with the principles of Replacement, Reduction and Refinement.

Disease-specific functional validation: Importantly, all key functional experiments, including suppression assays, CD200R1 agonist treatment and anti-CD200 blockade, were performed in the hTNFtg/*Il23mc* model. These experiments directly demonstrate that the CD200/CD200R1 axis functionally reprograms G-MDSCs in a disease-relevant axSpA model, thereby validating and extending the transcriptomic predictions.

Taken together, our approach combines unbiased hypothesis generation with rigorous functional validation in the appropriate disease context, ensuring both mechanistic depth and translational relevance.

Comment 6:

"Fig 4, what is relevance of hmgb2 to human neutrophils?

Response: This is an important translational question. *HMGB2* (High Mobility Group Box 2) is a chromatin-associated protein highly expressed in immature cells (Grieshaber-Bouyer et al., 2021). In neutrophils, HMGB2 marks early/immature stages of differentiation in both mice and humans.

Recent transcriptomic profiling of human bone marrow neutrophil progenitors (Signoretto et al., 2025) confirms that HMGB2 is indeed expressed by immature neutrophil precursors **(**Response letter figure 1**).** Importantly, these HMGB2^{+} precursors co-express CD200R1, demonstrating their responsiveness to CD200 signaling. This validates our findings that tissue-infiltrating HMGB2^{+} neutrophil precursors can be programmed by CD200 to acquire the PD-L1^{+} immunosuppressive phenotype we describe in axSpA.

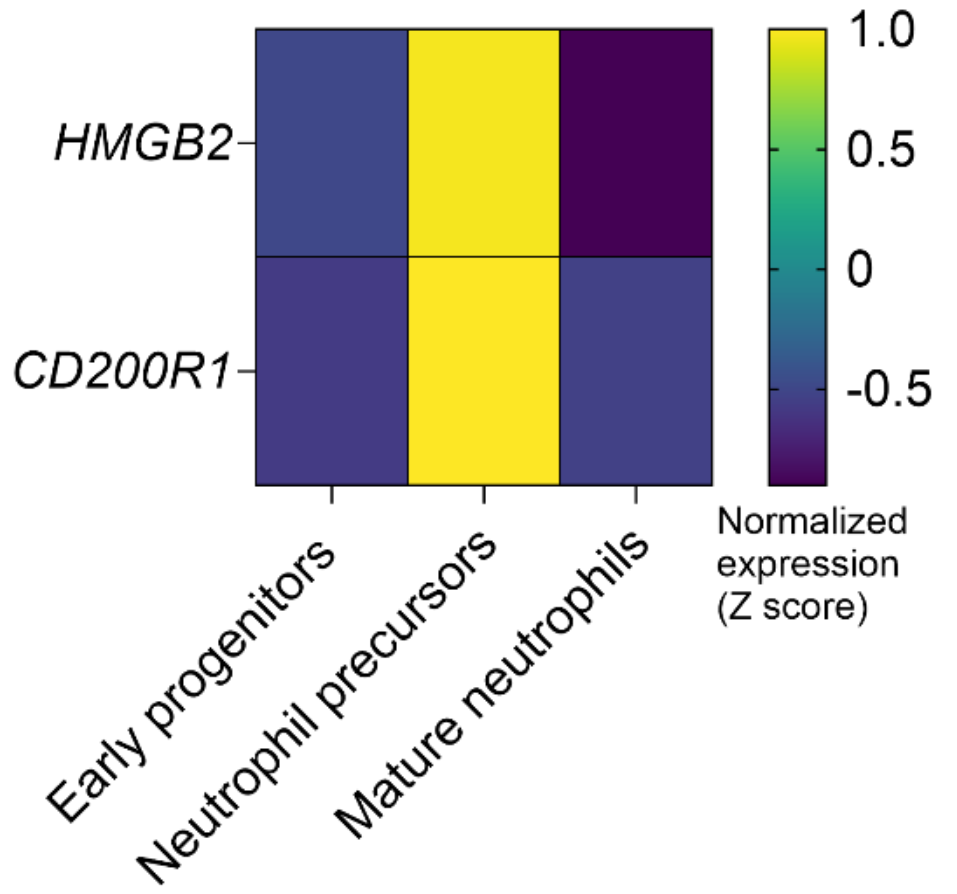

Response letter figure 1: Heatmap showing normalized expression (Z-scores) of *HMGB2* and *CD200R1* across human neutrophil developmental stages. Early progenitors = neutrophil-committed progenitors (NCPs 1-6); Neutrophil precursors = promyelocytes (PM), myelocytes (MY), metamyelocytes (MM), band cells (BC); Mature neutrophils = segmented neutrophil. Data reanalyzed from Signoretto et al., (GSE274002).

Comment 7, 11:

"Fig 4G, data presented from 8 patients, but the table has only 7 patients. Mean BASDAI on the figure is ~4.8 but in the table it is 6.3. Are these the same patients or from a different cohort?"

"Fig 5M, Where do these patients come from? There are 8 again, not 7, the same 8 as fig 4G or the patients from the table?"

Response: We sincerely thank the reviewer for identifying this discrepancy, which we have now resolved:

- Figure 4F/G data derive from CD200 staining analysis (n=8 patients), while Table 2 describes the tissue biopsy cohort for G-MDSC analysis (n=7 patients, now extended to n=8). These are overlapping but not identical cohorts.
- We have created Table S1 specifically for the CD200/PD-L1 analysis cohort (n=8) with corresponding clinical characteristics
- All tables have been audited for consistency with figure data
- Revised Figure 6I uses the same n=8 patients as Figure 4F/G (Table S1)

Comment 8:

"Fig 5 subclustering of GMDCs, which data is this from?"

Response: This subclustering is from the K/BxN serum transfer arthritis dataset (GSE253974). We have clarified this in the figure legend.

Minor Comment 10:

"Was the adoptive transfer into the joint or IV?"

Response: Adoptive transfer was performed intravenously (IV). This is stated in the Methods and we have added clarification to the figure legend.

Minor Comment 12:

" Fig 5M, how is the CD200 niche defined? I'm not sure the immunoflouresence data presents suppots the co-localisation of CD200 and PDL1"

Response: We thank the reviewer for this comment and apologize for any confusion caused by the original representation. We have removed the representative immunofluorescence image, as it was misleading rather than supportive of the quantitative analysis. To address the underlying point more rigorously, we have improved the Methods section with a more precise definition of the $CD200^+$ niche, defined as a 100 μm radius from $CD200^+$ cell centroids based on the typical range of paracrine signaling (Francis & Palsson, 1997). $PD\text{-}L1^+$ cells within this radius were classified as niche-associated. The improved quantitative analysis is now presented in revised Figure 6I.

References

Bronte, V., Brandau, S., Chen, S. H., Colombo, M. P., Frey, A. B., Greten, T. F., Mandruzzato, S., Murray, P. J., Ochoa, A., Ostrand-Rosenberg, S., Rodriguez, P. C., Sica, A., Umansky, V., Vonderheide, R. H., & Gabrilovich, D. I. (2016). Recommendations for myeloid-derived suppressor cell nomenclature and characterization standards. *Nat Commun*, *7*, 12150. https://doi.org/10.1038/ncomms12150

Cambre, I., Gaublomme, D., Burssens, A., Jacques, P., Schryvers, N., De Muynck, A., Meuris, L., Lambrecht, S., Carter, S., de Bleser, P., Saeys, Y., Van Hoorebeke, L., Kollias, G., Mack, M., Simoens, P., Lories, R., Callewaert, N., Schett, G., & Elewaut, D. (2018). Mechanical strain determines the site-specific localization of inflammation and tissue damage in arthritis. *Nat Commun*, *9*(1), 4613. https://doi.org/10.1038/s41467-018-06933-4

Cerezo-Wallis, D., Rubio-Ponce, A., Richter, M., Pitino, E., Kwok, I., Marteletto, G., Guanolema-Coba, A. C., Shih, C., Huang, R. K., Moraga, A., Bravo, N. B., Dore, S., Callejas, S., Aragones, D. G., Jimenez-Carretero, D., Martin, D., Ovadia, S., Vicanolo, T., Crainiciuc, G., . . . Ballesteros, I. (2026). Architecture of the neutrophil compartment. *Nature*, *649*(8098), 1003-1012. https://doi.org/10.1038/s41586-025-09807-0

Cho, W. S., Jang, E., Kim, H. Y., & Youn, J. (2016). Interleukin 17-expressing Innate Synovial Cells Drive K/Bxn Serum-induced Arthritis. *Immune Netw*, *16*(6), 366-372. https://doi.org/10.4110/in.2016.16.6.366

Christensen, A. D., Haase, C., Cook, A. D., & Hamilton, J. A. (2016). K/BxN Serum-Transfer Arthritis as a Model for Human Inflammatory Arthritis. *Front Immunol*, *7*, 213. https://doi.org/10.3389/fimmu.2016.00213

Damuzzo, V., Pinton, L., Desantis, G., Solito, S., Marigo, I., Bronte, V., & Mandruzzato, S. (2015). Complexity and challenges in defining myeloid-derived suppressor cells. *Cytometry B Clin Cytom*, *88*(2), 77-91. https://doi.org/10.1002/cyto.b.21206

Drujont, L., Carretero-Iglesia, L., Bouchet-Delbos, L., Beriou, G., Merieau, E., Hill, M., Delneste, Y., Cuturi, M. C., & Louvet, C. (2014). Evaluation of the therapeutic potential of bone marrow-derived myeloid suppressor cell (MDSC) adoptive transfer in mouse models of autoimmunity and allograft rejection. *PLoS One*, *9*(6), e100013. https://doi.org/10.1371/journal.pone.0100013

Egund, N., Sorensen, F. B., Ostgard, R., Loft, A. G., Boel, L. W. T., & Jurik, A. G. (2020). CT-guided transarticular biopsy of the sacroiliac joint: Technique and histomorphological results. A preliminary study. *Skeletal Radiol*, *49*(3), 453-460. https://doi.org/10.1007/s00256-019-03305-x

Francis, K., & Palsson, B. O. (1997). Effective intercellular communication distances are determined by the relative time constants for cyto/chemokine secretion and diffusion. *Proc Natl Acad Sci U S A*, *94*(23), 12258-12262. https://doi.org/10.1073/pnas.94.23.12258

Fujii, W., Ashihara, E., Hirai, H., Nagahara, H., Kajitani, N., Fujioka, K., Murakami, K., Seno, T., Yamamoto, A., Ishino, H., Kohno, M., Maekawa, T., & Kawahito, Y. (2013). Myeloid-derived suppressor cells play crucial roles in the regulation of mouse collagen-induced arthritis. *J Immunol*, *191*(3), 1073-1081. https://doi.org/10.4049/jimmunol.1203535

Gorczynski, R. M. (2005). CD200 and its receptors as targets for immunoregulation. *Curr Opin Investig Drugs*, *6*(5), 483-488. https://www.ncbi.nlm.nih.gov/pubmed/15912961

Grieshaber-Bouyer, R., Radtke, F. A., Cunin, P., Stifano, G., Levescot, A., Vijaykumar, B., Nelson-Maney, N., Blaustein, R. B., Monach, P. A., Nigrovic, P. A., & ImmGen, C.

(2021). The neutrotime transcriptional signature defines a single continuum of neutrophils across biological compartments. *Nat Commun*, *12*(1), 2856. https://doi.org/10.1038/s41467-021-22973-9

Gulati, G. S., Sikandar, S. S., Wesche, D. J., Manjunath, A., Bharadwaj, A., Berger, M. J., Ilagan, F., Kuo, A. H., Hsieh, R. W., Cai, S., Zabala, M., Scheeren, F. A., Lobo, N. A., Qian, D., Yu, F. B., Dirbas, F. M., Clarke, M. F., & Newman, A. M. (2020). Single-cell transcriptional diversity is a hallmark of developmental potential. *Science*, *367*(6476), 405-411. https://doi.org/10.1126/science.aax0249

Jacques, P., Lambrecht, S., Verheugen, E., Pauwels, E., Kollias, G., Armaka, M., Verhoye, M., Van der Linden, A., Achten, R., Lories, R. J., & Elewaut, D. (2014). Proof of concept: enthesitis and new bone formation in spondyloarthritis are driven by mechanical strain and stromal cells. *Ann Rheum Dis*, *73*(2), 437-445. https://doi.org/10.1136/annrheumdis-2013-203643

Jeong, H., Koh, J., Kim, S., Yim, J., Song, S. G., Kim, H., Li, Y., Lee, S. H., Chung, Y. K., Kim, H., Lee, C. H., Kim, H. Y., Keam, B., Lee, S. H., Chung, D. H., & Jeon, Y. K. (2025). Cell-intrinsic PD-L1 signaling drives immunosuppression by myeloid-derived suppressor cells through IL-6/Jak/Stat3 in PD-L1-high lung cancer. *J Immunother Cancer*, *13*(3). https://doi.org/10.1136/jitc-2024-010612

Kang, M., Gulati, G. S., Brown, E. L., Qi, Z., Avagyan, S., Armenteros, J. J. A., Gleyzer, R., Zhang, W., Steen, C. B., D'Silva, J. P., Schwab, J., Clarke, M. F., Chaudhuri, A. A., & Newman, A. M. (2025). Improved reconstruction of single-cell developmental potential with CytoTRACE 2. *Nat Methods*, *22*(11), 2258-2263. https://doi.org/10.1038/s41592-025-02857-2

Karow, F., Smiljanovic, B., Grun, J. R., Poddubnyy, D., Proft, F., Talpin, A., Hue, C., Boland, A., Deleuze, J. F., Garchon, H. J., Ergenc, I., De Craemer, A. S., Erben, U., Haupl, T., Elewaut, D., Breban, M., Grutzkau, A., & Syrbe, U. (2021). Monocyte transcriptomes from patients with axial spondyloarthritis reveal dysregulated monocytopoiesis and a distinct inflammatory imprint. *Arthritis Res Ther*, *23*(1), 246. https://doi.org/10.1186/s13075-021-02623-7

Malengier-Devlies, B., Metzemaekers, M., Wouters, C., Proost, P., & Matthys, P. (2021). Neutrophil Homeostasis and Emergency Granulopoiesis: The Example of Systemic Juvenile Idiopathic Arthritis. *Front Immunol*, *12*, 766620. https://doi.org/10.3389/fimmu.2021.766620

Marigo, I., Bosio, E., Solito, S., Mesa, C., Fernandez, A., Dolcetti, L., Ugel, S., Sonda, N., Bicciato, S., Falisi, E., Calabrese, F., Basso, G., Zanovello, P., Cozzi, E., Mandruzzato, S., & Bronte, V. (2010). Tumor-induced tolerance and immune suppression depend on the C/EBPbeta transcription factor. *Immunity*, *32*(6), 790-802. https://doi.org/10.1016/j.immuni.2010.05.010

Papagoras, C., Tsiami, S., Chrysanthopoulou, A., Mitroulis, I., & Baraliakos, X. (2022). Serum granulocyte-macrophage colony-stimulating factor (GM-CSF) is increased in patients with active radiographic axial spondyloarthritis and persists despite anti-TNF treatment. *Arthritis Res Ther*, *24*(1), 195. https://doi.org/10.1186/s13075-022-02888-6

Park, M. J., Lee, S. H., Kim, E. K., Lee, E. J., Baek, J. A., Park, S. H., Kwok, S. K., & Cho, M. L. (2018). Interleukin-10 produced by myeloid-derived suppressor cells is critical for the induction of Tregs and attenuation of rheumatoid inflammation in mice. *Sci Rep*, *8*(1), 3753. https://doi.org/10.1038/s41598-018-21856-2

Paudel, S., Ghimire, L., Jin, L., Jeansonne, D., & Jeyaseelan, S. (2022). Regulation of emergency granulopoiesis during infection. *Front Immunol*, *13*, 961601. https://doi.org/10.3389/fimmu.2022.961601

Raimondo, M. G., Mohammadian, H., Angeli, M. R., Alivernini, S., Fedorchenko, V., Huang, K., Demmler, R., Rhein, P., Xu, C., Li, Y. N., Micheroli, R., Winter, Z., Rigau, A. R., Anchang, C. G., Soare, A., Luber, M., Labinsky, H., Chang, J., Gunther, C., . . . Ramming, A. (2026). Skin-derived myeloid precursors and joint-resident fibroblasts spread psoriatic disease from skin to joints. *Nat Immunol*, *27*(1), 35-47. https://doi.org/10.1038/s41590-025-02351-z

Rauber, S., Mohammadian, H., Schmidkonz, C., Atzinger, A., Soare, A., Treutlein, C., Kemble, S., Mahony, C. B., Geisthoff, M., Angeli, M. R., Raimondo, M. G., Xu, C., Yang, K. T., Lu, L., Labinsky, H., Saad, M. S. A., Gwellem, C. A., Chang, J., Huang, K., . . . Ramming, A. (2024). CD200(+) fibroblasts form a pro-resolving mesenchymal network in arthritis. *Nat Immunol*, *25*(4), 682-692. https://doi.org/10.1038/s41590-024-01774-4

Regan-Komito, D., Swann, J. W., Demetriou, P., Cohen, E. S., Horwood, N. J., Sansom, S. N., & Griseri, T. (2020). GM-CSF drives dysregulated hematopoietic stem cell activity and pathogenic extramedullary myelopoiesis in experimental spondyloarthritis. *Nat Commun*, *11*(1), 155. https://doi.org/10.1038/s41467-019-13853-4

Sakaguchi, N., Takahashi, T., Hata, H., Nomura, T., Tagami, T., Yamazaki, S., Sakihama, T., Matsutani, T., Negishi, I., Nakatsuru, S., & Sakaguchi, S. (2003). Altered thymic T-cell selection due to a mutation of the ZAP-70 gene causes autoimmune arthritis in mice. *Nature*, *426*(6965), 454-460. https://doi.org/10.1038/nature02119

Schett, G., Lories, R. J., D'Agostino, M. A., Elewaut, D., Kirkham, B., Soriano, E. R., & McGonagle, D. (2017). Enthesitis: from pathophysiology to treatment. *Nat Rev Rheumatol*, *13*(12), 731-741. https://doi.org/10.1038/nrrheum.2017.188

Shaw, A. T., Maeda, Y., & Gravallese, E. M. (2016). IL-17A deficiency promotes periosteal bone formation in a model of inflammatory arthritis. *Arthritis Res Ther*, *18*(1), 104. https://doi.org/10.1186/s13075-016-0998-x

Signoretto, I., Calzetti, F., Finotti, G., Lonardi, S., Balanzin, C., Bianchetto-Aguilera, F., Gasperini, S., Gardiman, E., Castellucci, M., Russignan, A., Bonifacio, M., Sica, A., Vermi, W., Tecchio, C., Scapini, P., Tamassia, N., & Cassatella, M. A. (2025). Uncovering two neutrophil-committed progenitors that immediately precede promyelocytes during human neutropoiesis. *Cell Mol Immunol*, *22*(3), 316-329. https://doi.org/10.1038/s41423-025-01259-w

Srivastava, M. K., Sinha, P., Clements, V. K., Rodriguez, P., & Ostrand-Rosenberg, S. (2010). Myeloid-derived suppressor cells inhibit T-cell activation by depleting cystine and cysteine. *Cancer Res*, *70*(1), 68-77. https://doi.org/10.1158/0008-5472.CAN-09-2587

Wang, W., Jiao, Z., Duan, T., Liu, M., Zhu, B., Zhang, Y., Xu, Q., Wang, R., Xiong, Y., Xu, H., & Lu, L. (2015). Functional characterization of myeloid-derived suppressor cell subpopulations during the development of experimental arthritis. *Eur J Immunol*, *45*(2), 464-473. https://doi.org/10.1002/eji.201444799

Wipke, B. T., & Allen, P. M. (2001). Essential role of neutrophils in the initiation and progression of a murine model of rheumatoid arthritis. *J Immunol*, *167*(3), 1601-1608. https://doi.org/10.4049/jimmunol.167.3.1601

Wood, M. K., Daoud, A., Talor, M. V., Kalinoski, H. M., Hughes, D. M., Jaime, C. M., Hooper, J. E., Won, T., & Cihakova, D. (2024). Programmed Death Ligand 1-Expressing Macrophages and Their Protective Role in the Joint During Arthritis. *Arthritis Rheumatol*, *76*(4), 553-565. https://doi.org/10.1002/art.42749

Yan, L., Liang, M., Yang, T., Ji, J., Jose Kumar Sreena, G. S., Hou, X., Cao, M., & Feng, Z. (2020). The Immunoregulatory Role of Myeloid-Derived Suppressor Cells in the Pathogenesis of Rheumatoid Arthritis. *Front Immunol*, *11*, 568362. https://doi.org/10.3389/fimmu.2020.568362

Response to Reviewers

CD200⁺ fibroblasts prime the immunoregulatory function of granulocytic myeloid-derived suppressor cells in inflammatory arthritis

We thank the reviewers again for their thoughtful evaluation of our manuscript. Below, we provide point-by-point responses to the final comments. Reviewer comments are in *italics* and our responses follow in regular text. Changes to the manuscript are marked as “track changes”.

Reviewer #1

Comment 1

> *"In the biopsy technique published by Egund et al., the biopsy needle penetrates the SI joints at a perpendicular angle, thereby yielding tissue from subchondral bone marrow as well as the iliac and sacral bone plates. The authors explain that the biopsy site was chosen based on the presence of bone marrow edema on MRI. This suggests that the subchondral bone marrow was targeted rather than the SI joint. Please state explicitly which tissue(s) were sampled and reconcile this with the MRI-based site-selection criterion. An example CT image of the placed biopsy needle would be highly informative for the reader.”*

Response: We thank the reviewer for this comment and have clarified both points in the Methods. Active sacroiliitis is defined by bone marrow edema (BME) in the subchondral or periarticular bone marrow of the sacroiliac joint (Lambert et al., 2016), so subchondral BME is the imaging correlate of joint involvement rather than of a process remote from the joint. MRI identified the region of subchondral BME and the biopsy was performed under CT guidance at the same site, following the technique described by Egund et al. (2020). The Methods now state explicitly that the tissue samples comprised joint cartilage and the underlying subchondral bone at the site of BME.

Comment 2

> *"The animal data supporting the CD200-CD200R-G-MDSC axis are compelling. However, I remain unconvinced that the human data (tissue CD200 levels, circulating G-MDSC numbers) establish the same relationship in axSpA - the rebuttal and revised discussion do not fully resolve this. I would ask that the discussion states this limitation more plainly: that the human observations are consistent with, but do not establish, the mechanism demonstrated in mice. This is a question the field will need to resolve going forward.*

Response: We fully agree with the reviewer and have stated this limitation explicitly. Our human data demonstrate that $CD200^+$ fibroblasts and PD-$L1^+$ G-MDSCs are spatially associated and that their abundance relates to disease activity, but these observations are correlative and cannot finally establish causality in patients. We have added a dedicated limitation paragraph to the discussion that makes this distinction explicit and identifies it as an open question for the field.

Comment 3

> *"For clarity, I suggest the following change in the abstract: "CD200R agonists can restore PD-L1 expression in G-MDSCs and control axial and peripheral joint inflammation in vivo, whereas…" => "CD200R agonists can restore PD-L1 expression in G-MDSCs and control axial and peripheral joint inflammation in vivo in mice, whereas…" The addition of "in mice" is critical, as the previous sentence in the abstract describes findings in human axSpA while the mechanistic data are from mice, which may or may not apply to human disease."*

Response: We thank the reviewer for this suggestion and fully agree that the distinction between human and murine findings must be unambiguous in the abstract. The abstract has been revised and the mouse model is now named explicitly for each *in vivo* finding, including the CD200R agonist experiments.

Reviewer #2

The authors of this manuscript have done a commendable job with their revision, and successfully addressed my concerns.

I still feel that they should report the Treg cell frequencies at the end of their suppression assay, especially since they report that Treg cells are included in the assay based on the kit they use for CD4+ T cell isolation. This is an important point considering the dominant suppression that Treg cells may exert.

Response: We thank the reviewer for the positive assessment of our revision and for pursuing this point. We agree that the efficiency of T_{Reg} depletion should be documented rather than assumed from the manufacturer's specification. We have therefore assessed the frequency of $CD4^+CD25^+$ cells among $CD4^+$ splenocytes before and after isolation of naïve $CD4^+CD25^-$ T cells by flow cytometry prior to co-culture. $CD25^+$ cells accounted for 0.12% of the isolated $CD4^+$ T cell population, compared to 10.8% prior to isolation, confirming efficient depletion of regulatory T cells during negative selection with the Naïve $CD4^+$ T Cell Isolation Kit (revised Figure S2E). We would additionally like to note that all conditions within a given assay derive from the same T cell isolation split across wells, so that any residual $CD25^+$ cells are present at identical frequency in every condition, including the activated control and cannot account for the differences observed between conditions.

Reviewer #3

"I thank the authors for a their comprehensive resposne to my comments and the revision of the manuscript. In particular I think the additonal experiments elucidating the mechanistic role of PDL1 improve the paper. I take on board the explanation rgearding the re-analysis of the data from the K/BxN serum transfer arthritis model by Grieshaber-Bouyer et al, but given that this model to my knowledge does not feature axial arhtritis and this is fundamentally a paper about axial inflammation I am still struggling with the fact this data features prominently in 3 out of the 6 main figures in the paper."

Response: We thank the reviewer for the positive assessment and for returning to this point. Our earlier response explained why we used existing datasets for discovery rather than generating new sequencing data from our own model and we continue to hold that view. The comment raises a distinct point, the prominence of these analyses in the main figures and we have addressed it.

One clarification first, as it determined what we moved. GSE165276 is a cross-tissue neutrophil atlas rather than a K/BxN dataset. K/BxN blood and joint are two of five conditions, alongside IL-1β pneumonitis, IL-1β peritonitis and healthy blood. Every analysis we show from this dataset UMAP clustering, marker gene expression, neutrotime scoring and trajectory inference) is performed across all five conditions, and the *Hmgb2*$^{+}$ precursor state is therefore not a feature of serum transfer arthritis. It is a property of the neutrophil compartment that we identified across anatomically and etiologically distinct inflammations and subsequently tested in the axial model. We have retained these analyses as Figure 3 and revised the text so that the cross-compartment nature of the dataset is now explicit in the section heading, in the description of the dataset and in the concluding sentence of that section.

The K/BxN-specific analyses have been demoted to the Supplementary Information:

1. Whole-tissue joint dataset from K/BxN serum transfer arthritis (Figure 4A-D) → Supplementary Figure 3
2. Adoptive transfer of polarized mature G-MDSCs in serum transfer arthritis (Figure 6B-D) → Supplementary Figure 5B-E

The corresponding Results section was shortened and is now explicitly framed as hypothesis generation.

Finally, no claim rests on either dataset. All functional data (CD200R1 agonist treatment, anti-CD200 blockade, PD-L1^{+} G-MDSC abundance and spondylitis severity) derive from hTNFtg/*Il23mc* mice, supported by translational data from human sacroiliac joint tissue. The only serum transfer experiment retained in the main figures is the adoptive transfer of CD177^{+}Ly6G^{+} precursors (new Figure 6B-E). It demonstrates a general property of these precursors: when less CD200 is available, fewer of them polarize into PD-L1^{+} G-MDSCs. This is a cell-intrinsic mechanism and does not depend on the model in which the transfer is performed.

References

Egund, N., Sorensen, F. B., Ostgard, R., Loft, A. G., Boel, L. W. T., & Jurik, A. G. (2020). CT-guided transarticular biopsy of the sacroiliac joint: Technique and histomorphological results. A preliminary study. *Skeletal Radiol*, *49*(3), 453-460. https://doi.org/10.1007/s00256-019-03305-x

Lambert, R. G., Bakker, P. A., van der Heijde, D., Weber, U., Rudwaleit, M., Hermann, K. G., Sieper, J., Baraliakos, X., Bennett, A., Braun, J., Burgos-Vargas, R., Dougados, M., Pedersen, S. J., Jurik, A. G., Maksymowych, W. P., Marzo-Ortega, H., Ostergaard, M., Poddubnyy, D., Reijnierse, M., . . . Landewe, R. (2016). Defining active sacroiliitis on MRI for classification of axial spondyloarthritis: update by the ASAS MRI working group. *Ann Rheum Dis*, *75*(11), 1958-1963. https://doi.org/10.1136/annrheumdis-2015-208642
