# Supplementary material for: CD200+ fibroblasts prime the immunoregulatory function of granulocytic myeloid-derived suppressor cells in inflammatory arthritis
Source: Nat Commun. 2026 Oct 13;17:10746. doi: 10.1038/s41467-026-78351-w (PMC13661617; doi:10.1038/s41467-026-78351-w)
Supplement: Supplementary file 3 [file 41467_2026_78351_MOESM3_ESM.pdf]

nature portfolio

Corresponding author(s): Andreas Ramming

Last updated by author(s): Aug 31, 2026

# Reporting Summary

Nature Portfolio wishes to improve the reproducibility of the work that we publish. This form provides structure for consistency and transparency in reporting. For further information on Nature Portfolio policies, see our Editorial Policies and the Editorial Policy Checklist.

## Statistics

For all statistical analyses, confirm that the following items are present in the figure legend, table legend, main text, or Methods section.

| n/a | Confirmed | |
|---|---|---|
| ☐ | ☒ | The exact sample size (*n*) for each experimental group/condition, given as a discrete number and unit of measurement |
| ☐ | ☒ | A statement on whether measurements were taken from distinct samples or whether the same sample was measured repeatedly |
| ☐ | ☒ | The statistical test(s) used AND whether they are one- or two-sided<br>*Only common tests should be described solely by name; describe more complex techniques in the Methods section.* |
| ☒ | ☐ | A description of all covariates tested |
| ☐ | ☒ | A description of any assumptions or corrections, such as tests of normality and adjustment for multiple comparisons |
| ☐ | ☒ | A full description of the statistical parameters including central tendency (e.g. means) or other basic estimates (e.g. regression coefficient) AND variation (e.g. standard deviation) or associated estimates of uncertainty (e.g. confidence intervals) |
| ☐ | ☒ | For null hypothesis testing, the test statistic (e.g. *F*, *t*, *r*) with confidence intervals, effect sizes, degrees of freedom and *P* value noted<br>*Give P values as exact values whenever suitable.* |
| ☒ | ☐ | For Bayesian analysis, information on the choice of priors and Markov chain Monte Carlo settings |
| ☒ | ☐ | For hierarchical and complex designs, identification of the appropriate level for tests and full reporting of outcomes |
| ☒ | ☐ | Estimates of effect sizes (e.g. Cohen's *d*, Pearson's *r*), indicating how they were calculated |

*Our web collection on statistics for biologists contains articles on many of the points above.*

## Software and code

Policy information about availability of computer code

| | |
|---|---|
| Data collection | MRI volumetric data were analyzed with 3D Slicer v. 5.6.1; histology analysis was done with NDPView2 v .2.7.39 or Aperio ImageScope v12.4.3.7001; For Immunoflourescence Imaging analysis Leica Application Suite X (v3.7.423463) and Fiji (v. 1.52) was used; for cell sorting on BD Astrios Summit v 6.3.1 was used; for acquisition of cytometry data, Beckman Coulter Gallios software v 1.2, CytExpert v2.2 or Cytek Biosciences SpectroFlo 3.1.0 or was used; for flow cytometry data analysis FlowJo v. 10.10 or Kaluza 2.1, BD Biosciences was used;<br><br>Single-cell RNA sequencing data were analyzed using R 4.3.2 and Python 3.9.18. The following packages and pipelines were used: Seurat (v5.1.0), Scanpy (v1.9.8), scVI-tools (v1.1.1), UCell (v2.6.2), clusterProfiler (v4.10.0), Slingshot (v2.10.0), dittoSeq (v1.14.2), Harmony (0.1.0) CellChat (v2.1.2), phateR (v1.0.7), CytoTRACE (v0.3.3), CytoTRACE 2 (v1.1.0) and the Leiden clustering algorithm., |
| Data analysis | No custom software was developed; the use of any software is mentioned in the material and methods section including version numbers. |

For manuscripts utilizing custom algorithms or software that are central to the research but not yet described in published literature, software must be made available to editors and reviewers. We strongly encourage code deposition in a community repository (e.g. GitHub). See the Nature Portfolio guidelines for submitting code & software for further information.

## Data

Policy information about availability of data

All manuscripts must include a data availability statement. This statement should provide the following information, where applicable:

- Accession codes, unique identifiers, or web links for publicly available datasets
- A description of any restrictions on data availability
- For clinical datasets or third party data, please ensure that the statement adheres to our policy

No new sequencing datasets were generated in this study. The previously published datasets analysed here are cited in the Methods with their accession codes and references: GSE165276, GSE253974 and GSE228980. Source data are provided with this paper.

## Research involving human participants, their data, or biological material

Policy information about studies with human participants or human data. See also policy information about sex, gender (identity/presentation), and sexual orientation and race, ethnicity and racism.

| | |
|---|---|
| Reporting on sex and gender | Gender was not considered in this study. Sex was not considered as an inclusion criterion. |
| Reporting on race, ethnicity, or other socially relevant groupings | Race, Ethnicity or socially relevent groupings were not considered in this study. |
| Population characteristics | Human research received approval from the local ethical committees of the University of Erlangen-Nürnberg (30_19B) and the Ruhr-Universität Bochum (18-6671-BR) with written informed consent obtained from all participants. Clinical assessments were conducted by experienced rheumatologists who were blinded to the study's objectives. Participants diagnosed with rheumatoid arthritis (RA) (n=27), psoriatic arthritis (PsA) (n=29) and axial spondyloarthritis (n=35) met established classification criteria (ACR, CASPAR, ASAS)35-37. Control subjects were non-arthritic individuals (n=29). Disease activity was assessed using standardized composite scores, as detailed in the respective figure captions.<br>Needle biopsies of sacroiliac joints (SIJ) were obtained under sterile conditions following patient informed consent. Prior to the biopsy, MRI was performed to assess active disease, specifically identifying regions of subchondral bone marrow edema as the target area, as this compartment constitutes the site of active sacroiliitis. The biopsy was performed under CT guidance by experienced interventional radiologists as previously described, targeting the same anatomical site identified by MRI. In line with the biopsy technique described previously, tissue samples comprised joint cartilage and the underlying subchondral bone at the site of BME. Local anesthesia was applied and needle placement was confirmed by CT imaging prior to sample collection.<br>Biopsies of vertebral facet joints were taken under sterile conditions upon patient´s informed consent during spinal surgery. The region of interest had been verified prior by MRI as inflamed for axSpA patients and as disease-free for the controls.<br><br>Patients characteristics are provided in Table 1, 2, and Table S1<br><br>Sex was recorded from clinical records and is reported in all three tables. Sex was not used as an inclusion criterion, and the cohorts were not powered for a sex-disaggregated analysis. Race, ethnicity and other socially relevant groupings were not considered. |
| Recruitment | Participants were recruited consecutively from the rheumatology outpatient clinics of the two participating centres (Universitätsklinikum Erlangen and Rheumazentrum Ruhrgebiet, Herne) during routine clinical care. Axial tissue was obtained from patients undergoing CT-guided sacroiliac joint biopsy or spinal surgery for clinical indications, with degenerative controls recruited from the same surgical setting. Recruitment at tertiary rheumatology centres biases the cohorts towards established, treated disease and healthy controls were younger than the patient groups (Table 1). These factors do not affect the within-cohort correlations reported here. Participants received no compensation. |
| Ethics oversight | Human research was approved by the ethics committees of the Friedrich-Alexander-Universität Erlangen-Nürnberg (30_19B) and the Ruhr-Universität Bochum (18-6671-BR). Written informed consent was obtained from all participants. |

Note that full information on the approval of the study protocol must also be provided in the manuscript.

# Field-specific reporting

Please select the one below that is the best fit for your research. If you are not sure, read the appropriate sections before making your selection.

☒ Life sciences ☐ Behavioural & social sciences ☐ Ecological, evolutionary & environmental sciences

For a reference copy of the document with all sections, see nature.com/documents/nr-reporting-summary-flat.pdf

# Life sciences study design

All studies must disclose on these points even when the disclosure is negative.

| | |
|---|---|
| Sample size | No statistical methods were used to pre-specify sample sizes, but our sample sizes are similar to those reported in previous publications. |

| | |
|---|---|
| Data exclusions | No data points were excluded from the analyses. In individual experiments, single samples could not be analysed owing to insufficient cell yield after enzymatic tissue digestion; the exact n is given for each panel in the figure legends. |
| Replication | The key findings in the study were reliably reproduced in independent cohorts. Key findings were reproduced by the use of different experimental models or public online data sets. |
| Randomization | Animals were randomized by sex. All animals were 6-10 weeks old at the start of the experiment. |
| Blinding | Mice were stratified evenly by sex and then randomly assigned to experimental groups. All animals were 8–10 weeks old at the start of the experiments. For the human cohorts, randomization is not applicable: participants were assigned to groups by their clinical diagnosis, established according to the respective classification criteria. |

# Reporting for specific materials, systems and methods

We require information from authors about some types of materials, experimental systems and methods used in many studies. Here, indicate whether each material, system or method listed is relevant to your study. If you are not sure if a list item applies to your research, read the appropriate section before selecting a response.

## Materials & experimental systems

| n/a | Involved in the study | |
|---|---|---|
| ☐ | ☒ | Antibodies |
| ☒ | ☐ | Eukaryotic cell lines |
| ☒ | ☐ | Palaeontology and archaeology |
| ☐ | ☒ | Animals and other organisms |
| ☐ | ☒ | Clinical data |
| ☒ | ☐ | Dual use research of concern |
| ☒ | ☐ | Plants |

## Methods

| n/a | Involved in the study | |
|---|---|---|
| ☒ | ☐ | ChIP-seq |
| ☐ | ☒ | Flow cytometry |
| ☒ | ☐ | MRI-based neuroimaging |

# Antibodies

Antibodies used

| Application | Antigen | Label | Supplier | Clone | Cat# | Dilution |
|---|---|---|---|---|---|---|
| FC-Mo | CD45 | AlexaFluor700 | BioLegend | 30-F11 | 109822 | 1:1000 |
| FC-Mo | CD45 | APC/Cy7 | BioLegend | 30-F11 | 103115 | 1:2000 |
| FC-Mo | CD3e | FITC | BioLegend | 145-2C11 | 100306 | 1:100 |
| FC-Mo | CD3e | BV421 | BioLegend | 145-2C11 | 100341 | 1:100 |
| FC-Mo | CD3 | APC/Cy7 | BioLegend | 17A2 | 100222 | 1:100 |
| FC-Mo | CD45R/B220 | FITC | BioLegend | RA3-6B2 | 103206 | 1:500 |
| FC-Mo | CD45R/B220 | APC/Cy7 | BioLegend | RA3-6B2 | 103224 | 1:500 |
| FC-Mo | CD49b | FITC | BioLegend | DX5 | 108906 | 1:100 |
| FC-Mo | CD11c | FITC | BioLegend | N418 | 117306 | 1:100 |
| FC-Mo | CD11c | APC/Cy7 | BioLegend | N418 | 117324 | 1:100 |
| FC-Mo | CD170/SiglecF | FITC | BioLegend | S17007L | 155504 | 1:50 |
| FC-Mo | CD170/SiglecF | APC/Cy7 | BioLegend | S17007L | 155532 | 1:100 |
| FC-Mo | Ly6G | BV421 | BioLegend | 1A8 | 127641 | 1:100 |
| FC-Mo | Ly6G | BV421 | BioLegend | 1A8 | 127628 | 1:500 |
| FC-Mo | Ly6G | FITC | BioLegend | 1A8 | 127606 | 1:100 |
| FC-Mo | CD177 | AlexaFluor647 | BD Biosciences | Y127 | 566599 | 1:100 |
| FC-Mo | CD11b | APC | BioLegend | M1/70 | 101212 | 1:1000 |
| FC-Mo | CD11b | APC | BioLegend | M1/70 | 101212 | 1:500 |
| FC-Mo | CD11b | BV510 | BioLegend | M1/70 | 101263 | 1:1000 |
| FC-Mo | Ly6C | BV510 | BioLegend | HK1.4 | 128033 | 1:100 |
| FC-Mo | CD182/CXCR2 | PE | BioLegend | SA044G4 | 149304 | 1:100 |
| FC-Mo | CD101 | PE/Cy7 | Thermo Fisher | Moushi101 | 25-1011-82 | 1:100 |
| FC-Mo | CD274/PD-L1 | PE | BioLegend | 10F.9G2 | 124308 | 1:100 |
| FC-Mo | CD25 | PE | BioLegend | 3C7 | 101904 | 1:250 |
| FC-Mo | CD90.2 | PerCP/Cy5.5 | BioLegend | 30-H12 | 105338 | 1:500 |
| FC-Mo | CD4 | PE/Cy7 | BioLegend | GK1.5 | 100422 | 1:1500 |
| FC-Mo | CD4 | APC/Cy7 | BioLegend | RM4.5 | 100526 | 1:1000 |
| FC-Mo | TCR-beta | APC | BioLegend | H57-597 | 109212 | 1:500 |
| FC-Mo | F4/80 | PE/Cy7 | BioLegend | BM8 | 123113 | 1:100 |
| FC-Mo | CD200R1 | AlexaFluor647 | BD Biosciences | OX-110 | 566345 | 1:200 |
| FC-Mo | CD16/32 (Fc block) | unconjugated | BioLegend | 93 | 101302 | |
| FC-Hu | CD45 | BV510 | BioLegend | HI30 | 304036 | 1:250 |
| FC-Hu | CD45 | VioGreen | Miltenyi Biotec | 5B1 | 130-096-906 | 1:500 |
| FC-Hu | CD66b | FITC | BioLegend | G10F5 | 305104 | 1:1000 |

FC-Hu CD15 PE/Cy7 BioLegend W6D3 323030 1:1500
FC-Hu CD15 PE/Cy7 BioLegend W6D3 323030 1:50
FC-Hu CD33 APC Miltenyi Biotec AC104.3E3 130-113-345 1:50
FC-Hu HLA-DR PE BioLegend L243 307606 1:400
FC-Hu CD14 APC/Cy7 BioLegend M5E2 301820 1:50
FC-Hu CD11b Pacific Blue BioLegend ICRF44 301315 1:50
FC-Hu CD3 FITC Miltenyi Biotec UCHT1 300406 1:200
FC-Hu CD19 FITC Miltenyi Biotec HIB19 302206 1:200
FC-Hu CD56 FITC Miltenyi Biotec HCD56 318304 1:200
FC-Hu CD16 FITC Miltenyi Biotec 3G8 302006 1:200
IF-Hu CD33 unconjugated Bioss polyclonal bs-2042R 1:100
IF-Hu HLA-DR unconjugated LSBio LN3 LS-B8114 1:100
IF-Hu CD20 unconjugated BD Biosciences H1 555677 1:500
IF-Hu CD56 unconjugated Novus 123A8 NBP2-44713 1:1000
IF-Hu CD11c unconjugated Biorbyt polyclonal orb13554 1:300
IF-Hu CD3 unconjugated Abcam PS1 ab699 1:50
IF-Hu CD14 unconjugated Abcam 4B4F12 ab182032 1:100
IF-Hu CD16 unconjugated Santa Cruz 2Q1240 sc-70548 1:50
IF-Hu CD123 unconjugated BioLegend 6H6 306002 1:100
IF-Hu CD274/PD-L1 unconjugated Cell Signaling E1L3N 85164SF 1:100
IF-Hu CD200 unconjugated R&D Systems polyclonal AF2724 1:100
IF-Hu Podoplanin unconjugated R&D Systems polyclonal AF3670 1:100
IF-Mo CD182/CXCR2 unconjugated Invitrogen polyclonal PA5-100951 1:200
IF-Mo Ly6G unconjugated BioLegend 1A8 127602 1:100
IF-Mo (cryo) CD274/PD-L1 AlexaFluor647 BD Biosciences 10F.9G2 568306 1:25
IF-Mo (cryo) Ly6G AlexaFluor594 BioLegend 1A8 127636 1:100
IF-2nd Donkey anti-rabbit AlexaFluor488 Thermo Fisher polyclonal A21206 1:200
IF-2nd Donkey anti-rabbit AlexaFluor555 Abcam polyclonal ab150062 1:200
IF-2nd Donkey anti-rabbit AF555 Plus Thermo Fisher polyclonal A32794 1:500
IF-2nd Donkey anti-sheep AlexaFluor555 Thermo Fisher polyclonal A21436 1:200
IF-2nd Donkey anti-rat AF647 Plus Thermo Fisher polyclonal A48272 1:500
IF-2nd Donkey anti-mouse AlexaFluor488 Abcam polyclonal ab150105 1:200
IF-2nd Donkey anti-goat AlexaFluor647 Thermo Fisher polyclonal A-21447 1:200
IV CD200R1 Ultra-LEAF BioLegend OX-110 123917 100 ug/mouse
IV CD200 InVivoMAb BioXCell OX-90 BE0299 100 ug/mouse
IV IgG2a isotype Ultra-LEAF BioLegend RTK2758 400544 100 ug/mouse
in vitro PD-L1 blockade InVivoMAb BioXCell 10F.9G2 BE0101 10 ug/mL

Validation

All antibodies are commercially available and were validated by the manufacturers for the respective species and application; validation data are available on the manufacturers' websites for each catalogue number listed in the Methods. In addition, all flow cytometry antibodies were titrated in house and validated using fluorescence-minus-one and unstained controls on the relevant murine and human cell populations. Immunofluorescence antibodies were validated individually on positive control tissue, with secondary-antibody-only staining as negative control.

## Animals and other research organisms

Policy information about studies involving animals; ARRIVE guidelines recommended for reporting animal research, and Sex and Gender in Research

Laboratory animals

hTNFtg mice (B6.Cg-Tg(TNF)197Gkl) were maintained on a C57BL/6N background and bred in house; transgene-negative littermates served as controls. C57BL/6Rj mice were purchased from Janvier Labs. All animals were 8–10 weeks old at the start of the experiments and weighed 17–22 g. Both male and female mice were used; groups were stratified evenly by sex. Exact numbers per experiment are given in the figure legends.

Wild animals

The study did not include wild animals.

Reporting on sex

Animals were ranodmized by sex.

Field-collected samples

The study did not include field-collected samples.

Ethics oversight

Mice were maintained under specific pathogen-free conditions. All experimental procedures were conducted in accordance with local regulations and approved by the Government of Unterfranken, Würzburg (protocols 55.2-2532-2-1438, 55.2-2532-2-1886, 55.2-2532-2-1592, 55.2-2532-2-1061 and 55.2-2532-2-2337).

Note that full information on the approval of the study protocol must also be provided in the manuscript.

## Clinical data

Policy information about clinical studies

All manuscripts should comply with the ICMJE guidelines for publication of clinical research and a completed CONSORT checklist must be included with all submissions.

| | |
|---|---|
| Clinical trial registration | no trial |
| Study protocol | *Note where the full trial protocol can be accessed OR if not available, explain why.* |
| Data collection | *Describe the settings and locales of data collection, noting the time periods of recruitment and data collection.* |
| Outcomes | *Describe how you pre-defined primary and secondary outcome measures and how you assessed these measures.* |

## Plants

| | |
|---|---|
| Seed stocks | no plants |
| Novel plant genotypes | *Describe the methods by which all novel plant genotypes were produced. This includes those generated by transgenic approaches, gene editing, chemical/radiation-based mutagenesis and hybridization. For transgenic lines, describe the transformation method, the number of independent lines analyzed and the generation upon which experiments were performed. For gene-edited lines, describe the editor used, the endogenous sequence targeted for editing, the targeting guide RNA sequence (if applicable) and how the editor was applied.* |
| Authentication | *Describe any authentication procedures for each seed stock used or novel genotype generated. Describe any experiments used to assess the effect of a mutation and, where applicable, how potential secondary effects (e.g. second site T-DNA insertions, mosiacism, off-target gene editing) were examined.* |

## Flow Cytometry

### Plots

Confirm that:

- [x] The axis labels state the marker and fluorochrome used (e.g. CD4-FITC).
- [x] The axis scales are clearly visible. Include numbers along axes only for bottom left plot of group (a 'group' is an analysis of identical markers).
- [x] All plots are contour plots with outliers or pseudocolor plots.
- [x] A numerical value for number of cells or percentage (with statistics) is provided.

### Methodology

| | |
|---|---|
| Sample preparation | Single cell samples were created from joint tissue by enzymatic dissociation (Collagenase/Dispase/DNAse, outlined in methods). Spleen cells were isolated mechanically. Red cell lysis and debris removal was performed prior to staining. |
| Instrument | Samples were acquired using a Beckman Coulter Gallios (Human G-MDSC and murine CD200R1 neutrophils, Gallios Software v1.2), a Beckman Coulter Cytoflex S (Murine G-MDSC, CytExpert v2.2) or Cytek Aurora (Human Granulocytes, SpectroFlo 3.1.0) and analyzed using FlowJo v.10.10 or Kaluza 2.1. |
| Software | Gallios Software v1.2, CytExpert v2.2, SpectroFlo 3.1.0) |
| Cell population abundance | Purity of sorted populations was determined by re-analysis of the target population immediately after sorting based on the surface markers used for sorting, and exceeded 98% for each target population. |
| Gating strategy | Sequential gating strategies are shown in full in the Supplementary Information: Supplementary Fig. 1a (human granulocytes, eosinophils and MDSC subsets, related to Fig. 1b–d), Supplementary Fig. 2a (murine mature neutrophils and G-MDSCs, related to Fig. 2f), Supplementary Fig. 2e (purity of isolated naïve CD4+CD25− T cells, related to Fig. 2h), Supplementary Fig. 4n (CD200R1+ precursors, related to Fig. 3a), Supplementary Fig. 5d (PD-L1+ G-MDSCs, related to Fig. 4f–g) and Supplementary Fig. 6a and 6d (in vitro polarized G-MDSCs and adoptively transferred CFSE+ cells, related to Fig. 5a and 5e). Gate boundaries were defined using fluorescence-minus-one (FMO) or isotype controls where indicated. |

- [x] Tick this box to confirm that a figure exemplifying the gating strategy is provided in the Supplementary Information.
